# Supplementary material for: The side effect profile of Clozapine in real world data of three large mental health hospitals
Source: PLoS One. 2020 Dec 8;15(12):e0243437. doi: 10.1371/journal.pone.0243437 (PMC7723266; doi:10.1371/journal.pone.0243437)
Supplement: S1 Table — (PDF) [file pone.0243437.s001.pdf]

Clozapine - Gender Differences (%)

Measure Values
0.00 56.00

ADR	Trust	Cohort	Sub Cohort	Three Months Early	Two Months Early	One Month Early	One Month Later	Two Months Later	Three Months Later	SIDER Low End	SIDER High End
Agitation	SLAM	Trust	Clozapine (n=1760)	17.61	22.10	26.53	46.59	32.56	26.99		
		Gender	Male (n=1167)	17.31	20.31	24.68	44.56	31.71	27.34		
			Female (n=593)	18.21	25.63	30.19	50.59	34.23	26.31		
	Camden & Islington	Trust	Clozapine (n=561)	13.37	17.83	18.36	43.14	28.34	21.03		
		Gender	Male (n=357)	11.20	17.37	18.21	45.66	27.45	22.13		
			Female (n=204)	17.16	18.63	18.63	38.73	29.90	19.12		
	Oxford	Trust	Clozapine (n=514)	14.59	15.76	16.34	34.24	25.10	20.62		
		Gender	Male (n=342)	13.45	13.45	14.62	34.50	25.73	19.30		
			Female (n=172)	16.86	20.35	19.77	33.72	23.84	23.26		
	SIDER	SIDER	SIDER							4.00	
Fatigue	SLAM	Trust	Clozapine (n=1760)	12.67	14.83	15.85	43.58	35.80	30.51		
		Gender	Male (n=1167)	10.80	13.20	13.54	40.19	32.82	28.45		
			Female (n=593)	16.36	18.04	20.40	50.25	41.65	34.57		
	Camden & Islington	Trust	Clozapine (n=561)	10.34	12.30	13.37	41.18	29.23	26.56		
		Gender	Male (n=357)	8.68	10.64	11.76	38.94	29.97	24.93		
			Female (n=204)	13.24	15.20	16.18	45.10	27.94	29.41		
	Oxford	Trust	Clozapine (n=514)	9.73	11.87	12.06	35.21	27.43	26.85		
		Gender	Male (n=342)	9.06	11.40	11.99	31.87	24.85	26.32		
			Female (n=172)	11.05	12.79	12.21	41.86	32.56	27.91		
	SIDER	SIDER	SIDER								
Sedation	SLAM	Trust	Clozapine (n=1760)	12.67	12.16	14.83	43.86	35.51	29.83		
		Gender	Male (n=1167)	12.34	11.48	13.11	41.99	33.68	27.68		
			Female (n=593)	13.32	13.49	18.21	47.55	39.12	34.06		
	Camden & Islington	Trust	Clozapine (n=561)	5.17	9.09	9.09	38.15	26.56	21.93		
		Gender	Male (n=357)	5.60	8.12	8.68	40.06	26.05	23.53		
			Female (n=204)	4.41	10.78	9.80	34.80	27.45	19.12		
	Oxford	Trust	Clozapine (n=514)	7.20	8.37	9.34	31.52	21.40	18.48		
		Gender	Male (n=342)	6.14	7.89	8.77	30.99	21.35	17.54		
			Female (n=172)	9.30	9.30	10.47	32.56	21.51	20.35		
	SIDER	SIDER	SIDER							25.00	46.00
Dizziness	SLAM	Trust	Clozapine (n=1760)	2.78	4.20	4.09	16.59	13.13	11.19		
		Gender	Male (n=1167)	2.06	3.43	3.51	14.31	11.91	10.37		
			Female (n=593)	4.22	5.73	5.23	21.08	15.51	12.82		
	Camden & Islington	Trust	Clozapine (n=561)	3.21	3.39	3.74	18.18	13.73	9.09		
		Gender	Male (n=357)	2.52	3.08	5.04	15.97	11.48	8.12		
			Female (n=204)	4.41	3.92	1.47	22.06	17.65	10.78		
	Oxford	Trust	Clozapine (n=514)	3.89	4.09	4.47	17.70	13.04	10.12		
		Gender	Male (n=342)	2.63	3.22	3.22	14.91	11.11	8.48		
			Female (n=172)	6.40	5.81	6.98	23.26	16.86	13.37		
	SIDER	SIDER	SIDER							12.00	27.00
Hypersalivation	SLAM	Trust	Clozapine (n=1760)	1.19	1.48	2.10	14.32	13.24	11.31		
		Gender	Male (n=1167)	1.46	2.14	2.57	15.25	12.60	10.80		
			Female (n=593)	0.67	0.17	1.18	12.48	14.50	12.31		
	Camden & Islington	Trust	Clozapine (n=561)	1.07	1.43	0.53	14.26	6.95	7.66		
		Gender	Male (n=357)	1.40	1.96	0.56	14.57	7.84	6.72		
			Female (n=204)	0.49	0.49	0.49	13.73	5.39	9.31		
	Oxford	Trust	Clozapine (n=514)	0.97	0.78	1.56	12.65	10.70	5.84		
		Gender	Male (n=342)	0.88	0.29	1.17	10.82	10.23	4.68		
			Female (n=172)	1.16	1.74	2.33	16.28	11.63	8.14		
	SIDER	SIDER	SIDER							1.00	48.00
Feelingsick	SLAM	Trust	Clozapine (n=1760)	4.66	4.94	6.48	14.32	11.19	9.09		
		Gender	Male (n=1167)	4.37	4.54	6.43	11.31	8.83	7.28		
			Female (n=593)	5.23	5.73	6.58	20.24	15.85	12.65		
	Camden & Islington	Trust	Clozapine (n=561)	3.74	3.92	3.03	10.52	7.13	7.66		
		Gender	Male (n=357)	3.64	3.92	3.36	10.08	6.16	7.84		
			Female (n=204)	3.92	3.92	2.45	11.27	8.82	7.35		
	Oxford	Trust	Clozapine (n=514)	3.89	5.25	5.06	14.20	9.73	7.20		
		Gender	Male (n=342)	3.80	3.80	3.80	13.16	8.77	5.56		
			Female (n=172)	4.07	5.81	7.56	16.28	11.63	10.47		
	SIDER	SIDER	SIDER								
Weightgain	SLAM	Trust	Clozapine (n=1760)	3.75	4.43	5.06	15.34	10.91	10.34		
		Gender	Male (n=1167)	3.77	4.46	4.54	13.45	9.08	9.17		
			Female (n=593)	3.71	4.38	6.07	19.06	14.50	12.65		
	Camden & Islington	Trust	Clozapine (n=561)	2.50	3.39	1.96	11.76	6.60	6.24		
		Gender	Male (n=357)	2.52	2.80	1.96	10.08	4.76	6.44		
			Female (n=204)	2.45	4.41	1.96	14.71	9.80	5.88		

The results are shown in percentages (%) and broken down by ADRs, Trusts (SLAM, Camden & Islington and Oxford), Cohorts, Sub Cohorts and SIDER reported values.
In Sub Cohort 'Clozapine' represent the total baseline population which further breaks down into Gender 'Male' and 'Female' groups.
The columns (Three Months Early, Two Months Early, One Month Early, One Month Later, Two Months Later, Three Months Later) shows the percentages in each monthly interval. The last two columns (SIDER Low End and SIDER High End) shows the SIDER reporting.

Clozapine - Gender Differences (%)

Measure Values 0.00 – 56.00

ADR	Trust	Cohort	Sub Cohort	Three Months Early	Two Months Early	One Month Early	One Month Later	Two Months Later	Three Months Later	SIDER Low End	SIDER High End
Weightgain	Oxford	Trust	Clozapine (n=514)	3.50	3.31	3.70	11.28	9.92	7.78		
		Gender	Male (n=342)	3.51	3.22	3.22	10.53	8.77	7.31		
			Female (n=172)	3.49	3.49	4.65	12.79	12.21	8.72		
	SIDER	SIDER	SIDER							4.00	56.00
Tachycardia	SLAM	Trust	Clozapine (n=1760)	2.27	2.05	2.50	15.40	12.95	9.94		
		Gender	Male (n=1167)	2.40	1.80	2.23	16.28	13.11	10.37		
			Female (n=593)	2.02	2.53	3.04	13.66	12.65	9.11		
	Camden & Islington	Trust	Clozapine (n=561)	1.43	1.43	0.89	11.23	8.38	6.95		
		Gender	Male (n=357)	0.84	1.12	1.12	11.20	8.40	7.28		
			Female (n=204)	2.45	1.96	0.49	11.27	8.33	6.37		
	Oxford	Trust	Clozapine (n=514)	0.78	1.36	1.56	10.89	10.51	7.59		
		Gender	Male (n=342)	0.88	1.17	1.17	10.82	10.53	7.31		
			Female (n=172)	0.58	1.74	2.33	11.05	10.47	8.14		
	SIDER	SIDER	SIDER							11.00	25.00
Confusion	SLAM	Trust	Clozapine (n=1760)	4.72	5.51	6.08	13.92	8.47	6.76		
		Gender	Male (n=1167)	4.46	5.40	6.34	13.37	8.40	7.11		
			Female (n=593)	5.23	5.73	5.56	15.01	8.60	6.07		
	Camden & Islington	Trust	Clozapine (n=561)	3.57	6.24	5.53	12.66	6.77	5.88		
		Gender	Male (n=357)	3.92	6.72	5.32	13.45	7.84	7.28		
			Female (n=204)	2.94	5.39	5.88	11.27	4.90	3.43		
	Oxford	Trust	Clozapine (n=514)	2.53	3.89	3.89	9.92	6.42	5.25		
		Gender	Male (n=342)	2.05	2.63	2.92	8.19	6.14	4.09		
			Female (n=172)	3.49	6.40	5.81	13.37	6.98	7.56		
	SIDER	SIDER	SIDER							3.00	
Constipation	SLAM	Trust	Clozapine (n=1760)	1.76	1.99	2.16	12.27	11.70	9.49		
		Gender	Male (n=1167)	1.11	1.54	1.63	10.45	9.17	8.23		
			Female (n=593)	3.04	2.87	3.20	15.85	16.69	11.97		
	Camden & Islington	Trust	Clozapine (n=561)	1.07	2.50	1.78	11.41	7.13	5.70		
		Gender	Male (n=357)	0.56	2.80	1.68	10.36	7.00	3.92		
			Female (n=204)	1.96	1.96	1.96	13.24	7.35	8.82		
	Oxford	Trust	Clozapine (n=514)	0.58	0.97	1.36	10.31	7.78	7.78		
		Gender	Male (n=342)	0.29	0.88	1.17	8.77	6.43	6.43		
			Female (n=172)	1.16	1.16	1.74	13.37	10.47	10.47		
	SIDER	SIDER	SIDER							10.00	25.00
Headache	SLAM	Trust	Clozapine (n=1760)	4.20	4.55	5.45	12.44	8.18	5.91		
		Gender	Male (n=1167)	3.51	3.26	4.46	10.45	7.46	5.31		
			Female (n=593)	5.56	7.08	7.42	16.36	9.61	7.08		
	Camden & Islington	Trust	Clozapine (n=561)	2.32	3.57	4.28	9.27	6.42	4.63		
		Gender	Male (n=357)	2.24	3.64	3.64	10.08	6.72	4.48		
			Female (n=204)	2.45	3.43	5.39	7.84	5.88	4.90		
	Oxford	Trust	Clozapine (n=514)	3.89	3.89	4.09	10.89	8.37	7.59		
		Gender	Male (n=342)	3.51	3.80	4.09	11.11	8.77	7.31		
			Female (n=172)	4.65	4.07	4.07	10.47	7.56	8.14		
	SIDER	SIDER	SIDER								
Insomnia	SLAM	Trust	Clozapine (n=1760)	3.92	4.03	5.17	10.40	6.48	4.03		
		Gender	Male (n=1167)	3.68	4.03	4.46	9.85	6.00	4.54		
			Female (n=593)	4.38	4.05	6.58	11.47	7.42	3.04		
	Camden & Islington	Trust	Clozapine (n=561)	3.57	3.39	3.74	8.91	3.39	4.28		
		Gender	Male (n=357)	3.64	3.08	4.20	10.08	2.80	3.36		
			Female (n=204)	3.43	3.92	2.94	6.86	4.41	5.88		
	Oxford	Trust	Clozapine (n=514)	5.84	4.86	5.84	8.37	6.81	4.09		
		Gender	Male (n=342)	4.68	3.51	4.39	5.85	5.85	3.51		
			Female (n=172)	8.14	7.56	8.72	13.37	8.72	5.23		
	SIDER	SIDER	SIDER							20.00	33.00
Hyperprolactinaemia	SLAM	Trust	Clozapine (n=1760)	3.18	3.64	4.20	8.52	5.06	4.15		
		Gender	Male (n=1167)	2.66	2.49	2.91	6.43	3.86	3.68		
			Female (n=593)	4.22	5.90	6.75	12.65	7.42	5.06		
	Camden & Islington	Trust	Clozapine (n=561)	1.60	1.78	2.67	8.20	4.10	3.57		
		Gender	Male (n=357)	0.84	0.56	1.96	6.44	2.52	2.52		
			Female (n=204)	2.94	3.92	3.92	11.27	6.86	5.39		
	Oxford	Trust	Clozapine (n=514)	3.70	4.09	4.28	8.75	4.47	4.86		
		Gender	Male (n=342)	2.92	2.34	2.63	7.60	3.51	3.80		
			Female (n=172)	5.23	7.56	7.56	11.05	6.40	6.98		
	SIDER	SIDER	SIDER								
Shaking	SLAM	Trust	Clozapine (n=1760)	3.13	2.95	3.92	9.55	5.40	5.06		
		Gender	Male (n=1167)	2.57	3.00	3.68	9.08	5.23	5.40		

The results are shown in percentages (%) and broken down by ADRs, Trusts (SLAM, Camden & Islington and Oxford), Cohorts, Sub Cohorts and SIDER reported values.
In Sub Cohort 'Clozapine' represent the total baseline population which further breaks down into Gender 'Male' and 'Female' groups.
The columns (Three Months Early, Two Months Early, One Month Early, One Month Later, Two Months Later, Three Months Later) shows the percentages in each monthly interval. The last two columns (SIDER Low End and SIDER High End) shows the SIDER reporting.

Clozapine - Gender Differences (%)

Measure Values: 0.00 – 56.00

ADR	Trust	Cohort	Sub Cohort	Three Months Early	Two Months Early	One Month Early	One Month Later	Two Months Later	Three Months Later	SIDER Low End	SIDER High End
**Shaking**	SLAM	Gender	Female (n=593)	4.22	2.87	4.38	10.46	5.73	4.38		
	Camden & Islington	Trust	Clozapine (n=561)	1.78	1.96	3.74	6.06	3.92	2.85		
		Gender	Male (n=357)	1.96	1.96	4.48	6.16	3.92	3.36		
			Female (n=204)	1.47	1.96	2.45	5.88	3.92	1.96		
	Oxford	Trust	Clozapine (n=514)	2.92	3.31	3.89	7.78	4.47	4.86		
		Gender	Male (n=342)	2.05	2.92	2.92	7.31	4.39	3.22		
			Female (n=172)	4.65	4.07	5.81	8.72	4.65	8.14		
	SIDER	SIDER	SIDER								
**Vomiting**	SLAM	Trust	Clozapine (n=1760)	2.56	2.50	3.01	8.86	6.82	5.00		
		Gender	Male (n=1167)	1.97	2.14	3.34	8.57	5.91	4.46		
			Female (n=593)	3.71	3.20	2.36	9.44	8.60	6.07		
	Camden & Islington	Trust	Clozapine (n=561)	2.14	2.50	2.85	6.77	4.99	4.63		
		Gender	Male (n=357)	2.24	2.52	1.40	6.72	4.20	3.64		
			Female (n=204)	1.96	2.45	5.39	6.86	6.37	6.37		
	Oxford	Trust	Clozapine (n=514)	1.75	2.72	2.92	7.59	5.25	5.06		
		Gender	Male (n=342)	2.05	2.34	2.05	7.89	4.09	4.97		
			Female (n=172)	1.16	3.49	4.65	6.98	7.56	5.23		
	SIDER	SIDER	SIDER							3.00	17.00
**Hypertension**	SLAM	Trust	Clozapine (n=1760)	2.05	2.22	3.13	9.15	5.74	4.60		
		Gender	Male (n=1167)	1.80	1.46	2.49	8.48	5.40	4.54		
			Female (n=593)	2.53	3.71	4.38	10.46	6.41	4.72		
	Camden & Islington	Trust	Clozapine (n=561)	0.71	0.71	1.60	7.13	4.63	2.67		
		Gender	Male (n=357)	0.28	0.28	0.84	6.72	5.04	2.80		
			Female (n=204)	1.47	1.47	2.94	7.84	3.92	2.45		
	Oxford	Trust	Clozapine (n=514)	1.36	1.36	1.56	5.06	4.28	2.14		
		Gender	Male (n=342)	0.88	0.88	0.58	5.56	3.80	2.34		
			Female (n=172)	2.33	2.33	3.49	4.07	5.23	1.74		
	SIDER	SIDER	SIDER							4.00	12.00
**Abdominalpain**	SLAM	Trust	Clozapine (n=1760)	1.88	1.99	2.56	8.01	6.02	4.72		
		Gender	Male (n=1167)	1.80	1.97	2.31	7.97	6.00	4.20		
			Female (n=593)	2.02	2.02	3.04	8.09	6.07	5.73		
	Camden & Islington	Trust	Clozapine (n=561)	0.89	0.89	1.60	3.92	3.57	3.39		
		Gender	Male (n=357)	0.28	1.40	1.96	3.64	3.36	3.08		
			Female (n=204)	0.98	0.00	0.98	4.41	3.92	3.92		
	Oxford	Trust	Clozapine (n=514)	1.75	1.36	1.75	7.39	4.47	5.64		
		Gender	Male (n=342)	1.46	0.88	1.17	7.60	4.68	4.39		
			Female (n=172)	2.33	2.33	2.91	6.98	4.07	8.14		
	SIDER	SIDER	SIDER							4.00	
**Backache**	SLAM	Trust	Clozapine (n=1760)	1.14	1.59	2.44	4.94	3.35	2.73		
		Gender	Male (n=1167)	0.94	1.29	1.97	4.03	2.83	2.57		
			Female (n=593)	1.52	2.19	3.37	6.75	4.38	3.04		
	Camden & Islington	Trust	Clozapine (n=561)	1.43	1.25	1.96	5.35	3.03	3.03		
		Gender	Male (n=357)	0.84	1.40	2.24	3.08	2.24	1.96		
			Female (n=204)	2.45	0.98	1.47	9.31	4.41	4.90		
	Oxford	Trust	Clozapine (n=514)	1.17	1.17	1.36	5.84	3.89	2.92		
		Gender	Male (n=342)	0.88	0.88	1.17	3.80	3.22	1.75		
			Female (n=172)	1.74	1.74	1.74	9.88	5.23	5.23		
	SIDER	SIDER	SIDER							5.00	
**Nausea**	SLAM	Trust	Clozapine (n=1760)	1.14	1.08	1.19	6.08	5.23	3.69		
		Gender	Male (n=1167)	1.03	0.86	1.29	5.06	4.54	2.66		
			Female (n=593)	1.35	1.52	1.01	8.09	6.58	5.73		
	Camden & Islington	Trust	Clozapine (n=561)	0.89	1.43	0.36	4.63	3.57	3.57		
		Gender	Male (n=357)	0.00	1.40	0.28	3.92	2.80	2.80		
			Female (n=204)	2.45	1.47	0.49	5.88	4.90	4.90		
	Oxford	Trust	Clozapine (n=514)	0.97	0.58	1.36	4.86	3.70	2.92		
		Gender	Male (n=342)	0.58	0.58	0.58	3.51	3.22	2.34		
			Female (n=172)	1.74	0.58	2.91	7.56	4.65	4.07		
	SIDER	SIDER	SIDER							3.00	17.00
**Convulsion**	SLAM	Trust	Clozapine (n=1760)	1.36	1.70	1.82	7.05	4.94	4.03		
		Gender	Male (n=1167)	1.37	1.89	1.63	6.86	4.54	4.03		
			Female (n=593)	1.35	1.35	2.19	7.42	5.73	4.05		
	Camden & Islington	Trust	Clozapine (n=561)	0.53	0.53	0.36	2.85	2.14	1.07		
		Gender	Male (n=357)	0.56	0.56	0.56	3.08	2.24	0.84		
			Female (n=204)	0.49	0.49	0.00	2.45	1.96	1.47		
	Oxford	Trust	Clozapine (n=514)	1.36	1.36	1.56	6.42	3.11	2.72		
		Gender	Male (n=342)	0.88	0.88	1.17	6.43	4.09	2.05		

The results are shown in percentages (%) and broken down by ADRs, Trusts (SLAM, Camden & Islington and Oxford), Cohorts, Sub Cohorts and SIDER reported values.
In Sub Cohort 'Clozapine' represent the total baseline population which further breaks down into Gender 'Male' and 'Female' groups.
The columns (Three Months Early, Two Months Early, One Month Early, One Month Later, Two Months Later, Three Months Later) shows the percentages in each monthly interval. The last two columns (SIDER Low End and SIDER High End) shows the SIDER reporting.

Clozapine - Gender Differences (%)

Measure Values: 0.00 – 56.00

ADR	Trust	Cohort	Sub Cohort	Three Months Early	Two Months Early	One Month Early	One Month Later	Two Months Later	Three Months Later	SIDER Low End	SIDER High End
Convulsion	Oxford	Gender	Female (n=172)	2.33	2.33	2.33	6.40	1.16	4.07		
	SIDER	SIDER	SIDER							3.00	
Hypotension	SLAM	Trust	Clozapine (n=1760)	0.51	0.97	0.80	5.00	2.95	2.56		
		Gender	Male (n=1167)	0.43	0.60	0.34	3.86	2.83	2.49		
			Female (n=593)	0.67	1.69	1.69	7.25	3.20	2.70		
	Camden & Islington	Trust	Clozapine (n=561)	0.18	0.53	0.18	3.57	2.32	1.78		
		Gender	Male (n=357)	0.28	0.56	0.28	2.52	1.96	1.12		
			Female (n=204)	0.00	0.49	0.00	5.39	2.94	2.94		
	Oxford	Trust	Clozapine (n=514)	0.58	0.78	0.78	5.64	3.50	2.92		
		Gender	Male (n=342)	0.00	0.29	0.29	4.09	2.34	1.75		
			Female (n=172)	1.74	1.74	1.74	8.72	5.81	5.23		
	SIDER	SIDER	SIDER							9.00	38.00
Enuresis	SLAM	Trust	Clozapine (n=1760)	1.02	0.80	1.25	4.20	3.92	3.24		
		Gender	Male (n=1167)	0.60	0.51	0.86	3.17	2.74	2.74		
			Female (n=593)	1.85	1.35	2.02	6.24	6.24	4.22		
	Camden & Islington	Trust	Clozapine (n=561)	0.71	1.07	1.07	4.10	1.43	1.25		
		Gender	Male (n=357)	0.28	0.84	0.56	3.36	0.84	1.12		
			Female (n=204)	1.47	1.47	1.96	5.39	2.45	1.47		
	Oxford	Trust	Clozapine (n=514)	1.36	0.58	1.36	4.86	4.47	3.50		
		Gender	Male (n=342)	0.88	0.00	1.17	4.39	3.22	2.05		
			Female (n=172)	2.33	1.74	1.74	5.81	6.98	6.40		
	SIDER	SIDER	SIDER								
Fever	SLAM	Trust	Clozapine (n=1760)	1.02	1.14	1.65	6.36	4.43	3.13		
		Gender	Male (n=1167)	0.77	0.86	1.20	5.14	3.68	3.00		
			Female (n=593)	1.52	1.69	2.53	8.77	5.90	3.37		
	Camden & Islington	Trust	Clozapine (n=561)	0.89	0.89	0.53	3.74	2.67	0.89		
		Gender	Male (n=357)	0.56	0.84	0.56	3.64	1.68	0.84		
			Female (n=204)	1.47	0.98	0.49	3.92	4.41	0.98		
	Oxford	Trust	Clozapine (n=514)	0.39	0.78	0.58	3.11	2.72	2.33		
		Gender	Male (n=342)	0.58	0.88	0.58	3.22	2.05	2.05		
			Female (n=172)	0.00	0.58	0.58	2.91	4.07	2.91		
	SIDER	SIDER	SIDER							4.00	13.00
Diarrhoea	SLAM	Trust	Clozapine (n=1760)	1.08	1.31	1.36	4.72	3.58	2.56		
		Gender	Male (n=1167)	0.86	1.37	0.69	3.86	3.43	1.80		
			Female (n=593)	1.52	1.18	2.70	6.41	3.88	4.05		
	Camden & Islington	Trust	Clozapine (n=561)	0.71	1.25	0.18	3.03	3.39	3.03		
		Gender	Male (n=357)	0.28	0.84	0.28	2.80	3.08	2.24		
			Female (n=204)	1.47	1.96	0.00	3.43	3.92	4.41		
	Oxford	Trust	Clozapine (n=514)	1.17	0.78	1.36	4.09	3.70	2.53		
		Gender	Male (n=342)	1.17	0.29	1.17	2.34	2.63	1.17		
			Female (n=172)	1.16	1.74	1.74	7.56	5.81	5.23		
	SIDER	SIDER	SIDER							2.00	
Drymouth	SLAM	Trust	Clozapine (n=1760)	1.08	1.53	1.65	4.66	3.69	2.33		
		Gender	Male (n=1167)	0.86	1.20	1.46	4.20	3.00	2.23		
			Female (n=593)	1.52	2.19	2.02	5.56	5.06	2.53		
	Camden & Islington	Trust	Clozapine (n=561)	1.25	1.25	1.07	3.92	2.14	0.89		
		Gender	Male (n=357)	0.84	1.40	1.40	4.48	1.68	0.28		
			Female (n=204)	1.96	0.98	0.49	2.94	2.94	1.96		
	Oxford	Trust	Clozapine (n=514)	1.36	1.36	1.56	3.89	1.36	2.33		
		Gender	Male (n=342)	1.17	0.88	1.46	4.39	1.46	1.46		
			Female (n=172)	1.74	2.91	1.74	2.91	1.16	4.07		
	SIDER	SIDER	SIDER							5.00	20.00
Rash	SLAM	Trust	Clozapine (n=1760)	1.25	1.59	2.05	3.64	2.95	2.27		
		Gender	Male (n=1167)	0.86	1.29	1.46	3.17	2.57	1.89		
			Female (n=593)	2.02	2.19	3.20	4.55	3.71	3.04		
	Camden & Islington	Trust	Clozapine (n=561)	1.25	1.25	0.89	4.28	1.96	2.14		
		Gender	Male (n=357)	0.56	1.12	1.12	4.76	1.68	2.24		
			Female (n=204)	2.45	1.47	0.49	3.43	2.45	1.96		
	Oxford	Trust	Clozapine (n=514)	0.97	1.17	1.17	3.70	2.33	1.36		
		Gender	Male (n=342)	1.17	0.88	1.17	2.92	1.75	0.88		
			Female (n=172)	0.58	1.74	1.16	5.23	3.49	2.33		
	SIDER	SIDER	SIDER								
Dyspepsia	SLAM	Trust	Clozapine (n=1760)	0.74	1.08	0.91	3.92	3.13	3.69		
		Gender	Male (n=1167)	0.51	0.77	0.60	3.86	2.74	3.43		
			Female (n=593)	1.18	1.69	1.52	4.05	3.88	4.22		
	Camden &	Trust	Clozapine (n=561)	0.36	0.53	0.53	4.10	2.67	2.50		

The results are shown in percentages (%) and broken down by ADRs, Trusts (SLAM, Camden & Islington and Oxford), Cohorts, Sub Cohorts and SIDER reported values.
In Sub Cohort 'Clozapine' represent the total baseline population which further breaks down into Gender 'Male' and 'Female' groups.
The columns (Three Months Early, Two Months Early, One Month Early, One Month Later, Two Months Later, Three Months Later) shows the percentages in each monthly interval. The last two columns (SIDER Low End and SIDER High End) shows the SIDER reporting.

Clozapine - Gender Differences (%)

Measure Values
0.00 56.00

ADR	Trust	Cohort	Sub Cohort	Three Months Early	Two Months Early	One Month Early	One Month Later	Two Months Later	Three Months Later	SIDER Low End	SIDER High End
**Dyspepsia**	Camden & Islington	Gender	Male (n=357)	0.00	0.84	0.56	5.04	3.08	3.36		
			Female (n=204)	0.98	0.00	0.49	2.45	1.96	0.98		
	Oxford	Trust	Clozapine (n=514)	0.19	0.58	0.78	3.50	4.09	3.70		
		Gender	Male (n=342)	0.29	0.58	0.58	2.34	3.22	2.92		
			Female (n=172)	0.00	0.58	1.16	5.81	5.81	5.23		
	SIDER	SIDER	SIDER							8.00	14.00
**Stomachpain**	SLAM	Trust	Clozapine (n=1760)	1.93	1.76	1.93	4.94	3.52	3.52		
		Gender	Male (n=1167)	1.03	1.37	1.20	3.51	2.91	2.14		
			Female (n=593)	3.71	2.53	3.37	7.76	4.72	6.24		
	Camden & Islington	Trust	Clozapine (n=561)	0.89	1.25	0.89	3.39	2.85	2.14		
		Gender	Male (n=357)	0.84	0.84	0.56	2.80	1.96	1.68		
			Female (n=204)	0.98	1.96	1.47	4.41	4.41	2.94		
	Oxford	Trust	Clozapine (n=514)	1.56	0.78	0.78	2.14	0.97	0.97		
		Gender	Male (n=342)	1.46	0.88	0.29	2.34	0.88	0.58		
			Female (n=172)	1.74	0.58	1.74	1.74	1.16	1.74		
	SIDER	SIDER	SIDER								
**Sweating**	SLAM	Trust	Clozapine (n=1760)	1.08	0.97	1.36	4.43	4.26	2.84		
		Gender	Male (n=1167)	1.20	1.11	1.63	4.37	4.97	3.00		
			Female (n=593)	0.84	0.67	0.84	4.55	2.87	2.53		
	Camden & Islington	Trust	Clozapine (n=561)	0.53	0.53	0.53	2.85	2.14	1.96		
		Gender	Male (n=357)	0.28	0.28	0.56	3.64	2.52	1.40		
			Female (n=204)	0.98	0.98	0.49	1.47	1.47	2.94		
	Oxford	Trust	Clozapine (n=514)	1.17	0.97	0.97	2.72	1.36	1.95		
		Gender	Male (n=342)	1.75	1.17	0.88	2.92	1.17	1.46		
			Female (n=172)	0.00	0.58	1.16	2.33	1.74	2.91		
	SIDER	SIDER	SIDER							6.00	
**Tremor**	SLAM	Trust	Clozapine (n=1760)	1.48	1.99	2.95	5.51	3.52	3.47		
		Gender	Male (n=1167)	1.03	2.14	3.26	5.74	3.43	3.51		
			Female (n=593)	2.36	1.69	2.36	5.06	3.71	3.37		
	Camden & Islington	Trust	Clozapine (n=561)	1.60	1.78	2.14	3.92	1.96	2.14		
		Gender	Male (n=357)	1.68	2.24	2.52	3.92	1.96	2.52		
			Female (n=204)	1.47	0.98	1.47	3.92	1.96	1.47		
	SIDER	SIDER	SIDER							6.00	
**Neutropenia**	SLAM	Trust	Clozapine (n=1760)	0.80	0.80	0.74	5.34	2.73	2.61		
		Gender	Male (n=1167)	0.94	0.51	0.69	5.91	3.00	2.49		
			Female (n=593)	0.51	1.35	0.84	4.22	2.19	2.87		
	Camden & Islington	Trust	Clozapine (n=561)	0.00	0.18	0.53	1.60	0.89	1.07		
		Gender	Male (n=357)	0.00	0.00	0.00	1.12	0.84	0.84		
			Female (n=204)	0.00	0.49	1.47	2.45	0.98	1.47		
	SIDER	SIDER	SIDER								
**Akathisia**	SLAM	Trust	Clozapine (n=1760)	0.80	0.91	0.74	2.67	1.36	0.80		
		Gender	Male (n=1167)	0.77	0.86	0.69	2.74	1.03	0.77		
			Female (n=593)	0.84	1.01	0.84	2.53	2.02	0.84		
	Camden & Islington	Trust	Clozapine (n=561)	0.00	0.53	0.00	1.25	1.07	0.53		
		Gender	Male (n=357)	0.00	0.56	0.00	1.40	1.12	0.56		
			Female (n=204)	0.00	0.49	0.00	0.98	0.98	0.49		
	Oxford	Trust	Clozapine (n=514)	0.97	0.78	0.97	1.36	1.17	0.97		
		Gender	Male (n=342)	1.46	0.58	0.88	1.17	1.17	0.88		
			Female (n=172)	0.00	1.16	1.16	1.74	1.16	1.16		
	SIDER	SIDER	SIDER							3.00	
**Blurredvision**	SLAM	Trust	Clozapine (n=1760)	0.34	0.91	0.63	2.05	1.25	1.02		
		Gender	Male (n=1167)	0.43	0.94	0.51	1.37	1.20	0.94		
			Female (n=593)	0.17	0.84	0.84	3.37	1.35	1.18		
	Camden & Islington	Trust	Clozapine (n=561)	0.89	0.53	0.71	1.25	0.36	0.89		
		Gender	Male (n=357)	0.84	0.28	0.56	1.12	0.28	0.84		
			Female (n=204)	0.98	0.98	0.98	1.47	0.49	0.98		
	Oxford	Trust	Clozapine (n=514)	0.19	0.39	0.39	1.56	1.56	1.17		
		Gender	Male (n=342)	0.29	0.29	0.29	1.46	1.75	0.88		
			Female (n=172)	0.00	0.58	0.58	1.74	1.16	1.74		
	SIDER	SIDER	SIDER							5.00	

The results are shown in percentages (%) and broken down by ADRs, Trusts (SLAM, Camden & Islington and Oxford), Cohorts, Sub Cohorts and SIDER reported values.
In Sub Cohort 'Clozapine' represent the total baseline population which further breaks down into Gender 'Male' and 'Female' groups.
The columns (Three Months Early, Two Months Early, One Month Early, One Month Later, Two Months Later, Three Months Later) shows the percentages in each monthly interval. The last two columns (SIDER Low End and SIDER High End) shows the SIDER reporting.

Clozapine - Ethnic Background (%)

ADR	Trust	Cohort	Sub Cohort	Three Months Early	Two Months Early	One Month Early	One Month Later	Two Months Later	Three Months Later	SIDER Low End	SIDER High End
Agitation	SLAM	Trust	Clozapine (n=1760)	17.61	22.10	26.53	46.59	32.56	26.99		
		Ethnic Background	White (n=821)	16.32	21.80	23.26	38.98	26.31	22.53		
			Black (n=704)	19.03	25.43	27.13	45.45	30.68	26.28		
			Asian (n=93)	18.28	19.35	24.73	45.16	25.81	23.66		
			Other (n=142)	22.54	21.13	30.99	45.77	37.32	30.28		
	Camden & Islington	Trust	Clozapine (n=561)	13.37	17.83	18.36	43.14	28.34	21.03		
		Ethnic Background	White (n=347)	12.10	16.43	18.44	40.63	26.51	19.02		
			Black (n=120)	15.83	25.83	22.50	49.17	32.50	21.67		
			Asian (n=41)	17.07	9.76	14.63	51.22	29.27	31.71		
			Others (n=53)	13.21	11.32	11.32	28.30	22.64	22.64		
	Oxford	Trust	Clozapine (n=514)	14.59	15.76	16.34	34.24	25.10	20.62		
		Ethnic Background	White (n=426)	14.55	15.73	16.20	34.74	25.82	21.36		
			Black (n=20)	10.00	10.00	10.00	35.00	25.00	15.00		
			Asian (n=41)	12.20	17.07	19.51	36.59	21.95	19.51		
			Others (n=27)	22.22	18.52	18.52	22.22	18.52	14.81		
	SIDER	SIDER	SIDER							4.00	
Fatigue	SLAM	Trust	Clozapine (n=1760)	12.67	14.83	15.85	43.58	35.80	30.51		
		Ethnic Background	White (n=821)	13.03	13.89	14.62	35.69	29.72	24.24		
			Black (n=704)	15.20	16.19	17.05	41.62	34.66	28.27		
			Asian (n=93)	15.05	11.83	16.13	45.16	30.11	24.73		
			Other (n=142)	13.38	12.68	12.68	43.66	34.51	31.69		
	Camden & Islington	Trust	Clozapine (n=561)	10.34	12.30	13.37	41.18	29.23	26.56		
		Ethnic Background	White (n=347)	8.65	13.26	11.24	39.77	30.26	24.21		
			Black (n=120)	15.00	11.67	18.33	41.67	30.00	32.50		
			Asian (n=41)	12.20	7.32	14.63	51.22	29.27	36.59		
			Others (n=53)	9.43	5.66	11.32	30.19	24.53	20.75		
	Oxford	Trust	Clozapine (n=514)	9.73	11.87	12.06	35.21	27.43	26.85		
		Ethnic Background	White (n=426)	9.62	12.21	12.44	35.45	26.53	26.53		
			Black (n=20)	5.00	10.00	10.00	30.00	25.00	30.00		
			Asian (n=41)	4.88	4.88	4.88	29.27	24.39	24.39		
			Others (n=27)	22.22	18.52	18.52	44.44	48.15	33.33		
	SIDER	SIDER	SIDER								
Sedation	SLAM	Trust	Clozapine (n=1760)	12.67	12.16	14.83	43.86	35.51	29.83		
		Ethnic Background	White (n=821)	12.91	11.57	14.25	38.49	30.33	23.63		
			Black (n=704)	15.06	13.49	16.62	44.89	35.37	27.56		
			Asian (n=93)	17.20	12.90	11.83	46.24	35.48	23.66		
			Other (n=142)	16.20	14.08	14.79	42.25	38.73	28.87		
	Camden & Islington	Trust	Clozapine (n=561)	5.17	9.09	9.09	38.15	26.56	21.93		
		Ethnic Background	White (n=347)	3.17	9.22	7.20	37.18	25.65	21.04		
			Black (n=120)	10.83	11.67	15.83	39.17	29.17	25.83		
			Asian (n=41)	7.32	4.88	9.76	46.34	31.71	24.39		
			Others (n=53)	5.66	5.66	7.55	24.53	18.87	16.98		
	Oxford	Trust	Clozapine (n=514)	7.20	8.37	9.34	31.52	21.40	18.48		
		Ethnic Background	White (n=426)	7.04	7.75	8.45	30.75	20.66	18.54		
			Black (n=20)	10.00	5.00	15.00	30.00	30.00	30.00		
			Asian (n=41)	2.44	12.20	12.20	31.71	14.63	14.63		
			Others (n=27)	14.81	14.81	14.81	44.44	37.04	14.81		
	SIDER	SIDER	SIDER							25.00	46.00
Dizziness	SLAM	Trust	Clozapine (n=1760)	2.78	4.20	4.09	16.59	13.13	11.19		
		Ethnic Background	White (n=821)	3.29	4.51	3.53	12.67	8.40	6.94		
			Black (n=704)	3.84	5.26	4.12	14.77	9.80	8.10		
			Asian (n=93)	4.30	1.08	3.23	15.05	17.20	7.53		
			Other (n=142)	0.70	3.52	2.11	18.31	14.79	17.61		
	Camden & Islington	Trust	Clozapine (n=561)	3.21	3.39	3.74	18.18	13.73	9.09		
		Ethnic Background	White (n=347)	4.03	3.46	2.88	18.73	14.41	9.22		
			Black (n=120)	2.50	2.50	5.83	19.17	13.33	10.00		
			Asian (n=41)	0.00	7.32	4.88	19.51	17.07	12.20		
			Others (n=53)	3.77	3.77	5.66	11.32	5.66	3.77		
	Oxford	Trust	Clozapine (n=514)	3.89	4.09	4.47	17.70	13.04	10.12		
		Ethnic Background	White (n=426)	3.76	3.99	4.46	18.31	12.91	10.33		
			Black (n=20)	5.00	0.00	5.00	15.00	5.00	10.00		
			Asian (n=41)	2.44	7.32	4.88	9.76	12.20	9.76		
			Others (n=27)	7.41	3.70	3.70	22.22	22.22	7.41		
	SIDER	SIDER	SIDER							12.00	27.00
Tachycardia	SLAM	Trust	Clozapine (n=1760)	2.27	2.05	2.50	15.40	12.95	9.94		
		Ethnic Background	White (n=821)	1.95	2.68	2.44	13.03	11.21	7.80		
			Black (n=704)	2.27	3.13	2.84	15.20	13.07	9.09		

Measure Values: 0.00 – 56.00

The results are shown in percentages (%) and broken down by ADRs, Trusts (SLAM, Camden & Islington and Oxford), Cohorts, Sub Cohorts and SIDER reported values.
In Sub Cohort 'Clozapine' represent the total baseline population which further breaks down into 'White', 'Black', 'Asian' and 'Others' ethnic groups.
The columns (Three Months Early, Two Months Early, One Month Early, One Month Later, Two Months Later, Three Months Later) shows the percentages in each monthly interval. The last two columns (SIDER Low End and SIDER High End) shows the SIDER reporting.

Clozapine - Ethnic Background (%)

Measure Values: 0.00 – 56.00

ADR	Trust	Cohort	Sub Cohort	Three Months Early	Two Months Early	One Month Early	One Month Later	Two Months Later	Three Months Later	SIDER Low End	SIDER High End
**Tachycardia**	SLAM	Ethnic Background	Asian (n=93)	0.00	2.15	3.23	18.28	12.90	5.38		
			Other (n=142)	2.11	2.11	1.41	16.20	16.90	12.68		
	Camden & Islington	Trust	Clozapine (n=561)	1.43	1.43	0.89	11.23	8.38	6.95		
		Ethnic Background	White (n=347)	1.44	0.29	1.15	9.22	8.36	5.19		
			Black (n=120)	1.67	4.17	0.83	15.00	8.33	10.00		
			Asian (n=41)	0.00	2.44	0.00	24.39	9.76	7.32		
			Others (n=53)	3.77	0.00	0.00	7.55	5.66	13.21		
	Oxford	Trust	Clozapine (n=514)	0.78	1.36	1.56	10.89	10.51	7.59		
		Ethnic Background	White (n=426)	0.70	1.41	1.64	10.80	9.39	7.28		
			Black (n=20)	0.00	0.00	0.00	20.00	20.00	5.00		
			Asian (n=41)	2.44	2.44	2.44	9.76	9.76	4.88		
			Others (n=27)	0.00	0.00	0.00	7.41	22.22	18.52		
	SIDER	SIDER	SIDER							11.00	25.00
**Weightgain**	SLAM	Trust	Clozapine (n=1760)	3.75	4.43	5.06	15.34	10.91	10.34		
		Ethnic Background	White (n=821)	4.14	4.26	4.99	13.40	9.87	9.14		
			Black (n=704)	4.83	4.97	5.82	15.63	11.51	10.65		
			Asian (n=93)	1.08	5.38	6.45	19.35	9.68	9.68		
			Other (n=142)	4.93	6.34	4.23	19.01	14.08	12.68		
	Camden & Islington	Trust	Clozapine (n=561)	2.50	3.39	1.96	11.76	6.60	6.24		
		Ethnic Background	White (n=347)	2.88	3.75	2.02	13.26	6.63	5.48		
			Black (n=120)	1.67	2.50	3.33	10.83	7.50	9.17		
			Asian (n=41)	4.88	4.88	0.00	9.76	7.32	9.76		
			Others (n=53)	0.00	3.77	0.00	9.43	7.55	7.55		
	Oxford	Trust	Clozapine (n=514)	3.50	3.31	3.70	11.28	9.92	7.78		
		Ethnic Background	White (n=426)	3.99	3.29	3.52	11.03	10.09	8.45		
			Black (n=20)	0.00	0.00	0.00	20.00	5.00	5.00		
			Asian (n=41)	0.00	0.00	0.00	7.32	4.88	0.00		
			Others (n=27)	3.70	11.11	14.81	14.81	18.52	11.11		
	SIDER	SIDER	SIDER							4.00	56.00
**Hypersalivation**	SLAM	Trust	Clozapine (n=1760)	1.19	1.48	2.10	14.32	13.24	11.31		
		Ethnic Background	White (n=821)	1.10	1.34	1.34	12.79	11.57	9.99		
			Black (n=704)	1.28	1.56	1.56	14.91	13.49	11.65		
			Asian (n=93)	0.00	2.15	4.30	15.05	12.90	8.60		
			Other (n=142)	2.11	1.41	4.23	16.20	16.20	11.97		
	Camden & Islington	Trust	Clozapine (n=561)	1.07	1.43	0.53	14.26	6.95	7.66		
		Ethnic Background	White (n=347)	0.58	1.44	0.58	15.56	7.49	8.65		
			Black (n=120)	0.83	2.50	0.00	10.83	6.67	7.50		
			Asian (n=41)	0.00	0.00	2.44	17.07	7.32	0.00		
			Others (n=53)	5.66	0.00	0.00	7.55	1.89	7.55		
	Oxford	Trust	Clozapine (n=514)	0.97	0.78	1.56	12.65	10.70	5.84		
		Ethnic Background	White (n=426)	0.23	0.94	1.64	13.38	11.27	6.10		
			Black (n=20)	10.00	0.00	0.00	10.00	5.00	0.00		
			Asian (n=41)	2.44	0.00	0.00	7.32	9.76	7.32		
			Others (n=27)	3.70	0.00	3.70	11.11	7.41	3.70		
	SIDER	SIDER	SIDER							1.00	48.00
**Feelingsick**	SLAM	Trust	Clozapine (n=1760)	4.66	4.94	6.48	14.32	11.19	9.09		
		Ethnic Background	White (n=821)	4.51	4.38	5.72	12.67	9.38	6.46		
			Black (n=704)	5.26	5.11	6.68	14.77	10.94	7.53		
			Asian (n=93)	8.60	5.38	5.38	10.75	8.60	6.45		
			Other (n=142)	5.63	7.04	8.45	14.79	12.68	11.27		
	Camden & Islington	Trust	Clozapine (n=561)	3.74	3.92	3.03	10.52	7.13	7.66		
		Ethnic Background	White (n=347)	3.17	3.75	3.75	11.53	8.65	7.20		
			Black (n=120)	5.00	6.67	2.50	10.00	3.33	10.00		
			Asian (n=41)	2.44	0.00	2.44	4.88	2.44	2.44		
			Others (n=53)	5.66	3.77	1.89	13.21	5.66	7.55		
	Oxford	Trust	Clozapine (n=514)	3.89	5.25	5.06	14.20	9.73	7.20		
		Ethnic Background	White (n=426)	3.76	4.93	5.16	14.79	9.39	7.75		
			Black (n=20)	0.00	0.00	0.00	10.00	5.00	0.00		
			Asian (n=41)	4.88	4.88	7.32	12.20	7.32	4.88		
			Others (n=27)	7.41	14.81	3.70	11.11	22.22	7.41		
	SIDER	SIDER	SIDER								
**Confusion**	SLAM	Trust	Clozapine (n=1760)	4.72	5.51	6.08	13.92	8.47	6.76		
		Ethnic Background	White (n=821)	5.36	5.85	5.85	12.55	6.58	5.48		
			Black (n=704)	6.25	6.82	6.82	14.63	7.67	6.39		
			Asian (n=93)	2.15	0.00	4.30	10.75	10.75	3.23		
			Other (n=142)	7.04	3.52	4.93	11.27	9.86	7.75		
	Camden &	Trust	Clozapine (n=561)	3.57	6.24	5.53	12.66	6.77	5.88		

The results are shown in percentages (%) and broken down by ADRs, Trusts (SLAM, Camden & Islington and Oxford), Cohorts, Sub Cohorts and SIDER reported values.
In Sub Cohort 'Clozapine' represent the total baseline population which further breaks down into 'White', 'Black', 'Asian' and 'Others' ethnic groups. The columns (Three Months Early, Two Months Early, One Month Early, One Month Later, Two Months Later, Three Months Later) shows the percentages in each monthly interval. The last two columns (SIDER Low End and SIDER High End) shows the SIDER reporting.

Clozapine - Ethnic Background (%)

Measure Values: 0.00 – 56.00

ADR	Trust	Cohort	Sub Cohort	Three Months Early	Two Months Early	One Month Early	One Month Later	Two Months Later	Three Months Later	SIDER Low End	SIDER High End
Confusion	Camden & Islington	Ethnic Background	White (n=347)	3.75	5.19	6.05	10.95	6.34	4.90		
			Black (n=120)	4.17	8.33	7.50	19.17	11.67	9.17		
			Asian (n=41)	2.44	9.76	0.00	14.63	2.44	4.88		
			Others (n=53)	1.89	5.66	3.77	9.43	1.89	5.66		
	Oxford	Trust	Clozapine (n=514)	2.53	3.89	3.89	9.92	6.42	5.25		
		Ethnic Background	White (n=426)	2.35	3.99	4.23	10.80	5.87	5.16		
			Black (n=20)	0.00	0.00	0.00	10.00	10.00	10.00		
			Asian (n=41)	0.00	2.44	0.00	0.00	9.76	4.88		
			Others (n=27)	11.11	7.41	7.41	11.11	7.41	3.70		
	SIDER	SIDER	SIDER							3.00	
Constipation	SLAM	Trust	Clozapine (n=1760)	1.76	1.99	2.16	12.27	11.70	9.49		
		Ethnic Background	White (n=821)	1.46	1.83	2.31	9.14	9.14	8.04		
			Black (n=704)	1.70	2.13	2.70	10.65	10.65	9.38		
			Asian (n=93)	1.08	1.08	0.00	20.43	17.20	8.60		
			Other (n=142)	4.93	2.82	2.11	14.79	14.79	9.86		
	Camden & Islington	Trust	Clozapine (n=561)	1.07	2.50	1.78	11.41	7.13	5.70		
		Ethnic Background	White (n=347)	1.44	2.59	2.31	12.97	8.07	6.05		
			Black (n=120)	0.00	3.33	0.83	9.17	8.33	6.67		
			Asian (n=41)	0.00	0.00	0.00	14.63	2.44	2.44		
			Others (n=53)	1.89	1.89	1.89	3.77	3.77	3.77		
	Oxford	Trust	Clozapine (n=514)	0.58	0.97	1.36	10.31	7.78	7.78		
		Ethnic Background	White (n=426)	0.70	0.94	1.17	10.09	8.22	7.51		
			Black (n=20)	0.00	0.00	5.00	5.00	5.00	15.00		
			Asian (n=41)	0.00	2.44	2.44	12.20	7.32	9.76		
			Others (n=27)	0.00	0.00	0.00	14.81	3.70	3.70		
	SIDER	SIDER	SIDER							10.00	25.00
Headache	SLAM	Trust	Clozapine (n=1760)	4.20	4.55	5.45	12.44	8.18	5.91		
		Ethnic Background	White (n=821)	4.38	5.24	5.36	10.35	7.80	4.51		
			Black (n=704)	5.11	6.11	6.25	12.07	9.09	5.26		
			Asian (n=93)	2.15	3.23	4.30	13.98	6.45	7.53		
			Other (n=142)	5.63	8.45	5.63	14.08	10.56	7.04		
	Camden & Islington	Trust	Clozapine (n=561)	2.32	3.57	4.28	9.27	6.42	4.63		
		Ethnic Background	White (n=347)	2.31	3.17	3.46	8.65	5.76	4.03		
			Black (n=120)	2.50	5.00	6.67	10.00	6.67	6.67		
			Asian (n=41)	4.88	4.88	2.44	12.20	12.20	4.88		
			Others (n=53)	0.00	3.77	5.66	5.66	1.89	1.89		
	Oxford	Trust	Clozapine (n=514)	3.89	3.89	4.09	10.89	8.37	7.59		
		Ethnic Background	White (n=426)	3.99	3.76	3.99	10.33	8.92	7.75		
			Black (n=20)	5.00	5.00	5.00	5.00	10.00	0.00		
			Asian (n=41)	4.88	7.32	7.32	19.51	4.88	14.63		
			Others (n=27)	0.00	0.00	0.00	11.11	3.70	0.00		
	SIDER	SIDER	SIDER								
Hyperprolactinaemia	SLAM	Trust	Clozapine (n=1760)	3.18	3.64	4.20	8.52	5.06	4.15		
		Ethnic Background	White (n=821)	3.41	4.14	5.12	8.40	5.12	4.02		
			Black (n=704)	3.98	4.83	5.97	9.80	5.97	4.69		
			Asian (n=93)	4.30	3.23	4.30	12.90	3.23	2.15		
			Other (n=142)	3.52	4.93	3.52	8.45	4.23	3.52		
	Camden & Islington	Trust	Clozapine (n=561)	1.60	1.78	2.67	8.20	4.10	3.57		
		Ethnic Background	White (n=347)	0.29	1.44	1.73	6.05	3.46	3.46		
			Black (n=120)	5.00	4.17	5.00	14.17	4.17	2.50		
			Asian (n=41)	2.44	0.00	4.88	12.20	2.44	2.44		
			Others (n=53)	1.89	0.00	1.89	5.66	5.66	5.66		
	Oxford	Trust	Clozapine (n=514)	3.70	4.09	4.28	8.75	4.47	4.86		
		Ethnic Background	White (n=426)	3.52	3.76	3.99	8.45	4.46	3.99		
			Black (n=20)	5.00	0.00	5.00	10.00	5.00	5.00		
			Asian (n=41)	0.00	2.44	2.44	7.32	2.44	7.32		
			Others (n=27)	11.11	14.81	11.11	14.81	7.41	14.81		
	SIDER	SIDER	SIDER								
Insomnia	SLAM	Trust	Clozapine (n=1760)	3.92	4.03	5.17	10.40	6.48	4.03		
		Ethnic Background	White (n=821)	3.29	3.90	5.36	7.80	5.24	3.05		
			Black (n=704)	3.84	4.55	6.25	9.09	6.11	3.55		
			Asian (n=93)	7.53	3.23	9.68	11.83	3.23	4.30		
			Other (n=142)	7.75	4.93	5.63	10.56	7.75	4.93		
	Camden & Islington	Trust	Clozapine (n=561)	3.57	3.39	3.74	8.91	3.39	4.28		
		Ethnic Background	White (n=347)	3.75	3.46	4.03	8.93	3.75	4.03		
			Black (n=120)	4.17	4.17	2.50	8.33	2.50	5.00		
			Asian (n=41)	2.44	2.44	4.88	12.20	4.88	4.88		

The results are shown in percentages (%) and broken down by ADRs, Trusts (SLAM, Camden & Islington and Oxford), Cohorts, Sub Cohorts and SIDER reported values.
In Sub Cohort 'Clozapine' represent the total baseline population which further breaks down into 'White', 'Black', 'Asian' and 'Others' ethnic groups.
The columns (Three Months Early, Two Months Early, One Month Early, One Month Later, Two Months Later, Three Months Later) shows the percentages in each monthly interval. The last two columns (SIDER Low End and SIDER High End) shows the SIDER reporting.

Clozapine - Ethnic Background (%)

Measure Values: 0.00 – 56.00

ADR	Trust	Cohort	Sub Cohort	Three Months Early	Two Months Early	One Month Early	One Month Later	Two Months Later	Three Months Later	SIDER Low End	SIDER High End
Insomnia	Islington	Background	Others (n=53)	1.89	0.00	3.77	9.43	3.77	3.77		
	Oxford	Trust	Clozapine (n=514)	5.84	4.86	5.84	8.37	6.81	4.09		
		Ethnic	White (n=426)	5.87	4.69	5.63	8.92	6.57	4.69		
		Background	Black (n=20)	10.00	0.00	0.00	5.00	5.00	5.00		
			Asian (n=41)	4.88	7.32	7.32	7.32	12.20	0.00		
			Others (n=27)	3.70	7.41	11.11	3.70	3.70	0.00		
	SIDER	SIDER	SIDER							20.00	33.00
Hypertension	SLAM	Trust	Clozapine (n=1760)	2.05	2.22	3.13	9.15	5.74	4.60		
		Ethnic	White (n=821)	2.68	3.17	4.02	10.48	7.19	5.60		
		Background	Black (n=704)	3.13	3.69	4.69	12.22	8.38	6.53		
			Asian (n=93)	1.08	2.15	2.15	8.60	5.38	2.15		
			Other (n=142)	1.41	1.41	2.11	11.27	4.93	4.23		
	Camden & Islington	Trust	Clozapine (n=561)	0.71	0.71	1.60	7.13	4.63	2.67		
		Ethnic	White (n=347)	0.58	0.86	2.02	8.07	5.48	2.02		
		Background	Black (n=120)	0.83	0.83	0.83	7.50	3.33	5.00		
			Asian (n=41)	0.00	0.00	0.00	2.44	2.44	2.44		
			Others (n=53)	1.89	0.00	1.89	3.77	1.89	1.89		
	Oxford	Trust	Clozapine (n=514)	1.36	1.36	1.56	5.06	4.28	2.14		
		Ethnic	White (n=426)	1.17	1.41	1.41	3.99	3.99	1.88		
		Background	Black (n=20)	0.00	0.00	0.00	0.00	10.00	0.00		
			Asian (n=41)	0.00	0.00	0.00	7.32	2.44	2.44		
			Others (n=27)	7.41	3.70	7.41	22.22	7.41	7.41		
	SIDER	SIDER	SIDER							4.00	12.00
Vomiting	SLAM	Trust	Clozapine (n=1760)	2.56	2.50	3.01	8.86	6.82	5.00		
		Ethnic	White (n=821)	2.68	1.95	2.92	7.80	5.60	3.53		
		Background	Black (n=704)	3.13	2.27	3.41	9.09	6.53	4.12		
			Asian (n=93)	4.30	3.23	3.23	5.38	8.60	5.38		
			Other (n=142)	1.41	4.93	4.23	6.34	7.75	4.93		
	Camden & Islington	Trust	Clozapine (n=561)	2.14	2.50	2.85	6.77	4.99	4.63		
		Ethnic	White (n=347)	1.73	2.88	2.59	6.63	4.03	3.75		
		Background	Black (n=120)	2.50	2.50	4.17	10.00	8.33	7.50		
			Asian (n=41)	2.44	2.44	2.44	2.44	2.44	0.00		
			Others (n=53)	3.77	0.00	1.89	3.77	5.66	5.66		
	Oxford	Trust	Clozapine (n=514)	1.75	2.72	2.92	7.59	5.25	5.06		
		Ethnic	White (n=426)	1.88	2.35	2.58	7.51	5.16	5.87		
		Background	Black (n=20)	0.00	5.00	5.00	5.00	5.00	0.00		
			Asian (n=41)	2.44	4.88	2.44	7.32	7.32	2.44		
			Others (n=27)	0.00	3.70	7.41	11.11	3.70	0.00		
	SIDER	SIDER	SIDER							3.00	17.00
Shaking	SLAM	Trust	Clozapine (n=1760)	3.13	2.95	3.92	9.55	5.40	5.06		
		Ethnic	White (n=821)	3.17	2.80	3.29	7.67	4.26	3.41		
		Background	Black (n=704)	3.69	3.27	3.84	8.95	4.97	3.98		
			Asian (n=93)	2.15	2.15	3.23	15.05	9.68	9.68		
			Other (n=142)	4.23	3.52	4.23	7.04	7.75	3.52		
	Camden & Islington	Trust	Clozapine (n=561)	1.78	1.96	3.74	6.06	3.92	2.85		
		Ethnic	White (n=347)	1.73	1.15	2.59	4.61	3.17	1.73		
		Background	Black (n=120)	1.67	2.50	5.83	9.17	4.17	4.17		
			Asian (n=41)	0.00	2.44	2.44	2.44	2.44	2.44		
			Others (n=53)	1.89	1.89	5.66	5.66	7.55	7.55		
	Oxford	Trust	Clozapine (n=514)	2.92	3.31	3.89	7.78	4.47	4.86		
		Ethnic	White (n=426)	3.29	3.76	4.46	8.45	5.16	4.93		
		Background	Black (n=20)	0.00	0.00	0.00	0.00	0.00	0.00		
			Asian (n=41)	0.00	0.00	0.00	4.88	2.44	2.44		
			Others (n=27)	3.70	3.70	3.70	7.41	0.00	11.11		
	SIDER	SIDER	SIDER								
Abdominalpain	SLAM	Trust	Clozapine (n=1760)	1.88	1.99	2.56	8.01	6.02	4.72		
		Ethnic	White (n=821)	1.83	1.95	2.19	6.94	4.38	3.90		
		Background	Black (n=704)	2.13	2.27	2.56	8.10	5.11	4.55		
			Asian (n=93)	1.08	1.08	1.08	8.60	9.68	4.30		
			Other (n=142)	4.23	2.82	2.82	8.45	9.15	5.63		
	Camden & Islington	Trust	Clozapine (n=561)	0.89	0.89	1.60	3.92	3.57	3.39		
		Ethnic	White (n=347)	0.58	0.86	0.86	3.46	3.17	2.88		
		Background	Black (n=120)	0.83	0.83	4.17	4.17	4.17	4.17		
			Asian (n=41)	0.00	0.00	0.00	4.88	4.88	4.88		
			Others (n=53)	1.89	1.89	1.89	5.66	5.66	3.77		
	Oxford	Trust	Clozapine (n=514)	1.75	1.36	1.75	7.39	4.47	5.64		
		Ethnic	White (n=426)	1.17	1.41	1.64	7.51	4.93	6.10		

The results are shown in percentages (%) and broken down by ADRs, Trusts (SLAM, Camden & Islington and Oxford), Cohorts, Sub Cohorts and SIDER reported values.
In Sub Cohort 'Clozapine' represent the total baseline population which further breaks down into 'White', 'Black', 'Asian' and 'Others' ethnic groups. The columns (Three Months Early, Two Months Early, One Month Early, One Month Later, Two Months Later, Three Months Later) shows the percentages in each monthly interval. The last two columns (SIDER Low End and SIDER High End) shows the SIDER reporting.

Clozapine - Ethnic Background (%)

Measure Values: 0.00 – 56.00

ADR	Trust	Cohort	Sub Cohort	Three Months Early	Two Months Early	One Month Early	One Month Later	Two Months Later	Three Months Later	SIDER Low End	SIDER High End
Abdominalpain	Oxford	Ethnic Background	Black (n=20)	5.00	0.00	0.00	5.00	5.00	0.00		
			Asian (n=41)	7.32	2.44	0.00	2.44	0.00	4.88		
			Others (n=27)	0.00	0.00	7.41	14.81	3.70	3.70		
	SIDER	SIDER	SIDER							4.00	
Convulsion	SLAM	Trust	Clozapine (n=1760)	1.36	1.70	1.82	7.05	4.94	4.03		
		Ethnic Background	White (n=821)	0.49	1.71	0.85	1.83	2.92	2.31		
			Black (n=704)	0.57	1.99	0.99	2.13	3.41	2.70		
			Asian (n=93)	3.23	1.08	3.23	8.60	3.23	2.15		
			Other (n=142)	2.11	1.41	1.41	9.15	3.52	4.23		
	Camden & Islington	Trust	Clozapine (n=561)	0.53	0.53	0.36	2.85	2.14	1.07		
		Ethnic Background	White (n=347)	0.58	0.29	0.00	2.88	2.31	1.15		
			Black (n=120)	0.83	1.67	1.67	3.33	1.67	0.83		
			Asian (n=41)	0.00	0.00	0.00	0.00	2.44	0.00		
			Others (n=53)	1.89	1.89	1.89	5.66	3.77	3.77		
	Oxford	Trust	Clozapine (n=514)	1.36	1.36	1.56	6.42	3.11	2.72		
		Ethnic Background	White (n=426)	1.64	1.41	1.88	6.34	3.05	3.29		
			Black (n=20)	0.00	0.00	0.00	10.00	10.00	0.00		
			Asian (n=41)	0.00	0.00	0.00	4.88	2.44	0.00		
			Others (n=27)	0.00	3.70	0.00	7.41	0.00	0.00		
	SIDER	SIDER	SIDER							3.00	
Hypotension	SLAM	Trust	Clozapine (n=1760)	0.51	0.97	0.80	5.00	2.95	2.56		
		Ethnic Background	White (n=821)	0.49	0.73	0.37	3.78	1.46	1.46		
			Black (n=704)	0.57	0.85	0.43	4.40	1.70	1.70		
			Asian (n=93)	0.00	1.08	2.15	6.45	2.15	3.23		
			Other (n=142)	0.70	2.11	2.11	9.86	4.93	2.11		
	Camden & Islington	Trust	Clozapine (n=561)	0.18	0.53	0.18	3.57	2.32	1.78		
		Ethnic Background	White (n=347)	0.29	0.58	0.29	4.90	2.59	2.59		
			Black (n=120)	0.00	0.83	0.00	0.83	3.33	0.00		
			Asian (n=41)	0.00	0.00	0.00	4.88	0.00	0.00		
			Others (n=53)	0.00	0.00	0.00	0.00	0.00	1.89		
	Oxford	Trust	Clozapine (n=514)	0.58	0.78	0.78	5.64	3.50	2.92		
		Ethnic Background	White (n=426)	0.47	0.94	0.94	5.63	3.29	3.29		
			Black (n=20)	0.00	0.00	0.00	10.00	0.00	0.00		
			Asian (n=41)	0.00	0.00	0.00	0.00	4.88	0.00		
			Others (n=27)	3.70	0.00	0.00	11.11	7.41	3.70		
	SIDER	SIDER	SIDER							9.00	38.00
Nausea	SLAM	Trust	Clozapine (n=1760)	1.14	1.08	1.19	6.08	5.23	3.69		
		Ethnic Background	White (n=821)	1.58	0.97	0.97	3.78	4.14	2.44		
			Black (n=704)	1.85	1.14	1.14	4.40	4.83	2.84		
			Asian (n=93)	2.15	1.08	2.15	8.60	7.53	2.15		
			Other (n=142)	0.70	1.41	0.00	5.63	8.45	3.52		
	Camden & Islington	Trust	Clozapine (n=561)	0.89	1.43	0.36	4.63	3.57	3.57		
		Ethnic Background	White (n=347)	0.86	1.73	0.00	5.19	4.32	2.88		
			Black (n=120)	0.00	1.67	0.00	5.00	0.83	5.00		
			Asian (n=41)	0.00	0.00	2.44	2.44	4.88	4.88		
			Others (n=53)	1.89	1.89	0.00	3.77	3.77	1.89		
	Oxford	Trust	Clozapine (n=514)	0.97	0.58	1.36	4.86	3.70	2.92		
		Ethnic Background	White (n=426)	0.94	0.70	1.41	4.46	3.29	3.05		
			Black (n=20)	0.00	0.00	0.00	0.00	0.00	5.00		
			Asian (n=41)	2.44	0.00	0.00	9.76	9.76	2.44		
			Others (n=27)	0.00	0.00	3.70	7.41	3.70	0.00		
	SIDER	SIDER	SIDER							3.00	17.00
Backache	SLAM	Trust	Clozapine (n=1760)	1.14	1.59	2.44	4.94	3.35	2.73		
		Ethnic Background	White (n=821)	0.85	1.10	1.58	5.36	2.56	1.34		
			Black (n=704)	0.99	1.28	1.85	6.25	2.98	1.56		
			Asian (n=93)	3.23	4.30	3.23	7.53	5.38	4.30		
			Other (n=142)	3.52	0.70	2.11	6.34	4.23	2.82		
	Camden & Islington	Trust	Clozapine (n=561)	1.43	1.25	1.96	5.35	3.03	3.03		
		Ethnic Background	White (n=347)	1.73	1.15	2.02	6.05	3.46	2.31		
			Black (n=120)	0.83	2.50	3.33	6.67	3.33	5.83		
			Asian (n=41)	2.44	0.00	0.00	0.00	2.44	2.44		
			Others (n=53)	0.00	0.00	0.00	1.89	0.00	1.89		
	Oxford	Trust	Clozapine (n=514)	1.17	1.17	1.36	5.84	3.89	2.92		
		Ethnic Background	White (n=426)	0.94	1.41	1.41	6.10	4.46	3.29		
			Black (n=20)	5.00	0.00	0.00	5.00	0.00	0.00		
			Asian (n=41)	0.00	0.00	0.00	4.88	0.00	0.00		
			Others (n=27)	3.70	0.00	3.70	3.70	3.70	3.70		

The results are shown in percentages (%) and broken down by ADRs, Trusts (SLAM, Camden & Islington and Oxford), Cohorts, Sub Cohorts and SIDER reported values.
In Sub Cohort 'Clozapine' represent the total baseline population which further breaks down into 'White', 'Black', 'Asian' and 'Others' ethnic groups. The columns (Three Months Early, Two Months Early, One Month Early, One Month Later, Two Months Later, Three Months Later) shows the percentages in each monthly interval. The last two columns (SIDER Low End and SIDER High End) shows the SIDER reporting.

Clozapine - Ethnic Background (%)

Measure Values: 0.00 – 56.00

ADR	Trust	Cohort	Sub Cohort	Three Months Early	Two Months Early	One Month Early	One Month Later	Two Months Later	Three Months Later	SIDER Low End	SIDER High End
Backache	SIDER	SIDER	SIDER							5.00	
Fever	SLAM	Trust	Clozapine (n=1760)	1.02	1.14	1.65	6.36	4.43	3.13		
		Ethnic Background	White (n=821)	1.34	1.34	1.58	5.97	4.14	2.92		
			Black (n=704)	1.56	1.56	1.85	6.96	4.83	3.41		
			Asian (n=93)	1.08	0.00	2.15	6.45	3.23	1.08		
			Other (n=142)	0.70	0.70	0.70	9.15	6.34	3.52		
	Camden & Islington	Trust	Clozapine (n=561)	0.89	0.89	0.53	3.74	2.67	0.89		
		Ethnic Background	White (n=347)	1.15	1.15	0.29	3.17	2.88	0.58		
			Black (n=120)	0.83	0.83	0.83	5.83	3.33	2.50		
			Asian (n=41)	0.00	0.00	0.00	0.00	2.44	0.00		
			Others (n=53)	0.00	0.00	1.89	5.66	3.77	1.89		
	Oxford	Trust	Clozapine (n=514)	0.39	0.78	0.58	3.11	2.72	2.33		
		Ethnic Background	White (n=426)	0.23	0.70	0.47	3.05	2.82	2.58		
			Black (n=20)	0.00	5.00	0.00	0.00	0.00	0.00		
			Asian (n=41)	0.00	0.00	2.44	4.88	2.44	0.00		
			Others (n=27)	3.70	0.00	0.00	3.70	3.70	3.70		
	SIDER	SIDER	SIDER							4.00	13.00
Enuresis	SLAM	Trust	Clozapine (n=1760)	1.02	0.80	1.25	4.20	3.92	3.24		
		Ethnic Background	White (n=821)	1.10	0.85	1.46	3.41	2.80	2.07		
			Black (n=704)	1.28	0.99	1.70	3.98	3.27	2.41		
			Asian (n=93)	1.08	1.08	2.15	7.53	3.23	1.08		
			Other (n=142)	1.41	0.00	1.41	4.93	5.63	1.41		
	Camden & Islington	Trust	Clozapine (n=561)	0.71	1.07	1.07	4.10	1.43	1.25		
		Ethnic Background	White (n=347)	0.58	0.86	0.86	3.17	1.73	1.73		
			Black (n=120)	0.00	1.67	1.67	6.67	0.83	0.83		
			Asian (n=41)	2.44	2.44	2.44	7.32	2.44	0.00		
			Others (n=53)	1.89	0.00	0.00	0.00	0.00	0.00		
	Oxford	Trust	Clozapine (n=514)	1.36	0.58	1.36	4.86	4.47	3.50		
		Ethnic Background	White (n=426)	1.41	0.47	1.41	5.16	4.23	2.82		
			Black (n=20)	0.00	0.00	0.00	5.00	5.00	10.00		
			Asian (n=41)	2.44	2.44	2.44	2.44	7.32	4.88		
			Others (n=27)	0.00	0.00	0.00	3.70	3.70	7.41		
	SIDER	SIDER	SIDER								
Drymouth	SLAM	Trust	Clozapine (n=1760)	1.08	1.53	1.65	4.66	3.69	2.33		
		Ethnic Background	White (n=821)	1.22	1.34	1.71	3.53	2.68	1.95		
			Black (n=704)	1.42	1.56	1.99	4.12	3.13	2.27		
			Asian (n=93)	0.00	4.30	0.00	5.38	6.45	3.23		
			Other (n=142)	2.11	3.52	1.41	9.86	3.52	2.11		
	Camden & Islington	Trust	Clozapine (n=561)	1.25	1.25	1.07	3.92	2.14	0.89		
		Ethnic Background	White (n=347)	1.44	1.15	0.86	4.32	2.88	0.86		
			Black (n=120)	0.00	0.00	0.83	2.50	1.67	0.83		
			Asian (n=41)	0.00	4.88	2.44	7.32	0.00	0.00		
			Others (n=53)	3.77	1.89	1.89	3.77	1.89	1.89		
	Oxford	Trust	Clozapine (n=514)	1.36	1.36	1.56	3.89	1.36	2.33		
		Ethnic Background	White (n=426)	1.41	1.41	1.88	4.46	1.41	2.58		
			Black (n=20)	0.00	0.00	0.00	0.00	0.00	0.00		
			Asian (n=41)	0.00	2.44	0.00	2.44	2.44	0.00		
			Others (n=27)	3.70	0.00	0.00	0.00	0.00	3.70		
	SIDER	SIDER	SIDER							5.00	20.00
Diarrhoea	SLAM	Trust	Clozapine (n=1760)	1.08	1.31	1.36	4.72	3.58	2.56		
		Ethnic Background	White (n=821)	0.73	1.10	1.10	3.29	2.19	1.95		
			Black (n=704)	0.85	1.28	1.28	3.84	2.56	2.27		
			Asian (n=93)	1.08	1.08	2.15	2.15	2.15	0.00		
			Other (n=142)	3.52	2.11	0.70	4.93	4.93	2.82		
	Camden & Islington	Trust	Clozapine (n=561)	0.71	1.25	0.18	3.03	3.39	3.03		
		Ethnic Background	White (n=347)	0.29	1.44	0.29	3.75	3.46	3.75		
			Black (n=120)	0.83	1.67	0.00	3.33	5.00	2.50		
			Asian (n=41)	2.44	0.00	0.00	0.00	0.00	0.00		
			Others (n=53)	0.00	0.00	0.00	0.00	1.89	1.89		
	Oxford	Trust	Clozapine (n=514)	1.17	0.78	1.36	4.09	3.70	2.53		
		Ethnic Background	White (n=426)	1.17	0.47	1.41	3.76	3.52	3.05		
			Black (n=20)	0.00	5.00	0.00	10.00	10.00	0.00		
			Asian (n=41)	2.44	0.00	2.44	2.44	2.44	0.00		
			Others (n=27)	0.00	3.70	0.00	7.41	3.70	0.00		
	SIDER	SIDER	SIDER							2.00	
Dyspepsia	SLAM	Trust	Clozapine (n=1760)	0.74	1.08	0.91	3.92	3.13	3.69		
		Ethnic	White (n=821)	0.37	1.34	0.97	3.78	2.19	2.92		

The results are shown in percentages (%) and broken down by ADRs, Trusts (SLAM, Camden & Islington and Oxford), Cohorts, Sub Cohorts and SIDER reported values.
In Sub Cohort 'Clozapine' represent the total baseline population which further breaks down into 'White', 'Black', 'Asian' and 'Others' ethnic groups.
The columns (Three Months Early, Two Months Early, One Month Early, One Month Later, Two Months Later, Three Months Later) shows the percentages in each monthly interval. The last two columns (SIDER Low End and SIDER High End) shows the SIDER reporting.

Clozapine - Ethnic Background (%)

Measure Values
0.00 56.00

ADR	Trust	Cohort	Sub Cohort	Three Months Early	Two Months Early	One Month Early	One Month Later	Two Months Later	Three Months Later	SIDER Low End	SIDER High End
**Dyspepsia**	SLAM	Ethnic Background	Black (n=704)	0.43	1.56	1.14	4.40	2.56	3.41		
			Asian (n=93)	2.15	2.15	0.00	1.08	5.38	1.08		
			Other (n=142)	2.82	0.70	0.70	2.11	2.11	4.23		
	Camden & Islington	Trust	Clozapine (n=561)	0.36	0.53	0.53	4.10	2.67	2.50		
		Ethnic Background	White (n=347)	0.29	0.86	0.29	4.32	2.59	2.88		
			Black (n=120)	0.00	0.00	1.67	2.50	2.50	0.00		
			Asian (n=41)	0.00	0.00	0.00	9.76	7.32	2.44		
			Others (n=53)	1.89	0.00	0.00	1.89	0.00	5.66		
	Oxford	Trust	Clozapine (n=514)	0.19	0.58	0.78	3.50	4.09	3.70		
		Ethnic Background	White (n=426)	0.23	0.70	0.70	3.52	4.69	3.99		
			Black (n=20)	0.00	0.00	0.00	0.00	0.00	0.00		
			Asian (n=41)	0.00	0.00	0.00	2.44	0.00	2.44		
			Others (n=27)	0.00	0.00	3.70	7.41	3.70	3.70		
	SIDER	SIDER	SIDER							8.00	14.00
**Rash**	SLAM	Trust	Clozapine (n=1760)	1.25	1.59	2.05	3.64	2.95	2.27		
		Ethnic Background	White (n=821)	0.73	1.34	1.22	1.83	1.46	1.34		
			Black (n=704)	0.85	1.56	1.42	2.13	1.70	1.56		
			Asian (n=93)	0.00	3.23	4.30	7.53	0.00	2.15		
			Other (n=142)	4.23	1.41	3.52	3.52	1.41	1.41		
	Camden & Islington	Trust	Clozapine (n=561)	1.25	1.25	0.89	4.28	1.96	2.14		
		Ethnic Background	White (n=347)	1.15	1.15	1.15	4.90	2.31	3.17		
			Black (n=120)	0.00	0.83	0.00	2.50	0.00	0.00		
			Asian (n=41)	4.88	2.44	2.44	4.88	4.88	2.44		
			Others (n=53)	1.89	1.89	0.00	1.89	0.00	0.00		
	Oxford	Trust	Clozapine (n=514)	0.97	1.17	1.17	3.70	2.33	1.36		
		Ethnic Background	White (n=426)	1.17	1.41	1.41	3.99	2.58	1.41		
			Black (n=20)	0.00	0.00	0.00	5.00	5.00	5.00		
			Asian (n=41)	0.00	0.00	0.00	2.44	0.00	0.00		
			Others (n=27)	0.00	0.00	0.00	0.00	0.00	0.00		
	SIDER	SIDER	SIDER								
**Stomachpain**	SLAM	Trust	Clozapine (n=1760)	1.93	1.76	1.93	4.94	3.52	3.52		
		Ethnic Background	White (n=821)	1.71	1.58	1.58	4.14	3.29	2.68		
			Black (n=704)	1.99	1.85	1.85	4.83	3.84	3.13		
			Asian (n=93)	3.23	3.23	1.08	3.23	3.23	2.15		
			Other (n=142)	3.52	3.52	3.52	6.34	5.63	2.82		
	Camden & Islington	Trust	Clozapine (n=561)	0.89	1.25	0.89	3.39	2.85	2.14		
		Ethnic Background	White (n=347)	0.29	1.73	0.58	2.59	2.31	1.73		
			Black (n=120)	2.50	0.83	2.50	7.50	5.83	4.17		
			Asian (n=41)	0.00	0.00	0.00	2.44	0.00	0.00		
			Others (n=53)	5.66	0.00	0.00	1.89	1.89	1.89		
	Oxford	Trust	Clozapine (n=514)	1.56	0.78	0.78	2.14	0.97	0.97		
		Ethnic Background	White (n=426)	1.41	0.94	0.47	2.58	1.17	1.17		
			Black (n=20)	0.00	0.00	5.00	0.00	0.00	0.00		
			Asian (n=41)	2.44	0.00	0.00	0.00	0.00	0.00		
			Others (n=27)	3.70	0.00	3.70	0.00	0.00	0.00		
	SIDER	SIDER	SIDER								
**Tremor**	SLAM	Trust	Clozapine (n=1760)	1.48	1.99	2.95	5.51	3.52	3.47		
		Ethnic Background	White (n=821)	1.10	1.71	2.19	3.29	2.92	2.44		
			Black (n=704)	1.28	1.99	2.56	3.84	3.41	2.84		
			Asian (n=93)	1.08	2.15	1.08	8.60	4.30	5.38		
			Other (n=142)	2.82	2.82	3.52	4.93	4.23	4.23		
	Camden & Islington	Trust	Clozapine (n=561)	1.60	1.78	2.14	3.92	1.96	2.14		
		Ethnic Background	White (n=347)	1.44	0.58	0.86	3.75	1.73	1.73		
			Black (n=120)	2.50	3.33	5.00	3.33	2.50	2.50		
			Asian (n=41)	0.00	0.00	0.00	0.00	0.00	2.44		
			Others (n=53)	1.89	3.77	3.77	7.55	3.77	3.77		
	SIDER	SIDER	SIDER							6.00	
**Sweating**	SLAM	Trust	Clozapine (n=1760)	1.08	0.97	1.36	4.43	4.26	2.84		
		Ethnic Background	White (n=821)	0.61	0.85	1.46	4.14	3.29	1.95		
			Black (n=704)	0.71	0.99	1.70	4.83	3.84	2.27		
			Asian (n=93)	2.15	0.00	1.08	0.00	1.08	3.23		
			Other (n=142)	2.82	0.70	0.70	2.11	2.11	2.11		
	Camden & Islington	Trust	Clozapine (n=561)	0.53	0.53	0.53	2.85	2.14	1.96		
		Ethnic Background	White (n=347)	0.00	0.58	0.29	2.02	2.31	2.02		
			Black (n=120)	1.67	0.83	0.83	5.00	2.50	2.50		
			Asian (n=41)	0.00	0.00	2.44	4.88	0.00	0.00		
			Others (n=53)	1.89	0.00	0.00	1.89	1.89	1.89		

The results are shown in percentages (%) and broken down by ADRs, Trusts (SLAM, Camden & Islington and Oxford), Cohorts, Sub Cohorts and SIDER reported values.
In Sub Cohort 'Clozapine' represent the total baseline population which further breaks down into 'White', 'Black', 'Asian' and 'Others' ethnic groups. The columns (Three Months Early, Two Months Early, One Month Early, One Month Later, Two Months Later, Three Months Later) shows the percentages in each monthly interval. The last two columns (SIDER Low End and SIDER High End) shows the SIDER reporting.

Clozapine - Ethnic Background (%)

Measure Values: 0.00 – 56.00

ADR	Trust	Cohort	Sub Cohort	Three Months Early	Two Months Early	One Month Early	One Month Later	Two Months Later	Three Months Later	SIDER Low End	SIDER High End
**Sweating**	Oxford	Trust	Clozapine (n=514)	1.17	0.97	0.97	2.72	1.36	1.95		
		Ethnic Background	White (n=426)	0.94	0.94	0.94	3.05	1.17	1.64		
			Black (n=20)	0.00	0.00	0.00	0.00	0.00	5.00		
			Asian (n=41)	0.00	2.44	2.44	2.44	4.88	4.88		
			Others (n=27)	7.41	0.00	0.00	0.00	0.00	0.00		
	SIDER	SIDER	SIDER							6.00	
**Neutropenia**	SLAM	Trust	Clozapine (n=1760)	0.80	0.80	0.74	5.34	2.73	2.61		
		Ethnic Background	White (n=821)	1.10	0.73	0.85	5.85	3.41	3.17		
			Black (n=704)	1.28	0.85	0.99	6.82	3.98	3.69		
			Asian (n=93)	0.00	1.08	1.08	3.23	2.15	2.15		
			Other (n=142)	0.70	0.00	0.70	6.34	1.41	1.41		
	Camden & Islington	Trust	Clozapine (n=561)	0.00	0.18	0.53	1.60	0.89	1.07		
		Ethnic Background	White (n=347)	0.00	0.00	0.00	0.58	0.29	0.86		
			Black (n=120)	0.00	0.83	2.50	4.17	3.33	2.50		
			Asian (n=41)	0.00	0.00	0.00	2.44	0.00	0.00		
			Others (n=53)	0.00	0.00	0.00	1.89	0.00	0.00		
	SIDER	SIDER	SIDER								
**Blurredvision**	SLAM	Trust	Clozapine (n=1760)	0.34	0.91	0.63	2.05	1.25	1.02		
		Ethnic Background	White (n=821)	0.49	0.97	0.49	1.34	1.58	1.10		
			Black (n=704)	0.57	1.14	0.57	1.56	1.85	1.28		
			Asian (n=93)	0.00	1.08	0.00	1.08	0.00	0.00		
			Other (n=142)	0.00	0.70	0.70	2.82	2.11	0.70		
	Camden & Islington	Trust	Clozapine (n=561)	0.89	0.53	0.71	1.25	0.36	0.89		
		Ethnic Background	White (n=347)	0.86	0.29	0.58	1.44	0.58	0.86		
			Black (n=120)	1.67	1.67	1.67	1.67	0.00	1.67		
			Asian (n=41)	0.00	0.00	0.00	0.00	0.00	0.00		
			Others (n=53)	0.00	0.00	0.00	0.00	0.00	0.00		
	Oxford	Trust	Clozapine (n=514)	0.19	0.39	0.39	1.56	1.56	1.17		
		Ethnic Background	White (n=426)	0.23	0.47	0.47	1.64	1.64	1.41		
			Black (n=20)	0.00	0.00	0.00	0.00	0.00	0.00		
			Asian (n=41)	0.00	0.00	0.00	0.00	2.44	0.00		
			Others (n=27)	0.00	0.00	0.00	3.70	0.00	0.00		
	SIDER	SIDER	SIDER							5.00	
**Akathisia**	SLAM	Trust	Clozapine (n=1760)	0.80	0.91	0.74	2.67	1.36	0.80		
		Ethnic Background	White (n=821)	0.49	0.49	0.24	1.58	0.73	0.61		
			Black (n=704)	0.57	0.57	0.28	1.85	0.85	0.71		
			Asian (n=93)	1.08	2.15	3.23	4.30	2.15	1.08		
			Other (n=142)	0.70	0.00	0.00	0.70	0.00	0.70		
	Camden & Islington	Trust	Clozapine (n=561)	0.00	0.53	0.00	1.25	1.07	0.53		
		Ethnic Background	White (n=347)	0.00	0.29	0.00	1.15	0.86	0.29		
			Black (n=120)	0.00	0.00	0.00	0.83	0.00	0.00		
			Asian (n=41)	0.00	0.00	0.00	2.44	2.44	0.00		
			Others (n=53)	0.00	1.89	0.00	0.00	3.77	3.77		
	Oxford	Trust	Clozapine (n=514)	0.97	0.78	0.97	1.36	1.17	0.97		
		Ethnic Background	White (n=426)	0.70	0.70	0.94	1.64	1.41	1.17		
			Black (n=20)	0.00	0.00	0.00	0.00	0.00	0.00		
			Asian (n=41)	4.88	0.00	2.44	0.00	0.00	0.00		
			Others (n=27)	0.00	3.70	0.00	0.00	0.00	0.00		
	SIDER	SIDER	SIDER							3.00	

The results are shown in percentages (%) and broken down by ADRs, Trusts (SLAM, Camden & Islington and Oxford), Cohorts, Sub Cohorts and SIDER reported values.
In Sub Cohort ‘Clozapine’ represent the total baseline population which further breaks down into ‘White’, ‘Black’, ‘Asian’ and ‘Others’ ethnic groups. The columns (Three Months Early, Two Months Early, One Month Early, One Month Later, Two Months Later, Three Months Later) shows the percentages in each monthly interval. The last two columns (SIDER Low End and SIDER High End) shows the SIDER reporting.

Clozapine - Age Groups (%)

ADR	Trust	Cohort	Sub Cohort	Three Months Early	Two Months Early	One Month Early	One Month Later	Two Months Later	Three Months Later	SIDER Low End	SIDER High End
Agitation	SLAM	Trust	Clozapine (n=1760)	17.61	22.10	26.53	46.59	32.56	26.99		
		Age Groups	Under 21 (n=57)	31.58	47.37	40.35	75.44	50.88	40.35		
			21-30 (n=422)	20.62	27.96	34.36	55.69	38.86	37.68		
			31-40 (n=488)	17.83	20.08	23.77	45.29	33.20	25.41		
			41-50 (n=479)	16.08	19.21	24.63	42.80	28.60	22.55		
			51-60 (n=233)	12.88	15.88	20.17	37.34	26.18	17.60		
			61-70 (n=62)	12.90	20.97	19.35	35.48	25.81	24.19		
			71-80 (n=18)	16.67	16.67	27.78	33.33	16.67	27.78		
	Camden & Islington	Trust	Clozapine (n=561)	13.37	17.83	18.36	43.14	28.34	21.03		
		Age Groups	21-30 (n=27)	40.74	55.56	59.26	66.67	37.04	40.74		
			31-40 (n=141)	20.57	24.82	18.44	47.52	34.75	29.79		
			41-50 (n=168)	8.33	13.10	16.07	40.48	25.60	20.24		
			51-60 (n=135)	9.63	11.11	12.59	37.78	23.70	13.33		
			61-70 (n=67)	7.46	13.43	19.40	41.79	28.36	17.91		
			71-80 (n=21)	14.29	19.05	19.05	42.86	28.57	4.76		
	SIDER	SIDER	SIDER							4.00	
Fatigue	SLAM	Trust	Clozapine (n=1760)	12.67	14.83	15.85	43.58	35.80	30.51		
		Age Groups	Under 21 (n=57)	26.32	28.07	29.82	68.42	68.42	49.12		
			21-30 (n=422)	18.01	20.85	21.80	53.79	43.84	40.76		
			31-40 (n=488)	11.68	12.50	15.78	43.65	33.40	28.89		
			41-50 (n=479)	10.02	13.36	13.57	39.25	30.69	24.84		
			51-60 (n=233)	8.15	10.30	9.01	31.33	33.05	24.03		
			61-70 (n=62)	6.45	6.45	9.68	35.48	25.81	30.65		
			71-80 (n=18)	22.22	22.22	5.56	22.22	11.11	5.56		
	Camden & Islington	Trust	Clozapine (n=561)	10.34	12.30	13.37	41.18	29.23	26.56		
		Age Groups	21-30 (n=27)	25.93	44.44	55.56	59.26	33.33	40.74		
			31-40 (n=141)	14.18	17.73	15.60	45.39	33.33	32.62		
			41-50 (n=168)	4.17	7.74	7.14	42.26	29.17	24.40		
			51-60 (n=135)	11.11	6.67	12.59	31.11	22.96	22.96		
			61-70 (n=67)	11.94	10.45	10.45	46.27	32.84	25.37		
			71-80 (n=21)	4.76	14.29	9.52	38.10	28.57	19.05		
	SIDER	SIDER	SIDER								
Sedation	SLAM	Trust	Clozapine (n=1760)	12.67	12.16	14.83	43.86	35.51	29.83		
		Age Groups	Under 21 (n=57)	26.32	26.32	28.07	66.67	61.40	43.86		
			21-30 (n=422)	18.48	18.96	21.80	52.13	46.21	38.63		
			31-40 (n=488)	11.68	10.66	13.32	44.47	33.61	29.92		
			41-50 (n=479)	10.23	8.98	10.65	39.46	30.69	26.72		
			51-60 (n=233)	8.15	7.30	11.59	33.05	25.75	19.31		
			61-70 (n=62)	3.23	4.84	11.29	38.71	32.26	24.19		
			71-80 (n=18)	16.67	16.67	16.67	33.33	16.67	11.11		
	Camden & Islington	Trust	Clozapine (n=561)	5.17	9.09	9.09	38.15	26.56	21.93		
		Age Groups	21-30 (n=27)	18.52	25.93	37.04	48.15	25.93	40.74		
			31-40 (n=141)	9.22	14.18	13.48	41.84	34.04	26.95		
			41-50 (n=168)	2.98	6.55	7.14	39.29	25.60	25.00		
			51-60 (n=135)	3.70	4.44	5.19	28.89	22.96	14.07		
			61-70 (n=67)	1.49	8.96	2.99	43.28	26.87	14.93		
			71-80 (n=21)	0.00	4.76	4.76	33.33	14.29	19.05		
	SIDER	SIDER	SIDER							25.00	46.00
Dizziness	SLAM	Trust	Clozapine (n=1760)	2.78	4.20	4.09	16.59	13.13	11.19		
		Age Groups	Under 21 (n=57)	7.02	1.75	5.26	28.07	21.05	15.79		
			21-30 (n=422)	2.84	4.98	4.98	18.25	12.80	12.32		
			31-40 (n=488)	2.05	4.51	2.87	14.14	12.50	8.20		
			41-50 (n=479)	3.13	4.59	3.34	15.03	12.11	10.02		
			51-60 (n=233)	3.00	2.15	5.15	16.74	14.16	15.02		
			61-70 (n=62)	0.00	3.23	8.06	22.58	17.74	16.13		
			71-80 (n=18)	5.56	5.56	5.56	27.78	11.11	11.11		
	Camden & Islington	Trust	Clozapine (n=561)	3.21	3.39	3.74	18.18	13.73	9.09		
		Age Groups	21-30 (n=27)	14.81	14.81	14.81	25.93	14.81	18.52		
			31-40 (n=141)	2.13	4.96	7.80	15.60	20.57	6.38		
			41-50 (n=168)	2.98	1.79	1.19	14.29	7.74	8.93		
			51-60 (n=135)	1.48	1.48	2.22	21.48	13.33	10.37		
			61-70 (n=67)	4.48	4.48	0.00	23.88	17.91	7.46		
			71-80 (n=21)	4.76	0.00	4.76	19.05	9.52	19.05		
	SIDER	SIDER	SIDER							12.00	27.00
Confusion	SLAM	Trust	Clozapine (n=1760)	4.72	5.51	6.08	13.92	8.47	6.76		
		Age Groups	Under 21 (n=57)	8.77	5.26	12.28	21.05	22.81	12.28		

Measure Values
0.00 75.44

The results are shown in percentages (%) and broken down by ADRs, Trusts (SLAM, Camden & Islington and Oxford), Cohorts, Sub Cohorts and SIDER reported values.
In Sub Cohort 'Clozapine' represent the total baseline population which further breaks down into age groups.
The columns (Three Months Early, Two Months Early, One Month Early, One Month Later, Two Months Later, Three Months Later) shows the percentages in each monthly interval. The last two columns (SIDER Low End and SIDER High End) shows the SIDER reporting.

Clozapine - Age Groups (%)

ADR	Trust	Cohort	Sub Cohort	Three Months Early	Two Months Early	One Month Early	One Month Later	Two Months Later	Three Months Later	SIDER Low End	SIDER High End
Confusion	SLAM	Age Groups	21-30 (n=422)	5.69	6.40	6.16	13.27	8.77	6.64		
			31-40 (n=488)	3.69	5.12	6.76	11.89	7.99	6.76		
			41-50 (n=479)	4.38	4.80	4.59	13.78	7.52	6.26		
			51-60 (n=233)	4.72	4.29	5.58	17.17	7.30	8.15		
			61-70 (n=62)	4.84	9.68	8.06	19.35	11.29	3.23		
			71-80 (n=18)	5.56	11.11	5.56	5.56	0.00	0.00		
	Camden & Islington	Trust	Clozapine (n=561)	3.57	6.24	5.53	12.66	6.77	5.88		
		Age Groups	21-30 (n=27)	7.41	25.93	22.22	33.33	3.70	7.41		
			31-40 (n=141)	4.96	9.22	2.84	9.93	7.09	6.38		
			41-50 (n=168)	2.98	4.17	4.17	9.52	5.36	7.14		
			51-60 (n=135)	1.48	1.48	5.19	12.59	8.15	4.44		
			61-70 (n=67)	4.48	7.46	8.96	19.40	8.96	5.97		
			71-80 (n=21)	4.76	4.76	4.76	9.52	0.00	0.00		
	SIDER	SIDER	SIDER							3.00	
Constipation	SLAM	Trust	Clozapine (n=1760)	1.76	1.99	2.16	12.27	11.70	9.49		
		Age Groups	Under 21 (n=57)	1.75	5.26	7.02	19.30	15.79	15.79		
			21-30 (n=422)	1.66	0.24	1.90	13.27	13.27	9.24		
			31-40 (n=488)	1.84	1.84	1.64	9.22	9.22	10.04		
			41-50 (n=479)	1.46	2.51	2.51	11.90	9.39	7.10		
			51-60 (n=233)	1.72	3.00	2.15	12.88	15.88	9.44		
			61-70 (n=62)	1.61	4.84	1.61	19.35	17.74	17.74		
			71-80 (n=18)	11.11	0.00	0.00	27.78	16.67	16.67		
	Camden & Islington	Trust	Clozapine (n=561)	1.07	2.50	1.78	11.41	7.13	5.70		
		Age Groups	21-30 (n=27)	0.00	3.70	3.70	0.00	7.41	0.00		
			31-40 (n=141)	1.42	2.84	1.42	12.06	4.96	6.38		
			41-50 (n=168)	0.60	1.19	1.19	9.52	7.14	5.95		
			51-60 (n=135)	0.74	1.48	0.74	10.37	7.41	5.93		
			61-70 (n=67)	1.49	7.46	4.48	17.91	10.45	5.97		
			71-80 (n=21)	4.76	0.00	4.76	23.81	9.52	4.76		
	SIDER	SIDER	SIDER							10.00	25.00
Hypersalivation	SLAM	Trust	Clozapine (n=1760)	1.19	1.48	2.10	14.32	13.24	11.31		
		Age Groups	Under 21 (n=57)	0.00	3.51	3.51	24.56	15.79	19.30		
			21-30 (n=422)	2.37	1.90	3.55	17.30	13.03	14.45		
			31-40 (n=488)	1.23	1.02	1.23	14.55	14.34	12.70		
			41-50 (n=479)	0.84	1.46	1.25	10.65	11.48	7.31		
			51-60 (n=233)	0.43	1.72	2.58	14.59	14.16	9.01		
			61-70 (n=62)	0.00	0.00	3.23	9.68	14.52	14.52		
			71-80 (n=18)	0.00	0.00	0.00	16.67	11.11	0.00		
	Camden & Islington	Trust	Clozapine (n=561)	1.07	1.43	0.53	14.26	6.95	7.66		
		Age Groups	21-30 (n=27)	3.70	3.70	0.00	7.41	3.70	7.41		
			31-40 (n=141)	2.84	3.55	0.71	17.73	12.06	10.64		
			41-50 (n=168)	0.00	0.00	1.19	12.50	4.17	4.17		
			51-60 (n=135)	0.00	0.00	0.00	14.81	7.41	9.63		
			61-70 (n=67)	1.49	2.99	0.00	17.91	5.97	7.46		
			71-80 (n=21)	0.00	0.00	0.00	0.00	0.00	9.52		
	SIDER	SIDER	SIDER							1.00	48.00
Tachycardia	SLAM	Trust	Clozapine (n=1760)	2.27	2.05	2.50	15.40	12.95	9.94		
		Age Groups	Under 21 (n=57)	10.53	8.77	7.02	36.84	35.09	28.07		
			21-30 (n=422)	3.55	2.84	3.08	21.56	18.48	16.59		
			31-40 (n=488)	1.43	1.43	1.84	14.14	11.89	8.20		
			41-50 (n=479)	1.67	2.09	1.88	12.32	9.81	5.64		
			51-60 (n=233)	0.86	0.43	3.00	11.59	7.73	6.44		
			61-70 (n=62)	0.00	0.00	0.00	4.84	8.06	8.06		
			71-80 (n=18)	11.11	5.56	11.11	5.56	11.11	11.11		
	Camden & Islington	Trust	Clozapine (n=561)	1.43	1.43	0.89	11.23	8.38	6.95		
		Age Groups	21-30 (n=27)	11.11	3.70	7.41	25.93	11.11	14.81		
			31-40 (n=141)	0.71	1.42	0.71	13.48	11.35	9.93		
			41-50 (n=168)	1.19	1.79	0.00	7.14	8.93	4.76		
			51-60 (n=135)	0.00	0.74	0.00	11.85	5.93	5.93		
			61-70 (n=67)	2.99	1.49	2.99	13.43	5.97	7.46		
			71-80 (n=21)	0.00	0.00	0.00	0.00	4.76	0.00		
	SIDER	SIDER	SIDER							11.00	25.00
Weightgain	SLAM	Trust	Clozapine (n=1760)	3.75	4.43	5.06	15.34	10.91	10.34		
		Age Groups	Under 21 (n=57)	10.53	14.04	14.04	31.58	28.07	29.82		
			21-30 (n=422)	5.92	7.58	7.35	19.43	15.17	14.22		
			31-40 (n=488)	4.30	3.48	5.12	20.49	12.50	9.43		

Measure Values
0.00 75.44

The results are shown in percentages (%) and broken down by ADRs, Trusts (SLAM, Camden & Islington and Oxford), Cohorts, Sub Cohorts and SIDER reported values.
In Sub Cohort 'Clozapine' represent the total baseline population which further breaks down into age groups.
The columns (Three Months Early, Two Months Early, One Month Early, One Month Later, Two Months Later, Three Months Later) shows the percentages in each monthly interval. The last two columns (SIDER Low End and SIDER High End) shows the SIDER reporting.

Clozapine - Age Groups (%)

Measure Values: 0.00 – 75.44

ADR	Trust	Cohort	Sub Cohort	Three Months Early	Two Months Early	One Month Early	One Month Later	Two Months Later	Three Months Later	SIDER Low End	SIDER High End
**Weightgain**	SLAM	Age Groups	41-50 (n=479)	2.30	2.51	3.34	10.86	7.31	7.93		
			51-60 (n=233)	0.86	3.00	3.00	6.44	4.29	6.87		
			61-70 (n=62)	1.61	1.61	0.00	4.84	8.06	4.84		
			71-80 (n=18)	0.00	5.56	11.11	0.00	5.56	11.11		
	Camden & Islington	Trust	Clozapine (n=561)	2.50	3.39	1.96	11.76	6.60	6.24		
		Age Groups	21-30 (n=27)	0.00	7.41	0.00	18.52	22.22	11.11		
			31-40 (n=141)	2.84	4.26	3.55	11.35	7.80	9.22		
			41-50 (n=168)	2.38	2.98	1.19	11.31	5.36	5.95		
			51-60 (n=135)	3.70	2.96	0.74	12.59	6.67	5.19		
			61-70 (n=67)	1.49	2.99	4.48	8.96	1.49	2.99		
			71-80 (n=21)	0.00	0.00	0.00	14.29	4.76	0.00		
	SIDER	SIDER	SIDER							4.00	56.00
**Feelingsick**	SLAM	Trust	Clozapine (n=1760)	4.66	4.94	6.48	14.32	11.19	9.09		
		Age Groups	Under 21 (n=57)	7.02	17.54	15.79	31.58	26.32	19.30		
			21-30 (n=422)	7.82	6.40	7.11	20.38	15.64	12.80		
			31-40 (n=488)	3.89	5.53	6.76	12.91	10.86	7.58		
			41-50 (n=479)	3.55	2.92	6.05	11.48	9.81	8.56		
			51-60 (n=233)	2.15	2.58	4.72	9.44	5.58	4.72		
			61-70 (n=62)	3.23	3.23	1.61	11.29	4.84	6.45		
			71-80 (n=18)	11.11	5.56	5.56	5.56	0.00	11.11		
	Camden & Islington	Trust	Clozapine (n=561)	3.74	3.92	3.03	10.52	7.13	7.66		
		Age Groups	21-30 (n=27)	7.41	14.81	11.11	7.41	7.41	22.22		
			31-40 (n=141)	7.09	3.55	4.96	14.89	12.06	8.51		
			41-50 (n=168)	0.60	2.98	2.38	10.71	4.76	7.74		
			51-60 (n=135)	4.44	3.70	0.74	7.41	4.44	4.44		
			61-70 (n=67)	2.99	2.99	1.49	8.96	8.96	7.46		
			71-80 (n=21)	0.00	4.76	4.76	9.52	4.76	4.76		
	SIDER	SIDER	SIDER								
**Headache**	SLAM	Trust	Clozapine (n=1760)	4.20	4.55	5.45	12.44	8.18	5.91		
		Age Groups	Under 21 (n=57)	15.79	17.54	7.02	12.28	14.04	14.04		
			21-30 (n=422)	5.92	6.40	7.82	17.06	9.72	7.82		
			31-40 (n=488)	3.48	3.48	5.94	12.91	8.20	5.94		
			41-50 (n=479)	3.13	3.55	4.38	10.65	6.47	5.64		
			51-60 (n=233)	3.00	3.00	3.00	8.58	7.30	2.58		
			61-70 (n=62)	1.61	1.61	0.00	6.45	8.06	1.61		
			71-80 (n=18)	0.00	5.56	11.11	11.11	11.11	0.00		
	Camden & Islington	Trust	Clozapine (n=561)	2.32	3.57	4.28	9.27	6.42	4.63		
		Age Groups	21-30 (n=27)	3.70	22.22	3.70	11.11	11.11	0.00		
			31-40 (n=141)	4.26	4.96	5.67	12.77	9.93	9.93		
			41-50 (n=168)	1.79	1.19	4.17	7.74	4.76	4.17		
			51-60 (n=135)	0.74	0.74	3.70	7.41	2.96	0.74		
			61-70 (n=67)	2.99	5.97	4.48	10.45	10.45	5.97		
			71-80 (n=21)	0.00	0.00	0.00	4.76	0.00	0.00		
	SIDER	SIDER	SIDER								
**Insomnia**	SLAM	Trust	Clozapine (n=1760)	3.92	4.03	5.17	10.40	6.48	4.03		
		Age Groups	Under 21 (n=57)	12.28	8.77	10.53	21.05	7.02	10.53		
			21-30 (n=422)	5.69	4.50	5.92	11.85	7.82	5.69		
			31-40 (n=488)	4.10	4.92	4.51	10.25	6.35	3.07		
			41-50 (n=479)	1.88	3.55	6.05	9.39	7.52	4.38		
			51-60 (n=233)	3.00	2.15	3.86	9.01	3.43	2.15		
			61-70 (n=62)	3.23	0.00	0.00	6.45	1.61	0.00		
			71-80 (n=18)	0.00	5.56	0.00	5.56	5.56	0.00		
	Camden & Islington	Trust	Clozapine (n=561)	3.57	3.39	3.74	8.91	3.39	4.28		
		Age Groups	21-30 (n=27)	7.41	7.41	3.70	14.81	7.41	3.70		
			31-40 (n=141)	5.67	5.67	4.96	9.93	4.26	6.38		
			41-50 (n=168)	2.38	2.98	2.38	5.95	0.00	2.38		
			51-60 (n=135)	2.22	2.22	3.70	8.15	5.93	5.19		
			61-70 (n=67)	1.49	1.49	5.97	13.43	4.48	4.48		
			71-80 (n=21)	9.52	0.00	0.00	9.52	0.00	0.00		
	SIDER	SIDER	SIDER							20.00	33.00
**Hypertension**	SLAM	Trust	Clozapine (n=1760)	2.05	2.22	3.13	9.15	5.74	4.60		
		Age Groups	Under 21 (n=57)	0.00	1.75	0.00	14.04	10.53	5.26		
			21-30 (n=422)	1.66	1.18	1.66	7.58	4.27	3.79		
			31-40 (n=488)	1.23	1.64	2.25	6.15	4.30	3.48		
			41-50 (n=479)	2.92	2.92	4.59	11.27	5.85	4.80		
			51-60 (n=233)	2.15	2.15	3.86	11.16	6.87	6.44		

The results are shown in percentages (%) and broken down by ADRs, Trusts (SLAM, Camden & Islington and Oxford), Cohorts, Sub Cohorts and SIDER reported values.
In Sub Cohort 'Clozapine' represent the total baseline population which further breaks down into age groups.
The columns (Three Months Early, Two Months Early, One Month Early, One Month Later, Two Months Later, Three Months Later) shows the percentages in each monthly interval. The last two columns (SIDER Low End and SIDER High End) shows the SIDER reporting.

Clozapine - Age Groups (%)

Measure Values: 0.00 – 75.44

ADR	Trust	Cohort	Sub Cohort	Three Months Early	Two Months Early	One Month Early	One Month Later	Two Months Later	Three Months Later	SIDER Low End	SIDER High End
Hypertension	SLAM	Age Groups	61-70 (n=62)	6.45	4.84	6.45	12.90	17.74	9.68		
			71-80 (n=18)	0.00	11.11	11.11	11.11	0.00	5.56		
	Camden & Islington	Trust	Clozapine (n=561)	0.71	0.71	1.60	7.13	4.63	2.67		
		Age Groups	21-30 (n=27)	0.00	0.00	0.00	3.70	3.70	0.00		
			31-40 (n=141)	1.42	0.00	0.00	4.96	7.09	3.55		
			41-50 (n=168)	0.00	0.60	0.00	3.57	2.98	2.98		
			51-60 (n=135)	0.74	0.74	2.22	8.15	2.96	1.48		
			61-70 (n=67)	0.00	2.99	5.97	14.93	5.97	4.48		
			71-80 (n=21)	4.76	0.00	9.52	23.81	9.52	0.00		
	SIDER	SIDER	SIDER							4.00	12.00
Shaking	SLAM	Trust	Clozapine (n=1760)	3.13	2.95	3.92	9.55	5.40	5.06		
		Age Groups	Under 21 (n=57)	5.26	7.02	10.53	14.04	14.04	17.54		
			21-30 (n=422)	6.16	3.55	4.50	15.64	7.58	7.35		
			31-40 (n=488)	1.64	3.07	3.69	8.20	4.30	4.30		
			41-50 (n=479)	2.30	2.51	3.55	7.52	4.18	2.51		
			51-60 (n=233)	2.15	1.29	3.00	6.01	3.86	3.86		
			61-70 (n=62)	1.61	1.61	1.61	6.45	4.84	6.45		
			71-80 (n=18)	5.56	11.11	5.56	0.00	11.11	11.11		
	Camden & Islington	Trust	Clozapine (n=561)	1.78	1.96	3.74	6.06	3.92	2.85		
		Age Groups	21-30 (n=27)	0.00	7.41	14.81	22.22	11.11	14.81		
			31-40 (n=141)	4.26	2.84	4.96	5.67	4.26	4.96		
			41-50 (n=168)	0.60	1.79	2.98	4.17	2.38	0.60		
			51-60 (n=135)	1.48	0.74	1.48	5.93	2.96	0.00		
			61-70 (n=67)	1.49	1.49	1.49	2.99	4.48	5.97		
			71-80 (n=21)	0.00	0.00	9.52	14.29	9.52	0.00		
	SIDER	SIDER	SIDER								
Vomiting	SLAM	Trust	Clozapine (n=1760)	2.56	2.50	3.01	8.86	6.82	5.00		
		Age Groups	Under 21 (n=57)	5.26	7.02	12.28	17.54	15.79	17.54		
			21-30 (n=422)	3.55	3.08	4.27	12.80	9.00	6.16		
			31-40 (n=488)	2.66	3.69	2.46	7.38	7.17	4.30		
			41-50 (n=479)	1.88	1.46	2.09	7.10	5.01	3.76		
			51-60 (n=233)	2.15	0.43	2.15	6.87	4.72	3.86		
			61-70 (n=62)	0.00	0.00	1.61	6.45	3.23	4.84		
			71-80 (n=18)	0.00	5.56	0.00	11.11	5.56	5.56		
	Camden & Islington	Trust	Clozapine (n=561)	2.14	2.50	2.85	6.77	4.99	4.63		
		Age Groups	21-30 (n=27)	11.11	7.41	11.11	11.11	11.11	11.11		
			31-40 (n=141)	3.55	2.84	3.55	9.93	7.80	6.38		
			41-50 (n=168)	0.60	1.19	1.79	6.55	2.98	4.76		
			51-60 (n=135)	1.48	2.22	2.96	2.96	2.96	2.96		
			61-70 (n=67)	1.49	2.99	1.49	7.46	4.48	2.99		
			71-80 (n=21)	0.00	4.76	0.00	4.76	9.52	0.00		
	SIDER	SIDER	SIDER							3.00	17.00
Hyperprolactinaemia	SLAM	Trust	Clozapine (n=1760)	3.18	3.64	4.20	8.52	5.06	4.15		
		Age Groups	Under 21 (n=57)	12.28	14.04	8.77	21.05	17.54	12.28		
			21-30 (n=422)	3.55	4.50	5.45	9.95	6.40	7.11		
			31-40 (n=488)	3.89	4.10	4.30	8.81	4.92	3.89		
			41-50 (n=479)	1.88	2.09	3.34	8.14	3.97	3.13		
			51-60 (n=233)	1.72	2.58	3.00	4.29	3.43	0.86		
			61-70 (n=62)	3.23	1.61	3.23	6.45	1.61	0.00		
			71-80 (n=18)	0.00	0.00	0.00	0.00	0.00	0.00		
	Camden & Islington	Trust	Clozapine (n=561)	1.60	1.78	2.67	8.20	4.10	3.57		
		Age Groups	21-30 (n=27)	7.41	0.00	7.41	14.81	3.70	0.00		
			31-40 (n=141)	2.84	2.84	6.38	12.77	5.67	4.96		
			41-50 (n=168)	0.00	0.60	0.00	7.74	4.17	4.17		
			51-60 (n=135)	1.48	1.48	0.74	5.93	2.96	2.96		
			61-70 (n=67)	1.49	4.48	4.48	4.48	4.48	2.99		
			71-80 (n=21)	0.00	0.00	0.00	0.00	0.00	0.00		
	SIDER	SIDER	SIDER								
Tremor	SLAM	Trust	Clozapine (n=1760)	1.48	1.99	2.95	5.51	3.52	3.47		
		Age Groups	Under 21 (n=57)	1.75	3.51	3.51	12.28	7.02	7.02		
			21-30 (n=422)	0.95	2.37	3.32	5.69	2.13	3.55		
			31-40 (n=488)	1.23	0.61	1.84	4.10	2.46	3.48		
			41-50 (n=479)	1.25	2.09	2.51	4.59	4.18	2.51		
			51-60 (n=233)	2.15	2.15	5.15	6.87	5.15	3.00		
			61-70 (n=62)	3.23	4.84	3.23	9.68	4.84	8.06		
			71-80 (n=18)	11.11	11.11	5.56	11.11	11.11	5.56		

The results are shown in percentages (%) and broken down by ADRs, Trusts (SLAM, Camden & Islington and Oxford), Cohorts, Sub Cohorts and SIDER reported values.
In Sub Cohort 'Clozapine' represent the total baseline population which further breaks down into age groups.
The columns (Three Months Early, Two Months Early, One Month Early, One Month Later, Two Months Later, Three Months Later) shows the percentages in each monthly interval. The last two columns (SIDER Low End and SIDER High End) shows the SIDER reporting.

Clozapine - Age Groups (%)

Measure Values: 0.00 – 75.44

ADR	Trust	Cohort	Sub Cohort	Three Months Early	Two Months Early	One Month Early	One Month Later	Two Months Later	Three Months Later	SIDER Low End	SIDER High End
Tremor	Camden & Islington	Trust	Clozapine (n=561)	1.60	1.78	2.14	3.92	1.96	2.14		
		Age Groups	21-30 (n=27)	3.70	0.00	7.41	7.41	0.00	3.70		
			31-40 (n=141)	2.13	2.84	2.84	2.84	2.13	2.13		
			41-50 (n=168)	0.60	1.19	1.19	2.98	2.38	1.79		
			51-60 (n=135)	0.74	0.74	2.22	2.96	0.74	0.74		
			61-70 (n=67)	1.49	4.48	1.49	7.46	2.99	5.97		
			71-80 (n=21)	9.52	0.00	0.00	9.52	4.76	0.00		
	SIDER	SIDER	SIDER							6.00	
Abdominalpain	SLAM	Trust	Clozapine (n=1760)	1.88	1.99	2.56	8.01	6.02	4.72		
		Age Groups	Under 21 (n=57)	3.51	0.00	1.75	12.28	8.77	5.26		
			21-30 (n=422)	2.84	2.13	3.32	9.24	5.92	4.98		
			31-40 (n=488)	1.02	1.43	1.84	8.61	7.17	5.53		
			41-50 (n=479)	2.51	3.76	2.30	7.52	4.38	4.18		
			51-60 (n=233)	0.43	0.43	3.86	5.58	6.87	3.43		
			61-70 (n=62)	0.00	0.00	0.00	6.45	4.84	3.23		
			71-80 (n=18)	5.56	0.00	5.56	0.00	5.56	5.56		
	Camden & Islington	Trust	Clozapine (n=561)	0.89	0.89	1.60	3.92	3.57	3.39		
		Age Groups	21-30 (n=27)	3.70	0.00	3.70	7.41	3.70	3.70		
			31-40 (n=141)	0.00	1.42	2.84	7.09	4.96	4.96		
			41-50 (n=168)	0.00	0.60	1.19	2.98	3.57	2.98		
			51-60 (n=135)	1.48	0.74	0.74	1.48	1.48	0.74		
			61-70 (n=67)	0.00	1.49	1.49	4.48	4.48	4.48		
			71-80 (n=21)	0.00	0.00	0.00	0.00	4.76	9.52		
	SIDER	SIDER	SIDER							4.00	
Hypotension	SLAM	Trust	Clozapine (n=1760)	0.51	0.97	0.80	5.00	2.95	2.56		
		Age Groups	Under 21 (n=57)	1.75	3.51	3.51	15.79	8.77	12.28		
			21-30 (n=422)	0.47	0.95	0.95	4.98	2.84	3.79		
			31-40 (n=488)	0.20	0.41	0.41	4.10	2.87	1.64		
			41-50 (n=479)	0.21	1.25	0.42	3.76	1.67	0.84		
			51-60 (n=233)	0.86	0.86	1.29	5.58	3.00	3.00		
			61-70 (n=62)	1.61	0.00	0.00	9.68	9.68	4.84		
			71-80 (n=18)	5.56	5.56	5.56	5.56	0.00	0.00		
	Camden & Islington	Trust	Clozapine (n=561)	0.18	0.53	0.18	3.57	2.32	1.78		
		Age Groups	21-30 (n=27)	0.00	0.00	0.00	0.00	0.00	0.00		
			31-40 (n=141)	0.00	0.00	0.71	2.13	4.26	0.71		
			41-50 (n=168)	0.00	0.00	0.00	2.38	0.00	1.19		
			51-60 (n=135)	0.00	0.00	0.00	5.93	2.96	2.96		
			61-70 (n=67)	1.49	4.48	0.00	4.48	2.99	0.00		
			71-80 (n=21)	0.00	0.00	0.00	9.52	4.76	14.29		
	SIDER	SIDER	SIDER							9.00	38.00
Nausea	SLAM	Trust	Clozapine (n=1760)	1.14	1.08	1.19	6.08	5.23	3.69		
		Age Groups	Under 21 (n=57)	3.51	5.26	1.75	7.02	8.77	7.02		
			21-30 (n=422)	1.66	0.95	1.42	8.06	7.11	4.74		
			31-40 (n=488)	0.82	1.64	1.43	5.74	4.30	2.87		
			41-50 (n=479)	0.63	0.63	0.63	5.64	5.22	2.09		
			51-60 (n=233)	1.72	0.43	1.29	4.72	4.29	4.72		
			61-70 (n=62)	0.00	0.00	1.61	3.23	1.61	8.06		
			71-80 (n=18)	0.00	0.00	0.00	5.56	0.00	5.56		
	Camden & Islington	Trust	Clozapine (n=561)	0.89	1.43	0.36	4.63	3.57	3.57		
		Age Groups	21-30 (n=27)	0.00	3.70	0.00	3.70	7.41	3.70		
			31-40 (n=141)	1.42	1.42	0.00	4.26	4.26	2.84		
			41-50 (n=168)	0.00	0.60	1.19	4.76	1.19	4.17		
			51-60 (n=135)	1.48	1.48	0.00	4.44	4.44	3.70		
			61-70 (n=67)	1.49	1.49	0.00	5.97	5.97	2.99		
			71-80 (n=21)	0.00	4.76	0.00	4.76	0.00	9.52		
	SIDER	SIDER	SIDER							3.00	17.00
Fever	SLAM	Trust	Clozapine (n=1760)	1.02	1.14	1.65	6.36	4.43	3.13		
		Age Groups	Under 21 (n=57)	1.75	3.51	5.26	5.26	7.02	7.02		
			21-30 (n=422)	1.42	2.13	2.13	7.11	3.79	4.27		
			31-40 (n=488)	1.02	1.02	1.43	6.76	4.30	1.84		
			41-50 (n=479)	0.84	0.63	0.84	6.68	3.76	3.13		
			51-60 (n=233)	0.43	0.43	2.15	4.72	5.58	2.58		
			61-70 (n=62)	0.00	0.00	1.61	3.23	6.45	3.23		
			71-80 (n=18)	5.56	0.00	0.00	5.56	11.11	5.56		
	Camden & Islington	Trust	Clozapine (n=561)	0.89	0.89	0.53	3.74	2.67	0.89		
		Age Groups	21-30 (n=27)	0.00	3.70	3.70	7.41	7.41	7.41		

The results are shown in percentages (%) and broken down by ADRs, Trusts (SLAM, Camden & Islington and Oxford), Cohorts, Sub Cohorts and SIDER reported values.
In Sub Cohort 'Clozapine' represent the total baseline population which further breaks down into age groups.
The columns (Three Months Early, Two Months Early, One Month Early, One Month Later, Two Months Later, Three Months Later) shows the percentages in each monthly interval. The last two columns (SIDER Low End and SIDER High End) shows the SIDER reporting.

Clozapine - Age Groups (%)

Measure Values

0.00 75.44

ADR	Trust	Cohort	Sub Cohort	Three Months Early	Two Months Early	One Month Early	One Month Later	Two Months Later	Three Months Later	SIDER Low End	SIDER High End
Fever	Camden & Islington	Age Groups	31-40 (n=141)	1.42	2.13	0.71	4.96	4.26	1.42		
			41-50 (n=168)	0.00	0.00	0.00	0.00	1.19	0.60		
			51-60 (n=135)	1.48	0.74	0.00	5.19	1.48	0.00		
			61-70 (n=67)	1.49	0.00	1.49	5.97	2.99	0.00		
			71-80 (n=21)	0.00	0.00	0.00	4.76	4.76	0.00		
	SIDER	SIDER	SIDER							4.00	13.00
Convulsion	SLAM	Trust	Clozapine (n=1760)	1.36	1.70	1.82	7.05	4.94	4.03		
		Age Groups	Under 21 (n=57)	0.00	1.75	3.51	10.53	5.26	8.77		
			21-30 (n=422)	2.37	2.37	1.66	9.95	6.87	4.50		
			31-40 (n=488)	1.02	1.64	1.64	6.56	3.48	4.30		
			41-50 (n=479)	1.46	2.09	2.09	6.26	3.97	3.13		
			51-60 (n=233)	0.86	0.00	2.15	4.72	4.72	2.15		
			61-70 (n=62)	0.00	0.00	0.00	4.84	9.68	8.06		
			71-80 (n=18)	0.00	5.56	0.00	0.00	5.56	5.56		
	Camden & Islington	Trust	Clozapine (n=561)	0.53	0.53	0.36	2.85	2.14	1.07		
		Age Groups	21-30 (n=27)	3.70	3.70	3.70	3.70	0.00	0.00		
			31-40 (n=141)	0.71	1.42	0.71	2.13	3.55	2.13		
			41-50 (n=168)	0.60	0.00	0.00	0.60	2.38	0.60		
			51-60 (n=135)	0.00	0.00	0.00	4.44	2.22	1.48		
			61-70 (n=67)	0.00	0.00	0.00	4.48	0.00	0.00		
			71-80 (n=21)	0.00	0.00	0.00	9.52	0.00	0.00		
	SIDER	SIDER	SIDER							3.00	
Enuresis	SLAM	Trust	Clozapine (n=1760)	1.02	0.80	1.25	4.20	3.92	3.24		
		Age Groups	Under 21 (n=57)	1.75	0.00	0.00	3.51	7.02	8.77		
			21-30 (n=422)	1.42	0.71	0.95	3.79	3.55	2.37		
			31-40 (n=488)	1.23	0.61	0.41	2.66	4.10	4.10		
			41-50 (n=479)	0.84	1.04	2.71	4.38	3.76	2.30		
			51-60 (n=233)	0.43	0.86	0.43	6.87	3.43	3.00		
			61-70 (n=62)	0.00	1.61	0.00	8.06	6.45	4.84		
			71-80 (n=18)	0.00	0.00	11.11	5.56	0.00	0.00		
	Camden & Islington	Trust	Clozapine (n=561)	0.71	1.07	1.07	4.10	1.43	1.25		
		Age Groups	21-30 (n=27)	0.00	0.00	0.00	3.70	0.00	0.00		
			31-40 (n=141)	0.00	2.13	1.42	4.96	2.84	1.42		
			41-50 (n=168)	1.19	0.60	0.60	1.79	0.00	0.60		
			51-60 (n=135)	1.48	0.74	1.48	4.44	0.74	0.74		
			61-70 (n=67)	0.00	1.49	1.49	4.48	1.49	1.49		
			71-80 (n=21)	0.00	0.00	0.00	14.29	9.52	9.52		
	SIDER	SIDER	SIDER								
Backache	SLAM	Trust	Clozapine (n=1760)	1.14	1.59	2.44	4.94	3.35	2.73		
		Age Groups	Under 21 (n=57)	0.00	1.75	3.51	5.26	3.51	1.75		
			21-30 (n=422)	0.95	0.95	1.90	3.79	3.32	3.08		
			31-40 (n=488)	0.61	1.84	2.66	3.69	2.66	2.66		
			41-50 (n=479)	1.67	1.88	2.51	6.26	3.34	2.92		
			51-60 (n=233)	1.29	0.86	2.58	6.01	4.29	1.29		
			61-70 (n=62)	1.61	4.84	1.61	8.06	4.84	4.84		
			71-80 (n=18)	5.56	0.00	5.56	0.00	5.56	5.56		
	Camden & Islington	Trust	Clozapine (n=561)	1.43	1.25	1.96	5.35	3.03	3.03		
		Age Groups	21-30 (n=27)	0.00	3.70	0.00	0.00	0.00	3.70		
			31-40 (n=141)	1.42	1.42	2.13	4.26	2.13	4.26		
			41-50 (n=168)	1.19	0.00	1.79	3.57	2.98	2.98		
			51-60 (n=135)	0.74	0.74	2.22	5.93	1.48	1.48		
			61-70 (n=67)	4.48	2.99	2.99	14.93	8.96	2.99		
			71-80 (n=21)	0.00	4.76	0.00	0.00	4.76	4.76		
	SIDER	SIDER	SIDER							5.00	
Stomachpain	SLAM	Trust	Clozapine (n=1760)	1.93	1.76	1.93	4.94	3.52	3.52		
		Age Groups	Under 21 (n=57)	3.51	5.26	5.26	7.02	3.51	5.26		
			21-30 (n=422)	2.84	2.61	2.61	4.98	3.55	4.27		
			31-40 (n=488)	2.25	1.23	1.64	5.94	3.69	3.69		
			41-50 (n=479)	1.46	1.67	1.46	4.18	4.38	3.34		
			51-60 (n=233)	0.43	0.86	0.86	3.00	2.15	1.29		
			61-70 (n=62)	0.00	0.00	3.23	8.06	0.00	6.45		
			71-80 (n=18)	5.56	5.56	5.56	5.56	5.56	0.00		
	Camden & Islington	Trust	Clozapine (n=561)	0.89	1.25	0.89	3.39	2.85	2.14		
		Age Groups	21-30 (n=27)	7.41	0.00	0.00	3.70	7.41	3.70		
			31-40 (n=141)	0.71	2.13	0.71	3.55	5.67	2.84		
			41-50 (n=168)	0.00	0.00	0.60	4.76	2.38	2.98		

The results are shown in percentages (%) and broken down by ADRs, Trusts (SLAM, Camden & Islington and Oxford), Cohorts, Sub Cohorts and SIDER reported values.
In Sub Cohort 'Clozapine' represent the total baseline population which further breaks down into age groups.
The columns (Three Months Early, Two Months Early, One Month Early, One Month Later, Two Months Later, Three Months Later) shows the percentages in each monthly interval. The last two columns (SIDER Low End and SIDER High End) shows the SIDER reporting.

Clozapine - Age Groups (%)

Measure Values: 0.00 – 75.44

ADR	Trust	Cohort	Sub Cohort	Three Months Early	Two Months Early	One Month Early	One Month Later	Two Months Later	Three Months Later	SIDER Low End	SIDER High End
**Stomachpain**	Camden & Islington	Age Groups	51-60 (n=135)	1.48	2.22	1.48	2.22	0.74	0.74		
			61-70 (n=67)	0.00	1.49	1.49	1.49	1.49	0.00		
			71-80 (n=21)	0.00	0.00	0.00	4.76	0.00	4.76		
	SIDER	SIDER	SIDER								
**Diarrhoea**	SLAM	Trust	Clozapine (n=1760)	1.08	1.31	1.36	4.72	3.58	2.56		
		Age Groups	Under 21 (n=57)	3.51	5.26	0.00	7.02	3.51	5.26		
			21-30 (n=422)	0.47	0.71	1.18	4.74	2.84	2.84		
			31-40 (n=488)	1.02	1.23	1.43	3.89	3.89	2.87		
			41-50 (n=479)	1.25	1.04	1.67	4.80	3.76	2.09		
			51-60 (n=233)	0.86	2.15	1.29	5.15	3.43	1.29		
			61-70 (n=62)	1.61	0.00	1.61	6.45	4.84	4.84		
			71-80 (n=18)	5.56	5.56	0.00	5.56	5.56	0.00		
	Camden & Islington	Trust	Clozapine (n=561)	0.71	1.25	0.18	3.03	3.39	3.03		
		Age Groups	21-30 (n=27)	0.00	3.70	3.70	3.70	7.41	11.11		
			31-40 (n=141)	2.13	0.71	0.00	4.26	4.96	2.13		
			41-50 (n=168)	0.00	0.60	0.00	1.79	2.38	2.38		
			51-60 (n=135)	0.74	1.48	0.00	2.96	2.22	2.96		
			61-70 (n=67)	0.00	1.49	0.00	2.99	2.99	2.99		
			71-80 (n=21)	0.00	4.76	0.00	4.76	4.76	4.76		
	SIDER	SIDER	SIDER							2.00	
**Drymouth**	SLAM	Trust	Clozapine (n=1760)	1.08	1.53	1.65	4.66	3.69	2.33		
		Age Groups	Under 21 (n=57)	1.75	3.51	5.26	10.53	5.26	7.02		
			21-30 (n=422)	1.90	1.18	1.90	5.69	5.69	2.84		
			31-40 (n=488)	0.20	1.23	1.43	4.92	1.84	2.46		
			41-50 (n=479)	0.63	1.67	1.67	3.76	2.92	1.88		
			51-60 (n=233)	1.72	2.15	0.86	3.43	4.72	0.86		
			61-70 (n=62)	3.23	0.00	1.61	3.23	6.45	3.23		
			71-80 (n=18)	0.00	5.56	0.00	0.00	0.00	0.00		
	Camden & Islington	Trust	Clozapine (n=561)	1.25	1.25	1.07	3.92	2.14	0.89		
		Age Groups	21-30 (n=27)	0.00	0.00	0.00	7.41	7.41	0.00		
			31-40 (n=141)	1.42	2.13	2.13	4.96	3.55	0.71		
			41-50 (n=168)	1.19	1.19	1.79	4.17	1.19	1.19		
			51-60 (n=135)	0.74	0.00	0.00	2.22	2.22	1.48		
			61-70 (n=67)	2.99	2.99	0.00	4.48	0.00	0.00		
			71-80 (n=21)	0.00	0.00	0.00	0.00	0.00	0.00		
	SIDER	SIDER	SIDER							5.00	20.00
**Rash**	SLAM	Trust	Clozapine (n=1760)	1.25	1.59	2.05	3.64	2.95	2.27		
		Age Groups	Under 21 (n=57)	0.00	5.26	7.02	5.26	3.51	1.75		
			21-30 (n=422)	0.95	1.18	2.37	4.27	3.32	2.84		
			31-40 (n=488)	1.23	1.43	1.84	3.89	3.07	2.25		
			41-50 (n=479)	1.67	1.46	1.67	2.71	3.13	2.09		
			51-60 (n=233)	0.86	1.72	1.29	3.43	2.15	2.58		
			61-70 (n=62)	1.61	1.61	3.23	3.23	1.61	0.00		
			71-80 (n=18)	5.56	5.56	0.00	5.56	0.00	0.00		
	Camden & Islington	Trust	Clozapine (n=561)	1.25	1.25	0.89	4.28	1.96	2.14		
		Age Groups	21-30 (n=27)	7.41	7.41	3.70	0.00	3.70	7.41		
			31-40 (n=141)	0.00	0.71	1.42	4.26	1.42	2.84		
			41-50 (n=168)	1.79	1.79	0.60	3.57	2.38	1.19		
			51-60 (n=135)	1.48	0.74	0.00	3.70	1.48	0.74		
			61-70 (n=67)	0.00	0.00	1.49	8.96	2.99	2.99		
			71-80 (n=21)	0.00	0.00	0.00	4.76	0.00	4.76		
	SIDER	SIDER	SIDER								
**Neutropenia**	SLAM	Trust	Clozapine (n=1760)	0.80	0.80	0.74	5.34	2.73	2.61		
		Age Groups	Under 21 (n=57)	3.51	5.26	1.75	12.28	5.26	3.51		
			21-30 (n=422)	1.66	1.42	1.66	8.77	4.27	4.03		
			31-40 (n=488)	0.61	0.82	0.41	3.28	1.84	1.84		
			41-50 (n=479)	0.21	0.21	0.42	3.97	2.51	2.30		
			51-60 (n=233)	0.00	0.00	0.43	5.58	1.72	2.58		
			61-70 (n=62)	1.61	0.00	0.00	3.23	3.23	1.61		
			71-80 (n=18)	0.00	0.00	0.00	0.00	0.00	0.00		
	Camden & Islington	Trust	Clozapine (n=561)	0.00	0.18	0.53	1.60	0.89	1.07		
		Age Groups	21-30 (n=27)	0.00	0.00	0.00	3.70	0.00	0.00		
			31-40 (n=141)	0.00	0.71	0.71	0.71	1.42	2.13		
			41-50 (n=168)	0.00	0.00	0.60	2.38	0.60	0.60		
			51-60 (n=135)	0.00	0.00	0.74	1.48	1.48	0.74		
			61-70 (n=67)	0.00	0.00	0.00	1.49	0.00	1.49		

The results are shown in percentages (%) and broken down by ADRs, Trusts (SLAM, Camden & Islington and Oxford), Cohorts, Sub Cohorts and SIDER reported values.
In Sub Cohort 'Clozapine' represent the total baseline population which further breaks down into age groups.
The columns (Three Months Early, Two Months Early, One Month Early, One Month Later, Two Months Later, Three Months Later) shows the percentages in each monthly interval. The last two columns (SIDER Low End and SIDER High End) shows the SIDER reporting.

Clozapine - Age Groups (%)

ADR	Trust	Cohort	Sub Cohort	Three Months Early	Two Months Early	One Month Early	One Month Later	Two Months Later	Three Months Later	SIDER Low End	SIDER High End
Neutropenia	Islington	Age Groups	71-80 (n=21)	0.00	0.00	0.00	0.00	0.00	0.00		
	SIDER	SIDER	SIDER								
Sweating	SLAM	Trust	Clozapine (n=1760)	1.08	0.97	1.36	4.43	4.26	2.84		
		Age Groups	Under 21 (n=57)	3.51	5.26	3.51	10.53	8.77	7.02		
			21-30 (n=422)	0.71	0.95	2.13	3.55	4.27	2.61		
			31-40 (n=488)	1.02	0.61	1.43	4.51	4.51	3.07		
			41-50 (n=479)	1.46	1.04	1.04	5.22	3.55	2.92		
			51-60 (n=233)	0.43	0.43	0.00	3.86	5.15	2.15		
			61-70 (n=62)	0.00	0.00	0.00	1.61	1.61	1.61		
			71-80 (n=18)	5.56	0.00	0.00	0.00	0.00	0.00		
	Camden & Islington	Trust	Clozapine (n=561)	0.53	0.53	0.53	2.85	2.14	1.96		
		Age Groups	21-30 (n=27)	7.41	0.00	0.00	0.00	7.41	0.00		
			31-40 (n=141)	0.00	0.00	0.71	2.13	1.42	2.13		
			41-50 (n=168)	0.00	1.19	0.00	4.17	1.79	1.19		
			51-60 (n=135)	0.74	0.74	1.48	1.48	2.96	2.22		
			61-70 (n=67)	0.00	0.00	0.00	5.97	1.49	4.48		
			71-80 (n=21)	0.00	0.00	0.00	0.00	0.00	0.00		
	SIDER	SIDER	SIDER							6.00	
Dyspepsia	SLAM	Trust	Clozapine (n=1760)	0.74	1.08	0.91	3.92	3.13	3.69		
		Age Groups	Under 21 (n=57)	0.00	1.75	0.00	5.26	1.75	1.75		
			21-30 (n=422)	0.95	0.95	1.42	5.45	3.55	4.03		
			31-40 (n=488)	1.02	1.43	1.02	4.10	3.48	4.71		
			41-50 (n=479)	0.63	0.84	0.42	3.76	3.13	3.13		
			51-60 (n=233)	0.00	0.86	0.43	2.15	2.15	2.15		
			61-70 (n=62)	1.61	0.00	1.61	0.00	3.23	4.84		
			71-80 (n=18)	0.00	5.56	5.56	0.00	0.00	5.56		
	Camden & Islington	Trust	Clozapine (n=561)	0.36	0.53	0.53	4.10	2.67	2.50		
		Age Groups	21-30 (n=27)	0.00	0.00	0.00	3.70	7.41	3.70		
			31-40 (n=141)	0.71	2.13	0.71	4.26	1.42	3.55		
			41-50 (n=168)	0.00	0.00	1.19	6.55	4.76	3.57		
			51-60 (n=135)	0.00	0.00	0.00	2.96	0.00	0.00		
			61-70 (n=67)	1.49	0.00	0.00	1.49	4.48	2.99		
			71-80 (n=21)	0.00	0.00	0.00	0.00	0.00	0.00		
	SIDER	SIDER	SIDER							8.00	14.00
Blurredvision	SLAM	Trust	Clozapine (n=1760)	0.34	0.91	0.63	2.05	1.25	1.02		
		Age Groups	Under 21 (n=57)	0.00	0.00	0.00	3.51	1.75	1.75		
			21-30 (n=422)	0.71	1.18	0.47	2.37	1.90	1.42		
			31-40 (n=488)	0.41	1.64	0.82	3.07	1.43	1.02		
			41-50 (n=479)	0.21	0.21	0.63	1.46	0.42	0.42		
			51-60 (n=233)	0.00	0.43	0.43	0.43	0.86	0.43		
			61-70 (n=62)	0.00	0.00	1.61	1.61	3.23	3.23		
			71-80 (n=18)	0.00	5.56	0.00	0.00	0.00	5.56		
	Camden & Islington	Trust	Clozapine (n=561)	0.89	0.53	0.71	1.25	0.36	0.89		
		Age Groups	21-30 (n=27)	0.00	0.00	0.00	3.70	0.00	0.00		
			31-40 (n=141)	0.71	0.71	1.42	0.00	1.42	0.00		
			41-50 (n=168)	0.00	0.00	0.00	1.79	0.00	0.60		
			51-60 (n=135)	1.48	0.74	0.00	2.22	0.00	1.48		
			61-70 (n=67)	2.99	1.49	2.99	0.00	0.00	2.99		
			71-80 (n=21)	0.00	0.00	0.00	0.00	0.00	0.00		
	SIDER	SIDER	SIDER							5.00	
Akathisia	SLAM	Trust	Clozapine (n=1760)	0.80	0.91	0.74	2.67	1.36	0.80		
		Age Groups	Under 21 (n=57)	0.00	1.75	0.00	1.75	5.26	0.00		
			21-30 (n=422)	0.71	0.95	1.18	4.74	1.90	0.95		
			31-40 (n=488)	1.02	0.82	0.61	2.05	0.61	0.41		
			41-50 (n=479)	0.84	1.25	1.04	2.30	0.84	0.84		
			51-60 (n=233)	0.86	0.43	0.00	2.15	2.15	1.29		
			61-70 (n=62)	0.00	0.00	0.00	0.00	1.61	1.61		
			71-80 (n=18)	0.00	0.00	0.00	0.00	0.00	0.00		
	Camden & Islington	Trust	Clozapine (n=561)	0.00	0.53	0.00	1.25	1.07	0.53		
		Age Groups	21-30 (n=27)	0.00	0.00	0.00	0.00	0.00	0.00		
			31-40 (n=141)	0.00	0.71	0.00	0.71	1.42	0.71		
			41-50 (n=168)	0.00	1.19	0.00	2.38	1.19	1.19		
			51-60 (n=135)	0.00	0.00	0.00	0.74	0.00	0.00		
			61-70 (n=67)	0.00	0.00	0.00	1.49	2.99	0.00		
			71-80 (n=21)	0.00	0.00	0.00	0.00	0.00	0.00		
	SIDER	SIDER	SIDER							3.00	

Measure Values
0.00 75.44

The results are shown in percentages (%) and broken down by ADRs, Trusts (SLAM, Camden & Islington and Oxford), Cohorts, Sub Cohorts and SIDER reported values.
In Sub Cohort 'Clozapine' represent the total baseline population which further breaks down into age groups.
The columns (Three Months Early, Two Months Early, One Month Early, One Month Later, Two Months Later, Three Months Later) shows the percentages in each monthly interval. The last two columns (SIDER Low End and SIDER High End) shows the SIDER reporting.

Clozapine - Hospital Admission (%)

Measure Values: 0.00 – 79.82

ADR	Trust	Cohort	Sub Cohort	Three Months Early	Two Months Early	One Month Early	One Month Later	Two Months Later	Three Months Later	SIDER Low End	SIDER High End
Agitation	SLAM	Trust	Clozapine (n=1760)	17.61	22.10	26.53	46.59	32.56	26.99		
		Hospital Admission	Inpatient (n=737)	30.66	40.43	52.65	77.61	55.36	45.59		
			Outpatient (n=1023)	8.21	8.90	7.72	24.24	16.13	13.59		
	Camden & Islington	Trust	Clozapine (n=561)	13.37	17.83	18.36	43.14	28.34	21.03		
		Hospital Admission	Inpatient (n=114)	11.40	16.67	21.93	79.82	51.75	32.46		
			Outpatient (n=447)	13.87	18.12	17.45	33.78	22.37	18.12		
	SIDER	SIDER	SIDER							4.00	
Fatigue	SLAM	Trust	Clozapine (n=1760)	12.67	14.83	15.85	43.58	35.80	30.51		
		Hospital Admission	Inpatient (n=737)	21.71	26.32	30.26	68.66	59.16	50.88		
			Outpatient (n=1023)	6.16	6.55	5.47	25.51	18.96	15.84		
	Camden & Islington	Trust	Clozapine (n=561)	10.34	12.30	13.37	41.18	29.23	26.56		
		Hospital Admission	Inpatient (n=114)	12.28	6.14	11.40	64.04	51.75	43.86		
			Outpatient (n=447)	9.84	13.87	13.87	35.35	23.49	22.15		
	SIDER	SIDER	SIDER								
Sedation	SLAM	Trust	Clozapine (n=1760)	12.67	12.16	14.83	43.86	35.51	29.83		
		Hospital Admission	Inpatient (n=737)	21.71	22.93	29.58	70.69	56.17	49.12		
			Outpatient (n=1023)	6.16	4.40	4.20	24.54	20.63	15.93		
	Camden & Islington	Trust	Clozapine (n=561)	5.17	9.09	9.09	38.15	26.56	21.93		
		Hospital Admission	Inpatient (n=114)	2.63	6.14	9.65	65.79	48.25	30.70		
			Outpatient (n=447)	5.82	9.84	8.95	31.10	21.03	19.69		
	SIDER	SIDER	SIDER							25.00	46.00
Dizziness	SLAM	Trust	Clozapine (n=1760)	2.78	4.20	4.09	16.59	13.13	11.19		
		Hospital Admission	Inpatient (n=737)	4.61	7.87	8.55	28.36	20.35	16.55		
			Outpatient (n=1023)	1.47	1.56	0.88	8.11	7.92	7.33		
	Camden & Islington	Trust	Clozapine (n=561)	3.21	3.39	3.74	18.18	13.73	9.09		
		Hospital Admission	Inpatient (n=114)	2.63	1.75	4.39	33.33	29.82	19.30		
			Outpatient (n=447)	3.36	3.80	3.58	14.32	9.62	6.49		
	SIDER	SIDER	SIDER							12.00	27.00
Confusion	SLAM	Trust	Clozapine (n=1760)	4.72	5.51	6.08	13.92	8.47	6.76		
		Hospital Admission	Inpatient (n=737)	8.28	10.58	13.16	24.15	14.79	11.26		
			Outpatient (n=1023)	2.15	1.86	0.98	6.55	3.91	3.52		
	Camden & Islington	Trust	Clozapine (n=561)	3.57	6.24	5.53	12.66	6.77	5.88		
		Hospital Admission	Inpatient (n=114)	3.51	5.26	4.39	27.19	15.79	7.02		
			Outpatient (n=447)	3.58	6.49	5.82	8.95	4.47	5.59		
	SIDER	SIDER	SIDER							3.00	
Tachycardia	SLAM	Trust	Clozapine (n=1760)	2.27	2.05	2.50	15.40	12.95	9.94		
		Hospital Admission	Inpatient (n=737)	4.34	4.34	5.29	28.22	23.34	16.96		
			Outpatient (n=1023)	0.78	0.39	0.49	6.16	5.47	4.89		
	Camden & Islington	Trust	Clozapine (n=561)	1.43	1.43	0.89	11.23	8.38	6.95		
		Hospital Admission	Inpatient (n=114)	1.75	1.75	0.88	23.68	16.67	11.40		
			Outpatient (n=447)	1.34	1.34	0.89	8.05	6.26	5.82		
	SIDER	SIDER	SIDER							11.00	25.00
Hypersalivation	SLAM	Trust	Clozapine (n=1760)	1.19	1.48	2.10	14.32	13.24	11.31		
		Hospital Admission	Inpatient (n=737)	1.36	2.17	3.66	21.98	21.57	17.23		
			Outpatient (n=1023)	1.08	0.98	0.98	8.80	7.23	7.04		
	Camden & Islington	Trust	Clozapine (n=561)	1.07	1.43	0.53	14.26	6.95	7.66		
		Hospital Admission	Inpatient (n=114)	0.00	1.75	0.00	15.79	11.40	9.65		
			Outpatient (n=447)	1.34	1.34	0.67	13.87	5.82	7.16		
	SIDER	SIDER	SIDER							1.00	48.00
Weightgain	SLAM	Trust	Clozapine (n=1760)	3.75	4.43	5.06	15.34	10.91	10.34		
		Hospital Admission	Inpatient (n=737)	6.65	7.87	10.04	24.15	18.32	18.05		
			Outpatient (n=1023)	1.66	1.96	1.47	8.99	5.57	4.79		
	Camden & Islington	Trust	Clozapine (n=561)	2.50	3.39	1.96	11.76	6.60	6.24		
		Hospital Admission	Inpatient (n=114)	3.51	4.39	1.75	16.67	6.14	10.53		
			Outpatient (n=447)	2.24	3.13	2.01	10.51	6.71	5.15		
	SIDER	SIDER	SIDER							4.00	56.00
Feelingsick	SLAM	Trust	Clozapine (n=1760)	4.66	4.94	6.48	14.32	11.19	9.09		
		Hospital Admission	Inpatient (n=737)	8.55	9.23	13.43	24.02	18.59	14.79		
			Outpatient (n=1023)	1.86	1.86	1.47	7.33	5.87	4.99		
	Camden & Islington	Trust	Clozapine (n=561)	3.74	3.92	3.03	10.52	7.13	7.66		
		Hospital Admission	Inpatient (n=114)	4.39	0.88	1.75	21.05	10.53	11.40		
			Outpatient (n=447)	3.58	4.70	3.36	7.83	6.26	6.71		
	SIDER	SIDER	SIDER								
Constipation	SLAM	Trust	Clozapine (n=1760)	1.76	1.99	2.16	12.27	11.70	9.49		
		Hospital Admission	Inpatient (n=737)	3.12	3.39	4.07	18.86	18.72	13.16		
			Outpatient (n=1023)	0.78	0.98	0.78	7.53	6.65	6.84		
	Camden &	Trust	Clozapine (n=561)	1.07	2.50	1.78	11.41	7.13	5.70		

The results are shown in percentages (%) and broken down by ADRs, Trusts (SLAM, Camden & Islington and Oxford), Cohorts, Sub Cohorts and SIDER reported values.
In Sub Cohort 'Clozapine' represent the total baseline population which further breaks down into 'Inpatients' and 'Outpatients'.
The columns (Three Months Early, Two Months Early, One Month Early, One Month Later, Two Months Later, Three Months Later) shows the percentages in each monthly interval. The last two columns (SIDER Low End and SIDER High End) shows the SIDER reporting.

Clozapine - Hospital Admission (%)

Measure Values 0.00 – 79.82

ADR	Trust	Cohort	Sub Cohort	Three Months Early	Two Months Early	One Month Early	One Month Later	Two Months Later	Three Months Later	SIDER Low End	SIDER High End
Constipation	Camden & Islington	Hospital Admission	Inpatient (n=114)	0.00	2.63	2.63	22.81	12.28	7.89		
			Outpatient (n=447)	1.34	2.46	1.57	8.50	5.82	5.15		
	SIDER	SIDER	SIDER							10.00	25.00
Headache	SLAM	Trust	Clozapine (n=1760)	4.20	4.55	5.45	12.44	8.18	5.91		
		Hospital Admission	Inpatient (n=737)	6.51	8.01	10.18	20.90	11.94	9.77		
			Outpatient (n=1023)	2.54	2.05	2.05	6.35	5.47	3.13		
	Camden & Islington	Trust	Clozapine (n=561)	2.32	3.57	4.28	9.27	6.42	4.63		
		Hospital Admission	Inpatient (n=114)	0.00	2.63	5.26	17.54	10.53	8.77		
			Outpatient (n=447)	2.91	3.80	4.03	7.16	5.37	3.58		
	SIDER	SIDER	SIDER								
Insomnia	SLAM	Trust	Clozapine (n=1760)	3.92	4.03	5.17	10.40	6.48	4.03		
		Hospital Admission	Inpatient (n=737)	5.70	7.46	9.50	17.50	9.77	5.43		
			Outpatient (n=1023)	2.64	1.56	2.05	5.28	4.11	3.03		
	Camden & Islington	Trust	Clozapine (n=561)	3.57	3.39	3.74	8.91	3.39	4.28		
		Hospital Admission	Inpatient (n=114)	1.75	4.39	7.89	21.05	7.02	5.26		
			Outpatient (n=447)	4.03	3.13	2.68	5.82	2.46	4.03		
	SIDER	SIDER	SIDER							20.00	33.00
Hyperprolactinaemia	SLAM	Trust	Clozapine (n=1760)	3.18	3.64	4.20	8.52	5.06	4.15		
		Hospital Admission	Inpatient (n=737)	5.02	6.38	7.73	13.84	8.41	6.65		
			Outpatient (n=1023)	1.86	1.66	1.66	4.69	2.64	2.35		
	Camden & Islington	Trust	Clozapine (n=561)	1.60	1.78	2.67	8.20	4.10	3.57		
		Hospital Admission	Inpatient (n=114)	2.63	1.75	1.75	15.79	5.26	3.51		
			Outpatient (n=447)	1.34	1.79	2.91	6.26	3.80	3.58		
	SIDER	SIDER	SIDER								
Hypertension	SLAM	Trust	Clozapine (n=1760)	2.05	2.22	3.13	9.15	5.74	4.60		
		Hospital Admission	Inpatient (n=737)	3.53	4.21	5.97	13.03	8.96	7.19		
			Outpatient (n=1023)	0.98	0.78	1.08	6.35	3.42	2.74		
	Camden & Islington	Trust	Clozapine (n=561)	0.71	0.71	1.60	7.13	4.63	2.67		
		Hospital Admission	Inpatient (n=114)	0.00	0.00	2.63	13.16	7.89	4.39		
			Outpatient (n=447)	0.89	0.89	1.34	5.59	3.80	2.24		
	SIDER	SIDER	SIDER							4.00	12.00
Vomiting	SLAM	Trust	Clozapine (n=1760)	2.56	2.50	3.01	8.86	6.82	5.00		
		Hospital Admission	Inpatient (n=737)	4.21	4.61	5.97	12.62	11.26	8.68		
			Outpatient (n=1023)	1.37	0.98	0.88	6.16	3.62	2.35		
	Camden & Islington	Trust	Clozapine (n=561)	2.14	2.50	2.85	6.77	4.99	4.63		
		Hospital Admission	Inpatient (n=114)	1.75	0.88	2.63	11.40	6.14	6.14		
			Outpatient (n=447)	2.24	2.91	2.91	5.59	4.70	4.25		
	SIDER	SIDER	SIDER							3.00	17.00
Shaking	SLAM	Trust	Clozapine (n=1760)	3.13	2.95	3.92	9.55	5.40	5.06		
		Hospital Admission	Inpatient (n=737)	6.11	5.43	8.14	16.82	9.77	8.68		
			Outpatient (n=1023)	0.98	1.17	0.88	4.30	2.25	2.44		
	Camden & Islington	Trust	Clozapine (n=561)	1.78	1.96	3.74	6.06	3.92	2.85		
		Hospital Admission	Inpatient (n=114)	0.00	0.88	3.51	7.02	5.26	6.14		
			Outpatient (n=447)	2.24	2.24	3.80	5.82	3.58	2.01		
	SIDER	SIDER	SIDER								
Abdominalpain	SLAM	Trust	Clozapine (n=1760)	1.88	1.99	2.56	8.01	6.02	4.72		
		Hospital Admission	Inpatient (n=737)	3.66	3.93	5.29	14.38	8.82	7.73		
			Outpatient (n=1023)	0.59	0.59	0.59	3.42	4.01	2.54		
	Camden & Islington	Trust	Clozapine (n=561)	0.89	0.89	1.60	3.92	3.57	3.39		
		Hospital Admission	Inpatient (n=114)	0.00	0.88	2.63	6.14	9.65	4.39		
			Outpatient (n=447)	0.67	0.89	1.34	3.36	2.01	3.13		
	SIDER	SIDER	SIDER							4.00	
Fever	SLAM	Trust	Clozapine (n=1760)	1.02	1.14	1.65	6.36	4.43	3.13		
		Hospital Admission	Inpatient (n=737)	2.04	2.44	3.26	11.13	6.51	5.70		
			Outpatient (n=1023)	0.29	0.20	0.49	2.93	2.93	1.27		
	Camden & Islington	Trust	Clozapine (n=561)	0.89	0.89	0.53	3.74	2.67	0.89		
		Hospital Admission	Inpatient (n=114)	0.88	0.00	0.00	11.40	4.39	0.88		
			Outpatient (n=447)	0.89	1.12	0.67	1.79	2.24	0.89		
	SIDER	SIDER	SIDER							4.00	13.00
Backache	SLAM	Trust	Clozapine (n=1760)	1.14	1.59	2.44	4.94	3.35	2.73		
		Hospital Admission	Inpatient (n=737)	2.31	3.26	5.16	9.09	6.24	4.48		
			Outpatient (n=1023)	0.29	0.39	0.49	1.96	1.27	1.47		
	Camden & Islington	Trust	Clozapine (n=561)	1.43	1.25	1.96	5.35	3.03	3.03		
		Hospital Admission	Inpatient (n=114)	2.63	1.75	2.63	12.28	8.77	4.39		
			Outpatient (n=447)	1.12	1.12	1.79	3.58	1.57	2.68		
	SIDER	SIDER	SIDER							5.00	
Nausea	SLAM	Trust	Clozapine (n=1760)	1.14	1.08	1.19	6.08	5.23	3.69		

The results are shown in percentages (%) and broken down by ADRs, Trusts (SLAM, Camden & Islington and Oxford), Cohorts, Sub Cohorts and SIDER reported values.
In Sub Cohort 'Clozapine' represent the total baseline population which further breaks down into 'Inpatients' and 'Outpatients'.
The columns (Three Months Early, Two Months Early, One Month Early, One Month Later, Two Months Later, Three Months Later) shows the percentages in each monthly interval. The last two columns (SIDER Low End and SIDER High End) shows the SIDER reporting.

Clozapine - Hospital Admission (%)

Measure Values: 0.00 – 79.82

ADR	Trust	Cohort	Sub Cohort	Three Months Early	Two Months Early	One Month Early	One Month Later	Two Months Later	Three Months Later	SIDER Low End	SIDER High End
Nausea	SLAM	Hospital Admission	Inpatient (n=737)	1.90	1.90	2.17	9.50	7.73	5.02		
			Outpatient (n=1023)	0.59	0.49	0.49	3.62	3.42	2.74		
	Camden & Islington	Trust	Clozapine (n=561)	0.89	1.43	0.36	4.63	3.57	3.57		
		Hospital Admission	Inpatient (n=114)	0.88	0.88	0.00	9.65	5.26	2.63		
			Outpatient (n=447)	0.89	1.57	0.45	3.36	3.13	3.80		
	SIDER	SIDER	SIDER							3.00	17.00
Tremor	SLAM	Trust	Clozapine (n=1760)	1.48	1.99	2.95	5.51	3.52	3.47		
		Hospital Admission	Inpatient (n=737)	2.44	3.80	6.11	9.36	6.38	6.24		
			Outpatient (n=1023)	0.78	0.68	0.68	2.74	1.47	1.47		
	Camden & Islington	Trust	Clozapine (n=561)	1.60	1.78	2.14	3.92	1.96	2.14		
		Hospital Admission	Inpatient (n=114)	0.88	1.75	2.63	8.77	3.51	4.39		
			Outpatient (n=447)	1.79	1.79	2.01	2.68	1.57	1.57		
	SIDER	SIDER	SIDER							6.00	
Convulsion	SLAM	Trust	Clozapine (n=1760)	1.36	1.70	1.82	7.05	4.94	4.03		
		Hospital Admission	Inpatient (n=737)	2.71	3.39	3.80	12.62	8.14	7.19		
			Outpatient (n=1023)	0.39	0.49	0.39	3.03	2.64	1.76		
	Camden & Islington	Trust	Clozapine (n=561)	0.53	0.53	0.36	2.85	2.14	1.07		
		Hospital Admission	Inpatient (n=114)	0.00	0.00	0.00	3.51	4.39	0.88		
			Outpatient (n=447)	0.67	0.67	0.45	2.68	1.57	1.12		
	SIDER	SIDER	SIDER							3.00	
Hypotension	SLAM	Trust	Clozapine (n=1760)	0.51	0.97	0.80	5.00	2.95	2.56		
		Hospital Admission	Inpatient (n=737)	0.81	1.76	1.90	9.77	5.16	3.93		
			Outpatient (n=1023)	0.29	0.39	0.00	1.56	1.37	1.56		
	Camden & Islington	Trust	Clozapine (n=561)	0.18	0.53	0.18	3.57	2.32	1.78		
		Hospital Admission	Inpatient (n=114)	0.00	0.88	0.00	6.14	6.14	1.75		
			Outpatient (n=447)	0.22	0.45	0.22	2.91	1.34	1.79		
	SIDER	SIDER	SIDER							9.00	38.00
Enuresis	SLAM	Trust	Clozapine (n=1760)	1.02	0.80	1.25	4.20	3.92	3.24		
		Hospital Admission	Inpatient (n=737)	1.76	1.22	2.58	8.14	6.78	5.43		
			Outpatient (n=1023)	0.49	0.49	0.29	1.37	1.86	1.66		
	Camden & Islington	Trust	Clozapine (n=561)	0.71	1.07	1.07	4.10	1.43	1.25		
		Hospital Admission	Inpatient (n=114)	0.88	0.88	3.51	7.89	2.63	3.51		
			Outpatient (n=447)	0.67	1.12	0.45	3.13	1.12	0.67		
	SIDER	SIDER	SIDER								
Drymouth	SLAM	Trust	Clozapine (n=1760)	1.08	1.53	1.65	4.66	3.69	2.33		
		Hospital Admission	Inpatient (n=737)	1.76	2.71	3.12	7.46	5.97	3.53		
			Outpatient (n=1023)	0.59	0.68	0.59	2.64	2.05	1.47		
	Camden & Islington	Trust	Clozapine (n=561)	1.25	1.25	1.07	3.92	2.14	0.89		
		Hospital Admission	Inpatient (n=114)	0.88	0.00	0.88	7.02	3.51	1.75		
			Outpatient (n=447)	1.34	1.57	1.12	3.13	1.79	0.67		
	SIDER	SIDER	SIDER							5.00	20.00
Stomachpain	SLAM	Trust	Clozapine (n=1760)	1.93	1.76	1.93	4.94	3.52	3.52		
		Hospital Admission	Inpatient (n=737)	3.53	3.53	4.07	8.68	6.11	5.56		
			Outpatient (n=1023)	0.78	0.49	0.39	2.25	1.66	2.05		
	Camden & Islington	Trust	Clozapine (n=561)	0.89	1.25	0.89	3.39	2.85	2.14		
		Hospital Admission	Inpatient (n=114)	0.00	1.75	0.88	7.02	6.14	2.63		
			Outpatient (n=447)	1.12	1.12	0.89	2.46	2.01	2.01		
	SIDER	SIDER	SIDER								
Dyspepsia	SLAM	Trust	Clozapine (n=1760)	0.74	1.08	0.91	3.92	3.13	3.69		
		Hospital Admission	Inpatient (n=737)	1.22	2.04	2.04	7.06	4.34	6.38		
			Outpatient (n=1023)	0.39	0.39	0.10	1.66	2.25	1.76		
	Camden & Islington	Trust	Clozapine (n=561)	0.36	0.53	0.53	4.10	2.67	2.50		
		Hospital Admission	Inpatient (n=114)	0.00	0.88	0.88	7.89	4.39	7.02		
			Outpatient (n=447)	0.45	0.45	0.45	3.13	2.24	1.34		
	SIDER	SIDER	SIDER							8.00	14.00
Diarrhoea	SLAM	Trust	Clozapine (n=1760)	1.08	1.31	1.36	4.72	3.58	2.56		
		Hospital Admission	Inpatient (n=737)	2.17	2.58	2.85	8.41	5.97	4.75		
			Outpatient (n=1023)	0.29	0.39	0.29	2.05	1.86	0.98		
	Camden & Islington	Trust	Clozapine (n=561)	0.71	1.25	0.18	3.03	3.39	3.03		
		Hospital Admission	Inpatient (n=114)	1.75	1.75	0.00	6.14	6.14	1.75		
			Outpatient (n=447)	0.45	1.12	0.22	2.24	2.68	3.36		
	SIDER	SIDER	SIDER							2.00	
Rash	SLAM	Trust	Clozapine (n=1760)	1.25	1.59	2.05	3.64	2.95	2.27		
		Hospital Admission	Inpatient (n=737)	2.71	3.53	4.48	6.24	5.43	4.88		
			Outpatient (n=1023)	0.20	0.20	0.29	1.76	1.17	0.39		
	Camden & Islington	Trust	Clozapine (n=561)	1.25	1.25	0.89	4.28	1.96	2.14		
		Hospital	Inpatient (n=114)	0.00	1.75	1.75	7.02	3.51	2.63		

The results are shown in percentages (%) and broken down by ADRs, Trusts (SLAM, Camden & Islington and Oxford), Cohorts, Sub Cohorts and SIDER reported values.
In Sub Cohort 'Clozapine' represent the total baseline population which further breaks down into 'Inpatients' and 'Outpatients'.
The columns (Three Months Early, Two Months Early, One Month Early, One Month Later, Two Months Later, Three Months Later) shows the percentages in each monthly interval. The last two columns (SIDER Low End and SIDER High End) shows the SIDER reporting.

Clozapine - Hospital Admission (%)

ADR	Trust	Cohort	Sub Cohort	Three Months Early	Two Months Early	One Month Early	One Month Later	Two Months Later	Three Months Later	SIDER Low End	SIDER High End
Rash	Islington	Admission	Outpatient (n=447)	1.57	1.12	0.67	3.58	1.57	2.01		
	SIDER	SIDER	SIDER								
Sweating	SLAM	Trust	Clozapine (n=1760)	1.08	0.97	1.36	4.43	4.26	2.84		
		Hospital Admission	Inpatient (n=737)	1.90	2.04	2.85	7.87	5.97	4.61		
			Outpatient (n=1023)	0.49	0.20	0.29	1.96	3.03	1.56		
	Camden & Islington	Trust	Clozapine (n=561)	0.53	0.53	0.53	2.85	2.14	1.96		
		Hospital Admission	Inpatient (n=114)	0.00	0.00	0.88	6.14	4.39	4.39		
			Outpatient (n=447)	0.67	0.67	0.45	2.01	1.57	1.34		
	SIDER	SIDER	SIDER							6.00	
Neutropenia	SLAM	Trust	Clozapine (n=1760)	0.80	0.80	0.74	5.34	2.73	2.61		
		Hospital Admission	Inpatient (n=737)	1.09	1.63	1.63	8.68	4.07	4.34		
			Outpatient (n=1023)	0.59	0.20	0.10	2.93	1.76	1.37		
	Camden & Islington	Trust	Clozapine (n=561)	0.00	0.18	0.53	1.60	0.89	1.07		
		Hospital Admission	Inpatient (n=114)	0.00	0.00	0.00	2.63	0.00	0.88		
			Outpatient (n=447)	0.00	0.22	0.67	1.34	1.12	1.12		
	SIDER	SIDER	SIDER								
Akathisia	SLAM	Trust	Clozapine (n=1760)	0.80	0.91	0.74	2.67	1.36	0.80		
		Hospital Admission	Inpatient (n=737)	1.09	1.49	1.36	4.75	2.58	1.36		
			Outpatient (n=1023)	0.59	0.49	0.29	1.17	0.49	0.39		
	Camden & Islington	Trust	Clozapine (n=561)	0.00	0.53	0.00	1.25	1.07	0.53		
		Hospital Admission	Inpatient (n=114)	0.00	0.00	0.00	0.88	4.39	1.75		
			Outpatient (n=447)	0.00	0.67	0.00	1.34	0.22	0.22		
	SIDER	SIDER	SIDER							3.00	
Blurredvision	SLAM	Trust	Clozapine (n=1760)	0.34	0.91	0.63	2.05	1.25	1.02		
		Hospital Admission	Inpatient (n=737)	0.41	1.22	1.22	3.39	2.31	1.49		
			Outpatient (n=1023)	0.29	0.68	0.20	1.08	0.49	0.68		
	Camden & Islington	Trust	Clozapine (n=561)	0.89	0.53	0.71	1.25	0.36	0.89		
		Hospital Admission	Inpatient (n=114)	0.00	0.00	1.75	2.63	0.88	0.00		
			Outpatient (n=447)	1.12	0.67	0.45	0.89	0.22	1.12		
	SIDER	SIDER	SIDER							5.00	

Measure Values
0.00 79.82

The results are shown in percentages (%) and broken down by ADRs, Trusts (SLAM, Camden & Islington and Oxford), Cohorts, Sub Cohorts and SIDER reported values.
In Sub Cohort 'Clozapine' represent the total baseline population which further breaks down into 'Inpatients' and 'Outpatients'.
The columns (Three Months Early, Two Months Early, One Month Early, One Month Later, Two Months Later, Three Months Later) shows the percentages in each monthly interval. The last two columns (SIDER Low End and SIDER High End) shows the SIDER reporting.

Clozapine - Smoking Status (%)

Measure Values: 0.00 – 63.04

ADR	Trust	Cohort	Sub Cohort	Three Months Early	Two Months Early	One Month Early	One Month Later	Two Months Later	Three Months Later	SIDER Low End	SIDER High End
Agitation	SLAM	Trust	Clozapine (n=1760)	17.61	22.10	26.53	46.59	32.56	26.99		
		Smoking Status	Smoker (n=1039)	24.35	30.13	36.67	63.04	44.47	38.98		
			Non Smoker (n=721)	7.91	10.54	11.93	22.88	15.40	9.71		
	Camden & Islington	Trust	Clozapine (n=561)	13.37	17.83	18.36	43.14	28.34	21.03		
		Smoking Status	Smoker (n=360)	0.83	24.72	25.56	56.94	37.78	28.33		
			Non Smoker (n=201)	1.49	5.47	5.47	18.41	11.44	7.96		
Fatigue	SLAM	Trust	Clozapine (n=1760)	12.67	14.83	15.85	43.58	35.80	30.51		
		Smoking Status	Smoker (n=1039)	16.65	19.54	21.37	56.98	47.16	41.67		
			Non Smoker (n=721)	6.93	8.04	7.91	24.27	19.42	14.42		
	Camden & Islington	Trust	Clozapine (n=561)	10.34	12.30	13.37	41.18	29.23	26.56		
		Smoking Status	Smoker (n=360)	0.28	16.67	17.78	51.94	39.17	35.83		
			Non Smoker (n=201)	0.50	4.48	5.47	21.89	11.44	9.95		
Sedation	SLAM	Trust	Clozapine (n=1760)	12.67	12.16	14.83	43.86	35.51	29.83		
		Smoking Status	Smoker (n=1039)	16.84	16.46	20.50	56.98	46.29	39.36		
			Non Smoker (n=721)	6.66	5.96	6.66	24.97	19.97	16.09		
	Camden & Islington	Trust	Clozapine (n=561)	5.17	9.09	9.09	38.15	26.56	21.93		
		Smoking Status	Smoker (n=360)	0.00	11.67	12.78	45.56	34.17	28.33		
			Non Smoker (n=201)	0.00	4.48	2.49	24.88	12.94	10.45		
Dizziness	SLAM	Trust	Clozapine (n=1760)	2.78	4.20	4.09	16.59	13.13	11.19		
		Smoking Status	Smoker (n=1039)	3.66	5.87	5.49	20.98	16.55	14.63		
			Non Smoker (n=721)	1.53	1.80	2.08	10.26	8.18	6.24		
	Camden & Islington	Trust	Clozapine (n=561)	3.21	3.39	3.74	18.18	13.73	9.09		
		Smoking Status	Smoker (n=360)	0.28	4.44	5.00	23.33	18.06	11.94		
			Non Smoker (n=201)	0.50	1.49	1.49	8.96	5.97	3.98		
Hypersalivation	SLAM	Trust	Clozapine (n=1760)	1.19	1.48	2.10	14.32	13.24	11.31		
		Smoking Status	Smoker (n=1039)	1.06	1.92	2.50	16.84	15.50	13.47		
			Non Smoker (n=721)	1.39	0.83	1.53	10.68	9.99	8.18		
	Camden & Islington	Trust	Clozapine (n=561)	1.07	1.43	0.53	14.26	6.95	7.66		
		Smoking Status	Smoker (n=360)	0.00	1.67	0.56	15.83	8.33	9.72		
			Non Smoker (n=201)	0.00	1.00	0.50	11.44	4.48	3.98		
Weightgain	SLAM	Trust	Clozapine (n=1760)	3.75	4.43	5.06	15.34	10.91	10.34		
		Smoking Status	Smoker (n=1039)	4.72	5.58	6.26	18.96	14.73	13.09		
			Non Smoker (n=721)	2.36	2.77	3.33	10.12	5.41	6.38		
	Camden & Islington	Trust	Clozapine (n=561)	2.50	3.39	1.96	11.76	6.60	6.24		
		Smoking Status	Smoker (n=360)	0.00	4.72	2.78	13.89	8.89	8.61		
			Non Smoker (n=201)	0.00	1.00	0.50	7.96	2.49	1.99		
Tachycardia	SLAM	Trust	Clozapine (n=1760)	2.27	2.05	2.50	15.40	12.95	9.94		
		Smoking Status	Smoker (n=1039)	2.89	2.60	3.08	20.40	17.32	12.90		
			Non Smoker (n=721)	1.39	1.25	1.66	8.18	6.66	5.69		
	Camden & Islington	Trust	Clozapine (n=561)	1.43	1.43	0.89	11.23	8.38	6.95		
		Smoking Status	Smoker (n=360)	0.00	1.39	1.39	14.72	10.00	8.61		
			Non Smoker (n=201)	0.00	1.49	0.00	4.98	5.47	3.98		
Confusion	SLAM	Trust	Clozapine (n=1760)	4.72	5.51	6.08	13.92	8.47	6.76		
		Smoking Status	Smoker (n=1039)	6.16	7.70	8.85	19.25	11.16	9.72		
			Non Smoker (n=721)	2.64	2.36	2.08	6.24	4.58	2.50		
	Camden & Islington	Trust	Clozapine (n=561)	3.57	6.24	5.53	12.66	6.77	5.88		
		Smoking Status	Smoker (n=360)	0.28	9.44	8.06	17.22	8.89	8.61		
			Non Smoker (n=201)	0.50	0.50	1.00	4.48	2.99	1.00		
Feelingsick	SLAM	Trust	Clozapine (n=1760)	4.66	4.94	6.48	14.32	11.19	9.09		
		Smoking Status	Smoker (n=1039)	6.06	6.64	8.37	18.29	14.73	11.65		
			Non Smoker (n=721)	2.64	2.50	3.74	8.60	6.10	5.41		
	Camden & Islington	Trust	Clozapine (n=561)	3.74	3.92	3.03	10.52	7.13	7.66		
		Smoking Status	Smoker (n=360)	0.00	5.56	4.72	14.44	9.72	10.56		
			Non Smoker (n=201)	0.00	1.00	0.00	3.48	2.49	2.49		
Constipation	SLAM	Trust	Clozapine (n=1760)	1.76	1.99	2.16	12.27	11.70	9.49		
		Smoking Status	Smoker (n=1039)	2.21	2.50	2.79	14.63	15.11	11.26		
			Non Smoker (n=721)	1.11	1.25	1.25	8.88	6.80	6.93		
	Camden & Islington	Trust	Clozapine (n=561)	1.07	2.50	1.78	11.41	7.13	5.70		
		Smoking Status	Smoker (n=360)	0.28	3.61	2.22	13.61	8.61	7.50		
			Non Smoker (n=201)	0.50	0.50	1.00	7.46	4.48	2.49		
Headache	SLAM	Trust	Clozapine (n=1760)	4.20	4.55	5.45	12.44	8.18	5.91		
		Smoking Status	Smoker (n=1039)	5.58	5.87	7.12	17.04	11.16	8.95		
			Non Smoker (n=721)	2.22	2.64	3.05	5.83	3.88	1.53		
	Camden & Islington	Trust	Clozapine (n=561)	2.32	3.57	4.28	9.27	6.42	4.63		
		Smoking Status	Smoker (n=360)	0.00	5.28	6.11	12.22	8.06	6.11		
			Non Smoker (n=201)	0.00	0.50	1.00	3.98	3.48	1.99		
Insomnia	SLAM	Trust	Clozapine (n=1760)	3.92	4.03	5.17	10.40	6.48	4.03		

The results are shown in percentages (%) and broken down by ADRs, Trusts (SLAM, Camden & Islington and Oxford), Cohorts, Sub Cohorts and SIDER reported values.
In Sub Cohort 'Clozapine' represent the total baseline population which further breaks down into 'Smokers and 'Non-Smokers'.
The columns (Three Months Early, Two Months Early, One Month Early, One Month Later, Two Months Later, Three Months Later) shows the percentages in each monthly interval. The last two columns (SIDER Low End and SIDER High End) shows the SIDER reporting.

Clozapine - Smoking Status (%)

ADR	Trust	Cohort	Sub Cohort	Three Months Early	Two Months Early	One Month Early	One Month Later	Two Months Later	Three Months Later	SIDER Low End	SIDER High End
Insomnia	SLAM	Smoking Status	Smoker (n=1039)	4.72	5.29	6.35	14.15	8.47	5.29		
			Non Smoker (n=721)	2.77	2.22	3.47	4.99	3.61	2.22		
	Camden & Islington	Trust	Clozapine (n=561)	3.57	3.39	3.74	8.91	3.39	4.28		
		Smoking Status	Smoker (n=360)	0.56	4.44	5.28	12.22	4.72	5.83		
			Non Smoker (n=201)	1.00	1.49	1.00	2.99	1.00	1.49		
Hyperprolactinaemia	SLAM	Trust	Clozapine (n=1760)	3.18	3.64	4.20	8.52	5.06	4.15		
		Smoking Status	Smoker (n=1039)	3.46	4.04	4.81	9.14	5.39	4.72		
			Non Smoker (n=721)	2.77	3.05	3.33	7.63	4.58	3.33		
	Camden & Islington	Trust	Clozapine (n=561)	1.60	1.78	2.67	8.20	4.10	3.57		
		Smoking Status	Smoker (n=360)	0.00	2.22	3.06	10.28	4.72	3.06		
			Non Smoker (n=201)	0.00	1.00	1.99	4.48	2.99	4.48		
Hypertension	SLAM	Trust	Clozapine (n=1760)	2.05	2.22	3.13	9.15	5.74	4.60		
		Smoking Status	Smoker (n=1039)	2.41	2.79	4.23	11.16	7.41	6.35		
			Non Smoker (n=721)	1.53	1.39	1.53	6.24	3.33	2.08		
	Camden & Islington	Trust	Clozapine (n=561)	0.71	0.71	1.60	7.13	4.63	2.67		
		Smoking Status	Smoker (n=360)	0.28	0.56	1.94	7.78	6.11	3.06		
			Non Smoker (n=201)	0.50	1.00	1.00	5.97	1.99	1.99		
Shaking	SLAM	Trust	Clozapine (n=1760)	3.13	2.95	3.92	9.55	5.40	5.06		
		Smoking Status	Smoker (n=1039)	3.27	3.27	5.10	12.51	7.41	7.41		
			Non Smoker (n=721)	2.91	2.50	2.22	5.27	2.50	1.66		
	Camden & Islington	Trust	Clozapine (n=561)	1.78	1.96	3.74	6.06	3.92	2.85		
		Smoking Status	Smoker (n=360)	0.00	2.78	4.72	7.50	4.72	3.89		
			Non Smoker (n=201)	0.00	0.50	1.99	3.48	2.49	1.00		
Vomiting	SLAM	Trust	Clozapine (n=1760)	2.56	2.50	3.01	8.86	6.82	5.00		
		Smoking Status	Smoker (n=1039)	3.18	3.27	3.95	11.16	9.14	6.35		
			Non Smoker (n=721)	1.66	1.39	1.66	5.55	3.47	3.05		
	Camden & Islington	Trust	Clozapine (n=561)	2.14	2.50	2.85	6.77	4.99	4.63		
		Smoking Status	Smoker (n=360)	0.00	3.06	3.33	9.44	6.39	5.83		
			Non Smoker (n=201)	0.00	1.49	1.99	1.99	2.49	2.49		
Abdominalpain	SLAM	Trust	Clozapine (n=1760)	1.88	1.99	2.56	8.01	6.02	4.72		
		Smoking Status	Smoker (n=1039)	2.31	2.60	3.66	10.78	8.18	6.93		
			Non Smoker (n=721)	1.25	1.11	0.97	4.02	2.91	1.53		
	Camden & Islington	Trust	Clozapine (n=561)	0.89	0.89	1.60	3.92	3.57	3.39		
		Smoking Status	Smoker (n=360)	0.00	1.39	2.22	4.72	4.72	4.44		
			Non Smoker (n=201)	0.00	0.00	0.50	2.49	1.49	1.49		
Nausea	SLAM	Trust	Clozapine (n=1760)	1.14	1.08	1.19	6.08	5.23	3.69		
		Smoking Status	Smoker (n=1039)	1.15	0.96	1.64	7.51	5.97	4.62		
			Non Smoker (n=721)	1.11	1.25	0.55	4.02	4.16	2.36		
	Camden & Islington	Trust	Clozapine (n=561)	0.89	1.43	0.36	4.63	3.57	3.57		
		Smoking Status	Smoker (n=360)	0.00	1.94	0.00	5.83	4.44	3.61		
			Non Smoker (n=201)	0.00	0.50	1.00	2.49	1.99	3.48		
Backache	SLAM	Trust	Clozapine (n=1760)	1.14	1.59	2.44	4.94	3.35	2.73		
		Smoking Status	Smoker (n=1039)	1.64	2.21	3.46	6.64	4.91	3.46		
			Non Smoker (n=721)	0.42	0.69	0.97	2.50	1.11	1.66		
	Camden & Islington	Trust	Clozapine (n=561)	1.43	1.25	1.96	5.35	3.03	3.03		
		Smoking Status	Smoker (n=360)	0.00	1.94	3.06	7.50	3.89	4.17		
			Non Smoker (n=201)	0.00	0.00	0.00	1.49	1.49	1.00		
Convulsion	SLAM	Trust	Clozapine (n=1760)	1.36	1.70	1.82	7.05	4.94	4.03		
		Smoking Status	Smoker (n=1039)	1.83	2.12	2.41	9.53	6.35	4.72		
			Non Smoker (n=721)	0.69	1.11	0.97	3.47	2.91	3.05		
	Camden & Islington	Trust	Clozapine (n=561)	0.53	0.53	0.36	2.85	2.14	1.07		
		Smoking Status	Smoker (n=360)	0.00	0.83	0.56	3.33	3.06	1.39		
			Non Smoker (n=201)	0.00	0.00	0.00	1.99	0.50	0.50		
Fever	SLAM	Trust	Clozapine (n=1760)	1.02	1.14	1.65	6.36	4.43	3.13		
		Smoking Status	Smoker (n=1039)	1.25	1.54	2.21	8.47	6.16	4.43		
			Non Smoker (n=721)	0.69	0.55	0.83	3.33	1.94	1.25		
	Camden & Islington	Trust	Clozapine (n=561)	0.89	0.89	0.53	3.74	2.67	0.89		
		Smoking Status	Smoker (n=360)	0.00	1.11	0.83	5.28	3.89	1.11		
			Non Smoker (n=201)	0.00	0.50	0.00	1.00	0.50	0.50		
Tremor	SLAM	Trust	Clozapine (n=1760)	1.48	1.99	2.95	5.51	3.52	3.47		
		Smoking Status	Smoker (n=1039)	1.44	2.21	3.66	6.54	4.43	4.62		
			Non Smoker (n=721)	1.53	1.66	1.94	4.02	2.22	1.80		
	Camden & Islington	Trust	Clozapine (n=561)	1.60	1.78	2.14	3.92	1.96	2.14		
		Smoking Status	Smoker (n=360)	0.56	2.50	3.06	4.72	2.22	3.33		
			Non Smoker (n=201)	1.00	0.50	0.50	2.49	1.49	0.00		
Hypotension	SLAM	Trust	Clozapine (n=1760)	0.51	0.97	0.80	5.00	2.95	2.56		
		Smoking	Smoker (n=1039)	0.67	1.15	1.06	6.16	3.85	3.08		

Measure Values: 0.00 – 63.04

The results are shown in percentages (%) and broken down by ADRs, Trusts (SLAM, Camden & Islington and Oxford), Cohorts, Sub Cohorts and SIDER reported values.
In Sub Cohort 'Clozapine' represent the total baseline population which further breaks down into 'Smokers and 'Non-Smokers'.
The columns (Three Months Early, Two Months Early, One Month Early, One Month Later, Two Months Later, Three Months Later) shows the percentages in each monthly interval. The last two columns (SIDER Low End and SIDER High End) shows the SIDER reporting.

Clozapine - Smoking Status (%)

Measure Values: 0.00 – 63.04

ADR	Trust	Cohort	Sub Cohort	Three Months Early	Two Months Early	One Month Early	One Month Later	Two Months Later	Three Months Later	SIDER Low End	SIDER High End
Hypotension	SLAM	Status	Non Smoker (n=721)	0.28	0.69	0.42	3.33	1.66	1.80		
	Camden & Islington	Trust	Clozapine (n=561)	0.18	0.53	0.18	3.57	2.32	1.78		
		Smoking Status	Smoker (n=360)	0.00	0.83	0.28	4.17	2.50	2.22		
			Non Smoker (n=201)	0.00	0.00	0.00	2.49	1.99	1.00		
Drymouth	SLAM	Trust	Clozapine (n=1760)	1.08	1.53	1.65	4.66	3.69	2.33		
		Smoking Status	Smoker (n=1039)	1.06	1.92	2.21	5.77	4.72	3.56		
			Non Smoker (n=721)	1.11	0.97	0.83	3.05	2.22	0.55		
	Camden & Islington	Trust	Clozapine (n=561)	1.25	1.25	1.07	3.92	2.14	0.89		
		Smoking Status	Smoker (n=360)	0.00	1.11	1.67	5.00	2.78	0.83		
			Non Smoker (n=201)	0.00	1.49	0.00	1.99	1.00	1.00		
Enuresis	SLAM	Trust	Clozapine (n=1760)	1.02	0.80	1.25	4.20	3.92	3.24		
		Smoking Status	Smoker (n=1039)	1.15	1.15	1.83	6.26	5.10	4.14		
			Non Smoker (n=721)	0.83	0.28	0.42	1.25	2.22	1.94		
	Camden & Islington	Trust	Clozapine (n=561)	0.71	1.07	1.07	4.10	1.43	1.25		
		Smoking Status	Smoker (n=360)	0.00	1.39	1.11	5.28	1.39	1.39		
			Non Smoker (n=201)	0.00	0.50	1.00	1.99	1.49	1.00		
Stomachpain	SLAM	Trust	Clozapine (n=1760)	1.93	1.76	1.93	4.94	3.52	3.52		
		Smoking Status	Smoker (n=1039)	2.60	2.12	2.69	7.31	5.00	4.52		
			Non Smoker (n=721)	0.97	1.25	0.83	1.53	1.39	2.08		
	Camden & Islington	Trust	Clozapine (n=561)	0.89	1.25	0.89	3.39	2.85	2.14		
		Smoking Status	Smoker (n=360)	0.00	1.94	1.39	5.00	3.89	3.06		
			Non Smoker (n=201)	0.00	0.00	0.00	0.50	1.00	0.50		
Rash	SLAM	Trust	Clozapine (n=1760)	1.25	1.59	2.05	3.64	2.95	2.27		
		Smoking Status	Smoker (n=1039)	1.92	2.41	2.60	4.52	4.14	3.27		
			Non Smoker (n=721)	0.28	0.42	1.25	2.36	1.25	0.83		
	Camden & Islington	Trust	Clozapine (n=561)	1.25	1.25	0.89	4.28	1.96	2.14		
		Smoking Status	Smoker (n=360)	0.00	1.94	1.39	5.83	3.06	2.78		
			Non Smoker (n=201)	0.00	0.00	0.00	1.49	0.00	1.00		
Diarrhoea	SLAM	Trust	Clozapine (n=1760)	1.08	1.31	1.36	4.72	3.58	2.56		
		Smoking Status	Smoker (n=1039)	1.15	1.35	1.83	5.49	4.81	3.18		
			Non Smoker (n=721)	0.97	1.25	0.69	3.61	1.80	1.66		
	Camden & Islington	Trust	Clozapine (n=561)	0.71	1.25	0.18	3.03	3.39	3.03		
		Smoking Status	Smoker (n=360)	0.00	1.67	0.00	4.17	5.00	2.78		
			Non Smoker (n=201)	0.00	0.50	0.50	1.00	0.50	3.48		
Dyspepsia	SLAM	Trust	Clozapine (n=1760)	0.74	1.08	0.91	3.92	3.13	3.69		
		Smoking Status	Smoker (n=1039)	0.87	1.15	1.06	5.77	4.43	5.20		
			Non Smoker (n=721)	0.55	0.97	0.69	1.25	1.25	1.53		
	Camden & Islington	Trust	Clozapine (n=561)	0.36	0.53	0.53	4.10	2.67	2.50		
		Smoking Status	Smoker (n=360)	0.00	0.83	0.83	5.83	3.33	3.61		
			Non Smoker (n=201)	0.00	0.00	0.00	1.00	1.49	0.50		
Sweating	SLAM	Trust	Clozapine (n=1760)	1.08	0.97	1.36	4.43	4.26	2.84		
		Smoking Status	Smoker (n=1039)	1.06	1.25	1.83	5.97	5.97	4.33		
			Non Smoker (n=721)	1.11	0.55	0.69	2.22	1.80	0.69		
	Camden & Islington	Trust	Clozapine (n=561)	0.53	0.53	0.53	2.85	2.14	1.96		
		Smoking Status	Smoker (n=360)	0.00	0.83	0.56	3.89	2.78	2.50		
			Non Smoker (n=201)	0.00	0.00	0.50	1.00	1.00	1.00		
Neutropenia	SLAM	Trust	Clozapine (n=1760)	0.80	0.80	0.74	5.34	2.73	2.61		
		Smoking Status	Smoker (n=1039)	0.87	0.87	0.77	6.45	3.66	3.46		
			Non Smoker (n=721)	0.69	0.69	0.69	3.74	1.39	1.39		
	Camden & Islington	Trust	Clozapine (n=561)	0.00	0.18	0.53	1.60	0.89	1.07		
		Smoking Status	Smoker (n=360)	0.00	0.00	0.56	2.22	1.11	1.11		
			Non Smoker (n=201)	0.00	0.50	0.50	0.50	0.50	1.00		
Akathisia	SLAM	Trust	Clozapine (n=1760)	0.80	0.91	0.74	2.67	1.36	0.80		
		Smoking Status	Smoker (n=1039)	1.15	0.96	0.87	3.37	1.64	1.35		
			Non Smoker (n=721)	0.28	0.83	0.55	1.66	0.97	0.00		
	Camden & Islington	Trust	Clozapine (n=561)	0.00	0.53	0.00	1.25	1.07	0.53		
		Smoking Status	Smoker (n=360)	0.00	0.83	0.00	1.94	1.11	0.56		
			Non Smoker (n=201)	0.00	0.00	0.00	0.00	1.00	0.50		
Blurredvision	SLAM	Trust	Clozapine (n=1760)	0.34	0.91	0.63	2.05	1.25	1.02		
		Smoking Status	Smoker (n=1039)	0.19	1.35	0.96	2.21	1.73	1.44		
			Non Smoker (n=721)	0.55	0.28	0.14	1.80	0.55	0.42		
	Camden & Islington	Trust	Clozapine (n=561)	0.89	0.53	0.71	1.25	0.36	0.89		
		Smoking Status	Smoker (n=360)	0.00	0.83	1.11	1.67	0.56	1.11		
			Non Smoker (n=201)	0.00	0.00	0.00	0.50	0.00	0.50		

The results are shown in percentages (%) and broken down by ADRs, Trusts (SLAM, Camden & Islington and Oxford), Cohorts, Sub Cohorts and SIDER reported values.
In Sub Cohort 'Clozapine' represent the total baseline population which further breaks down into 'Smokers and 'Non-Smokers'.
The columns (Three Months Early, Two Months Early, One Month Early, One Month Later, Two Months Later, Three Months Later) shows the percentages in each monthly interval. The last two columns (SIDER Low End and SIDER High End) shows the SIDER reporting.
