# Supplementary material for: The side effect profile of Clozapine in real world data of three large mental health hospitals
Source: PLoS One. 2020 Dec 8;15(12):e0243437. doi: 10.1371/journal.pone.0243437 (PMC7723266; doi:10.1371/journal.pone.0243437)
Supplement: S2 Table — (PDF) [file pone.0243437.s002.pdf]

# Chi Square Statistics (χ2)

| ADR | Cohort | Trust | Months: One Month Later | Months: Two Months Later | Months: Three Months Later |
|---|---|---|---|---|---|
| **Abdominalpain** | Gender (1 df) | SLAM | 2.30e-28 | 3.60e-30 | 1.73e+00 |
| | | C&I | 5.11e-02 | 1.16e-02 | 8.22e-02 |
| | | Oxford | 5.95e-03 | 7.89e-03 | 2.36e+00 |
| | Ethnicity (3 df) | SLAM | 1.03e+00 | 9.10e+00 | 1.03e+00 |
| | | C&I | 7.42e-01 | 1.06e+00 | 7.96e-01 |
| | | Oxford | 3.82e+00 | 2.18e+00 | 1.60e+00 |
| | Agegroup (7 df) | SLAM | 6.31e+00 | 4.63e+00 | 2.34e+00 |
| | | C&I | 8.05e+00 | 2.75e+00 | 6.69e+00 |
| | Hospital Admissions(1 df) | SLAM | 6.84e+01 | 1.67e+01 | 2.46e+01 |
| | | C&I | 1.20e+00 | 1.33e+01 | 1.37e-01 |
| | Smoking Status (1 df) | SLAM | 2.55e+01 | 2.00e+01 | 2.65e+01 |
| | | C&I | 1.17e+00 | 3.03e+00 | 2.59e+00 |
| **Agitation** | Gender (1 df) | SLAM | 5.51e+00 | 1.03e+00 | 1.62e-01 |
| | | C&I | 2.27e+00 | 2.73e-01 | 5.39e-01 |
| | | Oxford | 6.05e-03 | 1.29e-01 | 8.67e-01 |
| | Ethnicity (3 df) | SLAM | 7.58e+00 | 9.10e+00 | 5.45e+00 |
| | | C&I | 8.30e+00 | 2.35e+00 | 3.78e+00 |
| | | Oxford | 1.88e+00 | 9.56e-01 | 1.12e+00 |
| | Agegroup (7 df) | SLAM | 4.86e+01 | 2.75e+01 | 4.57e+01 |
| | | C&I | 9.31e+00 | 5.90e+00 | 2.14e+01 |
| | Hospital Admissions(1 df) | SLAM | 4.88e+02 | 2.98e+02 | 2.21e+02 |
| | | C&I | 7.66e+01 | 3.72e+01 | 1.04e+01 |
| | Smoking Status (1 df) | SLAM | 2.74e+02 | 1.62e+02 | 1.84e+02 |
| | | C&I | 7.65e+01 | 4.28e+01 | 3.10e+01 |
| **Akathisia** | Gender (1 df) | SLAM | 1.10e-02 | 2.20e+00 | 2.44e-27 |
| | | C&I | 1.29e-03 | 2.21e-31 | 9.60e-28 |
| | | Oxford | 1.62e-02 | 4.46e-31 | 1.53e-30 |
| | Ethnicity (3 df) | SLAM | 4.56e+00 | 3.37e+00 | 2.86e-01 |
| | | C&I | 1.39e+00 | 5.82e+00 | 1.17e+01 |
| | | Oxford | 1.47e+00 | 1.25e+00 | 1.04e+00 |
| | Agegroup (7 df) | SLAM | 1.06e+01 | 1.17e+01 | 2.89e+00 |
| | | C&I | 2.99e+00 | 4.47e+00 | 2.77e+00 |
| | Hospital Admissions(1 df) | SLAM | 1.97e+01 | 1.24e+01 | 3.91e+00 |
| | | C&I | 7.38e-31 | 1.12e+01 | 1.64e+00 |
| | Smoking Status (1 df) | SLAM | 4.12e+00 | 9.50e-01 | 8.16e+00 |
| | | C&I | 2.54e+00 | 3.76e-28 | 1.86e-28 |
| **Backache** | Gender (1 df) | SLAM | 5.62e+00 | 2.48e+00 | 1.69e-01 |
| | | C&I | 8.77e+00 | 1.41e+00 | 2.89e+00 |
| | | Oxford | 6.64e+00 | 7.63e-01 | 3.74e+00 |
| | Ethnicity (3 df) | SLAM | 1.08e+00 | 3.08e+00 | 5.53e+00 |
| | | C&I | 4.32e+00 | 1.96e+00 | 4.11e+00 |
| | | Oxford | 3.73e-01 | 2.84e+00 | 2.10e+00 |
| | Agegroup (7 df) | SLAM | 7.47e+00 | 2.05e+00 | 3.88e+00 |
| | | C&I | 1.63e+01 | 1.05e+01 | 2.07e+00 |
| | Hospital Admissions(1 df) | SLAM | 4.49e+01 | 3.12e+01 | 1.35e+01 |
| | | C&I | 1.19e+01 | 1.37e+01 | 4.09e-01 |
| | Smoking Status (1 df) | SLAM | 1.47e+01 | 1.78e+01 | 4.54e+00 |
| | | C&I | 8.05e+00 | 1.77e+00 | 3.40e+00 |
| **Blurredvision** | Gender (1 df) | SLAM | 6.90e+00 | 1.58e-03 | 4.76e-02 |

P Value
- <0.05
- >0.05

**Chi Square (χ2)** statistics are shown in the results and broken down by ADR, the cohort with a degree of freedom*, three trusts (SLaM, Camden & Islington and Oxford) and further broken down into three months after starting the drug Clozapine.
Adjustment for multiple comparisons: **Bonferroni**.
The mean difference is significant at the **0.05 level** (95% confidence interval for difference). The results in **Red** shows statistically significant p values.

## Chi Square Statistics (χ2)

P Value
<0.05
>0.05

| ADR | Cohort | Trust | Months: One Month Later | Two Months Later | Three Months Later |
|---|---|---|---|---|---|
| **Blurredvision** | Gender (1 df) | C&I | 1.50e-28 | 9.25e-28 | 2.48e-31 |
| | | Oxford | 9.21e-31 | 1.79e-02 | 1.83e-01 |
| | Ethnicity (3 df) | SLAM | 1.89e+00 | 1.94e+00 | 1.47e+00 |
| | | C&I | 1.46e+00 | 1.24e+00 | 1.66e+00 |
| | | Oxford | 1.80e+00 | 9.72e-01 | 1.25e+00 |
| | Agegroup (7 df) | SLAM | 7.69e+00 | 6.84e+00 | 1.01e+01 |
| | | C&I | 5.63e+00 | 5.95e+00 | 5.70e+00 |
| | Hospital Admissions(1 df) | SLAM | 1.03e+01 | 1.00e+01 | 2.02e+00 |
| | | C&I | 1.04e+00 | 2.71e-02 | 3.32e-01 |
| | Smoking Status (1 df) | SLAM | 1.83e-01 | 3.88e+00 | 3.48e+00 |
| | | C&I | 6.39e-01 | 1.02e-01 | 7.46e-02 |
| **Confusion** | Gender (1 df) | SLAM | 7.52e-01 | 2.90e-03 | 5.21e-01 |
| | | C&I | 3.74e-01 | 1.34e+00 | 2.82e+00 |
| | | Oxford | 2.89e+00 | 3.04e-02 | 2.11e+00 |
| | Ethnicity (3 df) | SLAM | 2.52e+00 | 3.59e+00 | 2.63e+00 |
| | | C&I | 6.07e+00 | 7.88e+00 | 3.02e+00 |
| | | Oxford | 4.92e+00 | 1.45e+00 | 1.05e+00 |
| | Agegroup (7 df) | SLAM | 8.89e+00 | 1.86e+01 | 6.20e+00 |
| | | C&I | 1.58e+01 | 3.45e+00 | 2.47e+00 |
| | Hospital Admissions(1 df) | SLAM | 1.09e+02 | 6.40e+01 | 3.95e+01 |
| | | C&I | 2.57e+01 | 1.67e+01 | 1.25e-01 |
| | Smoking Status (1 df) | SLAM | 5.90e+01 | 2.30e+01 | 3.41e+01 |
| | | C&I | 1.78e+01 | 6.22e+00 | 1.22e+01 |
| **Constipation** | Gender (1 df) | SLAM | 1.01e+01 | 2.08e+01 | 6.00e+00 |
| | | C&I | 7.94e-01 | 2.98e-29 | 4.92e+00 |
| | | Oxford | 2.14e+00 | 2.06e+00 | 2.06e+00 |
| | Ethnicity (3 df) | SLAM | 1.37e+01 | 8.75e+00 | 1.09e+00 |
| | | C&I | 4.91e+00 | 2.90e+00 | 1.46e+00 |
| | | Oxford | 1.38e+00 | 9.66e-01 | 2.34e+00 |
| | Agegroup (7 df) | SLAM | 1.43e+01 | 1.38e+01 | 1.20e+01 |
| | | C&I | 1.02e+01 | 2.30e+00 | 1.82e+00 |
| | Hospital Admissions(1 df) | SLAM | 5.01e+01 | 5.93e+01 | 1.92e+01 |
| | | C&I | 1.70e+01 | 4.80e+00 | 8.17e-01 |
| | Smoking Status (1 df) | SLAM | 1.26e+01 | 2.77e+01 | 8.78e+00 |
| | | C&I | 4.24e+00 | 2.73e+00 | 5.13e+00 |
| **Convulsion** | Gender (1 df) | SLAM | 1.15e-01 | 9.49e-01 | 6.47e-28 |
| | | C&I | 2.81e-02 | 8.94e-31 | 7.37e-02 |
| | | Oxford | 4.80e-29 | 2.36e+00 | 1.09e+00 |
| | Ethnicity (3 df) | SLAM | 3.53e+01 | 3.50e-01 | 1.84e+00 |
| | | C&I | 2.59e+00 | 7.24e-01 | 3.45e+00 |
| | | Oxford | 6.37e-01 | 4.08e+00 | 2.97e+00 |
| | Agegroup (7 df) | SLAM | 1.09e+01 | 9.62e+00 | 9.49e+00 |
| | | C&I | 8.64e+00 | 3.88e+00 | 3.30e+00 |
| | Hospital Admissions(1 df) | SLAM | 5.87e+01 | 2.64e+01 | 3.13e+01 |
| | | C&I | 2.46e-02 | 2.24e+00 | 2.15e-30 |
| | Smoking Status (1 df) | SLAM | 2.30e+01 | 1.00e+01 | 2.63e+00 |
| | | C&I | 4.25e-01 | 2.90e+00 | 3.09e-01 |
| **Diarrhoea** | Gender (1 df) | SLAM | 5.15e+00 | 1.19e-01 | 7.10e+00 |
| | | C&I | 2.65e-02 | 8.22e-02 | 1.41e+00 |

Chi Square (χ2) statistics are shown in the results and broken down by ADR, the cohort with a degree of freedom*, three trusts (SLaM, Camden & Islington and Oxford) and further broken down into three months after starting the drug Clozapine.
Adjustment for multiple comparisons: **Bonferroni**.
The mean difference is significant at the **0.05 level** (95% confidence interval for difference). The results in **Red** shows statistically significant p values.

## Chi Square Statistics (χ2)

| ADR | Cohort | Trust | Months: One Month Later | Two Months Later | Three Months Later |
|---|---|---|---|---|---|
| **Diarrhoea** | Gender (1 df) | Oxford | 6.68e+00 | 2.42e+00 | 6.10e+00 |
| | Ethnicity (3 df) | SLAM | 1.63e+00 | 3.71e+00 | 2.58e+00 |
| | | C&I | 3.58e+00 | 2.76e+00 | 2.24e+00 |
| | | Oxford | 2.95e+00 | 2.45e+00 | 2.76e+00 |
| | Agegroup (7 df) | SLAM | 1.96e+00 | 1.35e+00 | 5.70e+00 |
| | | C&I | 1.86e+00 | 3.63e+00 | 6.82e+00 |
| | Hospital Admissions(1 df) | SLAM | 3.72e+01 | 1.98e+01 | 2.30e+01 |
| | | C&I | 3.47e+00 | 2.34e+00 | 3.41e-01 |
| | Smoking Status (1 df) | SLAM | 2.94e+00 | 1.03e+01 | 3.32e+00 |
| | | C&I | 3.40e+00 | 6.67e+00 | 4.42e-02 |
| **Dizziness** | Gender (1 df) | SLAM | 1.25e+01 | 4.17e+00 | 2.13e+00 |
| | | C&I | 2.84e+00 | 3.66e+00 | 8.14e-01 |
| | | Oxford | 4.91e+00 | 2.85e+00 | 2.50e+00 |
| | Ethnicity (3 df) | SLAM | 3.80e+00 | 1.14e+01 | 1.83e+01 |
| | | C&I | 1.87e+00 | 3.47e+00 | 2.42e+00 |
| | | Oxford | 2.36e+00 | 3.18e+00 | 2.45e-01 |
| | Agegroup (7 df) | SLAM | 1.25e+01 | 5.22e+00 | 1.18e+01 |
| | | C&I | 5.88e+00 | 1.18e+01 | 6.99e+00 |
| | Hospital Admissions(1 df) | SLAM | 1.25e+02 | 5.70e+01 | 3.57e+01 |
| | | C&I | 2.08e+01 | 2.96e+01 | 1.65e+01 |
| | Smoking Status (1 df) | SLAM | 3.46e+01 | 2.54e+01 | 2.93e+01 |
| | | C&I | 1.70e+01 | 1.49e+01 | 8.96e+00 |
| **Drymouth** | Gender (1 df) | SLAM | 1.36e+00 | 4.13e+00 | 5.26e-02 |
| | | C&I | 4.60e-01 | 4.75e-01 | 2.47e+00 |
| | | Oxford | 3.32e-01 | 3.32e-30 | 2.37e+00 |
| | Ethnicity (3 df) | SLAM | 1.19e+01 | 4.01e+00 | 7.17e-01 |
| | | C&I | 1.92e+00 | 1.73e+00 | 9.71e-01 |
| | | Oxford | 2.50e+00 | 1.01e+00 | 1.79e+00 |
| | Agegroup (7 df) | SLAM | 8.32e+00 | 1.33e+01 | 9.32e+00 |
| | | C&I | 3.24e+00 | 7.54e+00 | 1.78e+00 |
| | Hospital Admissions(1 df) | SLAM | 2.14e+01 | 1.74e+01 | 7.12e+00 |
| | | C&I | 2.68e+00 | 5.93e-01 | 2.92e-01 |
| | Smoking Status (1 df) | SLAM | 6.51e+00 | 6.78e+00 | 1.56e+01 |
| | | C&I | 2.35e+00 | 1.20e+00 | 3.21e-28 |
| **Dyspepsia** | Gender (1 df) | SLAM | 4.28e-03 | 1.32e+00 | 4.83e-01 |
| | | C&I | 1.61e+00 | 2.70e-01 | 2.13e+00 |
| | | Oxford | 3.13e+00 | 1.36e+00 | 1.13e+00 |
| | Ethnicity (3 df) | SLAM | 3.73e+00 | 3.57e+00 | 2.16e+00 |
| | | C&I | 4.82e+00 | 4.88e+00 | 5.47e+00 |
| | | Oxford | 2.08e+00 | 3.01e+00 | 1.05e+00 |
| | Agegroup (7 df) | SLAM | 8.18e+00 | 2.14e+00 | 4.56e+00 |
| | | C&I | 5.06e+00 | 1.11e+01 | 5.64e+00 |
| | Hospital Admissions(1 df) | SLAM | 3.17e+01 | 5.53e+00 | 2.44e+01 |
| | | C&I | 4.10e+00 | 8.92e-01 | 9.80e+00 |
| | Smoking Status (1 df) | SLAM | 2.20e+01 | 1.32e+01 | 1.51e+01 |
| | | C&I | 6.50e+00 | 1.05e+00 | 3.94e+00 |
| **Enuresis** | Gender (1 df) | SLAM | 8.45e+00 | 1.19e+01 | 2.28e+00 |
| | | C&I | 8.94e-01 | 1.39e+00 | 1.50e-28 |
| | | Oxford | 2.43e-01 | 2.96e+00 | 5.18e+00 |

P Value
- <0.05 (red)
- >0.05 (blue)

**Chi Square (χ2)** statistics are shown in the results and broken down by ADR, the cohort with a degree of freedom*, three trusts (SLaM, Camden & Islington and Oxford) and further broken down into three months after starting the drug Clozapine.
Adjustment for multiple comparisons: **Bonferroni**.
The mean difference is significant at the **0.05 level** (95% confidence interval for difference). The results in **Red** shows statistically significant p values.

## Chi Square Statistics (χ2)

| ADR | Cohort | Trust | Months: One Month Later | Two Months Later | Three Months Later |
|---|---|---|---|---|---|
| Enuresis | Ethnicity (3 df) | SLAM | 4.10e+00 | 3.10e+00 | 1.15e+00 |
| | | C&I | 6.34e+00 | 1.59e+00 | 2.01e+00 |
| | | Oxford | 6.83e-01 | 8.87e-01 | 4.54e+00 |
| | Agegroup (7 df) | SLAM | 9.63e+00 | 3.61e+00 | 1.04e+01 |
| | | C&I | 8.15e+00 | 1.50e+01 | 1.29e+01 |
| | Hospital Admissions(1 df) | SLAM | 4.71e+01 | 2.63e+01 | 1.82e+01 |
| | | C&I | 4.10e+00 | 5.99e-01 | 3.86e+00 |
| | Smoking Status (1 df) | SLAM | 2.53e+01 | 8.64e+00 | 5.87e+00 |
| | | C&I | 2.76e+00 | 3.47e-29 | 4.05e-05 |
| Fatigue | Gender (1 df) | SLAM | 1.58e+01 | 1.30e+01 | 6.66e+00 |
| | | C&I | 1.79e+00 | 1.70e-01 | 1.12e+00 |
| | | Oxford | 4.58e+00 | 3.04e+00 | 7.76e-02 |
| | Ethnicity (3 df) | SLAM | 8.55e+00 | 4.80e+00 | 5.40e+00 |
| | | C&I | 4.42e+00 | 7.38e-01 | 6.18e+00 |
| | | Oxford | 1.89e+00 | 6.25e+00 | 8.28e-01 |
| | Agegroup (7 df) | SLAM | 5.51e+01 | 5.32e+01 | 4.80e+01 |
| | | C&I | 1.12e+01 | 4.34e+00 | 7.32e+00 |
| | Hospital Admissions(1 df) | SLAM | 3.23e+02 | 2.99e+02 | 2.47e+02 |
| | | C&I | 2.97e+01 | 3.37e+01 | 2.09e+01 |
| | Smoking Status (1 df) | SLAM | 1.84e+02 | 1.41e+02 | 1.48e+02 |
| | | C&I | 4.69e+01 | 4.66e+01 | 4.30e+01 |
| Feelingsick | Gender (1 df) | SLAM | 2.48e+01 | 1.88e+01 | 1.30e+01 |
| | | C&I | 8.95e-02 | 1.02e+00 | 2.02e-03 |
| | | Oxford | 6.77e-01 | 7.63e-01 | 3.43e+00 |
| | Ethnicity (3 df) | SLAM | 2.25e+00 | 2.23e+00 | 4.33e+00 |
| | | C&I | 2.07e+00 | 5.50e+00 | 2.64e+00 |
| | | Oxford | 7.57e-01 | 5.64e+00 | 2.08e+00 |
| | Agegroup (7 df) | SLAM | 3.65e+01 | 3.46e+01 | 2.17e+01 |
| | | C&I | 4.72e+00 | 8.55e+00 | 1.04e+01 |
| | Hospital Admissions(1 df) | SLAM | 9.59e+01 | 6.85e+01 | 4.86e+01 |
| | | C&I | 1.55e+01 | 1.89e+00 | 2.20e+00 |
| | Smoking Status (1 df) | SLAM | 3.18e+01 | 3.10e+01 | 1.93e+01 |
| | | C&I | 1.53e+01 | 9.13e+00 | 1.08e+01 |
| Fever | Gender (1 df) | SLAM | 8.09e+00 | 4.06e+00 | 7.88e-02 |
| | | C&I | 9.09e-31 | 2.75e+00 | 2.48e-31 |
| | | Oxford | 1.06e-28 | 1.09e+00 | 8.99e-02 |
| | Ethnicity (3 df) | SLAM | 2.17e+00 | 1.86e+00 | 1.67e+00 |
| | | C&I | 3.91e+00 | 2.12e-01 | 3.90e+00 |
| | | Oxford | 1.10e+00 | 6.84e-01 | 1.79e+00 |
| | Agegroup (7 df) | SLAM | 2.82e+00 | 5.06e+00 | 7.89e+00 |
| | | C&I | 9.85e+00 | 6.19e+00 | 1.55e+01 |
| | Hospital Admissions(1 df) | SLAM | 4.69e+01 | 1.21e+01 | 2.63e+01 |
| | | C&I | 2.07e+01 | 8.92e-01 | 2.67e-28 |
| | Smoking Status (1 df) | SLAM | 1.80e+01 | 1.69e+01 | 1.32e+01 |
| | | C&I | 5.43e+00 | 4.47e+00 | 7.46e-02 |
| Headache | Gender (1 df) | SLAM | 1.20e+01 | 2.16e+00 | 1.91e+00 |
| | | C&I | 5.32e-01 | 4.48e-02 | 3.60e-04 |
| | | Oxford | 5.15e-03 | 9.01e-02 | 2.52e-02 |
| | Ethnicity (3 df) | SLAM | 2.77e+00 | 2.12e+00 | 2.82e+00 |

P Value
- <0.05 (red)
- >0.05 (blue)

**Chi Square (χ2)** statistics are shown in the results and broken down by ADR, the cohort with a degree of freedom*, three trusts (SLaM, Camden & Islington and Oxford) and further broken down into three months after starting the drug Clozapine.
Adjustment for multiple comparisons: **Bonferroni**.
The mean difference is significant at the **0.05 level** (95% confidence interval for difference). The results in **Red** shows statistically significant p values.

## Chi Square Statistics (χ2)

| ADR | Cohort | Trust | Months: One Month Later | Two Months Later | Three Months Later |
|---|---|---|---|---|---|
| **Headache** | Ethnicity (3 df) | C&I | 1.44e+00 | 4.46e+00 | 2.36e+00 |
| | | Oxford | 3.99e+00 | 1.66e+00 | 6.78e+00 |
| | Agegroup (7 df) | SLAM | 1.50e+01 | 6.23e+00 | 1.74e+01 |
| | | C&I | 3.79e+00 | 1.06e+01 | 1.62e+01 |
| | Hospital Admissions(1 df) | SLAM | 8.18e+01 | 2.30e+01 | 3.28e+01 |
| | | C&I | 1.04e+01 | 3.21e+00 | 4.43e+00 |
| | Smoking Status (1 df) | SLAM | 4.81e+01 | 2.91e+01 | 4.09e+01 |
| | | C&I | 9.46e+00 | 3.76e+00 | 4.07e+00 |
| **Hyperprolactinaemia** | Gender (1 df) | SLAM | 1.87e+01 | 9.67e+00 | 1.54e+00 |
| | | C&I | 3.41e+00 | 5.17e+00 | 2.33e+00 |
| | | Oxford | 1.30e+00 | 1.61e+00 | 1.86e+00 |
| | Ethnicity (3 df) | SLAM | 2.55e+00 | 1.81e+00 | 1.62e+00 |
| | | C&I | 9.13e+00 | 8.72e-01 | 1.24e+00 |
| | | Oxford | 1.44e+00 | 9.54e-01 | 7.01e+00 |
| | Agegroup (7 df) | SLAM | 2.01e+01 | 2.51e+01 | 2.99e+01 |
| | | C&I | 9.53e+00 | 2.26e+00 | 2.95e+00 |
| | Hospital Admissions(1 df) | SLAM | 4.48e+01 | 2.85e+01 | 1.89e+01 |
| | | C&I | 9.72e+00 | 1.91e-01 | 9.74e-31 |
| | Smoking Status (1 df) | SLAM | 1.07e+00 | 4.29e-01 | 1.73e+00 |
| | | C&I | 5.02e+00 | 5.97e-01 | 4.01e-01 |
| **Hypersalivation** | Gender (1 df) | SLAM | 2.25e+00 | 1.08e+00 | 7.53e-01 |
| | | C&I | 2.20e-02 | 8.56e-01 | 8.93e-01 |
| | | Oxford | 2.61e+00 | 1.10e-01 | 1.90e+00 |
| | Ethnicity (3 df) | SLAM | 2.14e+00 | 2.89e+00 | 1.76e+00 |
| | | C&I | 3.88e+00 | 2.31e+00 | 3.88e+00 |
| | | Oxford | 1.45e+00 | 1.17e+00 | 1.68e+00 |
| | Agegroup (7 df) | SLAM | 1.44e+01 | 2.48e+00 | 2.05e+01 |
| | | C&I | 7.09e+00 | 9.81e+00 | 5.35e+00 |
| | Hospital Admissions(1 df) | SLAM | 5.96e+01 | 7.55e+01 | 4.34e+01 |
| | | C&I | 1.39e-01 | 3.56e+00 | 4.83e-01 |
| | Smoking Status (1 df) | SLAM | 1.27e+01 | 1.08e+01 | 1.14e+01 |
| | | C&I | 1.69e+00 | 2.40e+00 | 5.23e+00 |
| **Hypertension** | Gender (1 df) | SLAM | 1.61e+00 | 5.66e-01 | 2.52e-03 |
| | | C&I | 1.06e-01 | 1.59e-01 | 7.26e-29 |
| | | Oxford | 2.62e-01 | 2.76e-01 | 1.37e-02 |
| | Ethnicity (3 df) | SLAM | 1.80e+00 | 2.87e+00 | 3.69e+00 |
| | | C&I | 2.75e+00 | 2.42e+00 | 3.20e+00 |
| | | Oxford | 1.91e+01 | 2.67e+00 | 4.17e+00 |
| | Agegroup (7 df) | SLAM | 1.31e+01 | 2.43e+01 | 7.58e+00 |
| | | C&I | 1.98e+01 | 5.27e+00 | 3.35e+00 |
| | Hospital Admissions(1 df) | SLAM | 2.21e+01 | 2.32e+01 | 1.84e+01 |
| | | C&I | 6.75e+00 | 2.58e+00 | 8.92e-01 |
| | Smoking Status (1 df) | SLAM | 1.18e+01 | 1.24e+01 | 1.67e+01 |
| | | C&I | 3.93e-01 | 4.07e+00 | 2.28e-01 |
| **Hypotension** | Gender (1 df) | SLAM | 8.84e+00 | 8.51e-02 | 1.17e-02 |
| | | C&I | 2.33e+00 | 2.03e-01 | 1.53e+00 |
| | | Oxford | 3.77e+00 | 3.13e+00 | 3.74e+00 |
| | Ethnicity (3 df) | SLAM | 1.09e+01 | 8.11e+00 | 1.71e+00 |
| | | C&I | 6.57e+00 | 2.89e+00 | 4.23e+00 |

P Value
- <0.05 (red)
- >0.05 (blue)

**Chi Square (χ2)** statistics are shown in the results and broken down by ADR, the cohort with a degree of freedom*, three trusts (SLaM, Camden & Islington and Oxford) and further broken down into three months after starting the drug Clozapine.
Adjustment for multiple comparisons: **Bonferroni**.
The mean difference is significant at the **0.05 level** (95% confidence interval for difference). The results in **Red** shows statistically significant p values.

## Chi Square Statistics (χ2)

| ADR | Cohort | Trust | Months<br>One Month Later | <br>Two Months Later | <br>Three Months Later |
|---|---|---|---|---|---|
| Hypotension | Ethnicity (3 df) | Oxford | 4.68e+00 | 2.23e+00 | 2.10e+00 |
| | Agegroup (7 df) | SLAM | 1.94e+01 | 1.98e+01 | 3.35e+01 |
| | | C&I | 7.03e+00 | 7.87e+00 | 2.27e+01 |
| | Hospital Admissions(1 df) | SLAM | 5.90e+01 | 2.01e+01 | 8.74e+00 |
| | | C&I | 1.90e+00 | 7.24e+00 | 2.33e-30 |
| | Smoking Status (1 df) | SLAM | 6.60e+00 | 6.35e+00 | 2.30e+00 |
| | | C&I | 6.26e-01 | 8.52e-03 | 5.19e-01 |
| Insomnia | Gender (1 df) | SLAM | 9.31e-01 | 1.09e+00 | 1.93e+00 |
| | | C&I | 1.29e+00 | 5.96e-01 | 1.45e+00 |
| | | Oxford | 7.50e+00 | 1.07e+00 | 4.84e-01 |
| | Ethnicity (3 df) | SLAM | 2.73e+00 | 2.72e+00 | 1.55e+00 |
| | | C&I | 5.79e-01 | 6.41e-01 | 2.72e-01 |
| | | Oxford | 1.29e+00 | 2.43e+00 | 3.34e+00 |
| | Agegroup (7 df) | SLAM | 1.04e+01 | 8.15e+00 | 1.60e+01 |
| | | C&I | 4.93e+00 | 1.11e+01 | 4.23e+00 |
| | Hospital Admissions(1 df) | SLAM | 6.74e+01 | 2.18e+01 | 5.75e+00 |
| | | C&I | 2.41e+01 | 4.46e+00 | 1.04e-01 |
| | Smoking Status (1 df) | SLAM | 3.73e+01 | 1.58e+01 | 9.61e+00 |
| | | C&I | 1.24e+01 | 4.40e+00 | 4.92e+00 |
| Nausea | Gender (1 df) | SLAM | 5.84e+00 | 2.89e+00 | 9.62e+00 |
| | | C&I | 7.29e-01 | 1.11e+00 | 1.11e+00 |
| | | Oxford | 3.23e+00 | 3.20e-01 | 6.76e-01 |
| | Ethnicity (3 df) | SLAM | 5.14e+00 | 6.18e+00 | 7.44e-01 |
| | | C&I | 7.45e-01 | 3.40e+00 | 1.87e+00 |
| | | Oxford | 3.67e+00 | 5.20e+00 | 1.18e+00 |
| | Agegroup (7 df) | SLAM | 4.89e+00 | 8.34e+00 | 1.17e+01 |
| | | C&I | 3.86e-01 | 6.30e+00 | 2.45e+00 |
| | Hospital Admissions(1 df) | SLAM | 2.49e+01 | 1.52e+01 | 5.65e+00 |
| | | C&I | 6.78e+00 | 6.60e-01 | 1.02e-01 |
| | Smoking Status (1 df) | SLAM | 8.45e+00 | 2.45e+00 | 5.50e+00 |
| | | C&I | 2.55e+00 | 1.60e+00 | 1.95e-29 |
| Neutropenia | Gender (1 df) | SLAM | 1.92e+00 | 6.85e-01 | 1.00e-01 |
| | | C&I | 7.35e-01 | 2.48e-31 | 7.37e-02 |
| | Ethnicity (3 df) | SLAM | 2.07e+00 | 2.86e+00 | 2.37e+00 |
| | | C&I | 7.52e+00 | 1.04e+01 | 3.47e+00 |
| | Agegroup (7 df) | SLAM | 2.27e+01 | 8.12e+00 | 5.55e+00 |
| | | C&I | 2.46e+00 | 2.17e+00 | 2.61e+00 |
| | Hospital Admissions(1 df) | SLAM | 2.69e+01 | 7.78e+00 | 1.37e+01 |
| | | C&I | 3.14e-01 | 3.32e-01 | 2.15e-30 |
| | Smoking Status (1 df) | SLAM | 5.63e+00 | 7.44e+00 | 6.43e+00 |
| | | C&I | 1.46e+00 | 7.46e-02 | 3.76e-28 |
| Rash | Gender (1 df) | SLAM | 1.77e+00 | 1.40e+00 | 1.85e+00 |
| | | C&I | 2.83e-01 | 1.00e-01 | 8.94e-31 |
| | | Oxford | 1.13e+00 | 8.44e-01 | 8.72e-01 |
| | Ethnicity (3 df) | SLAM | 1.26e+01 | 1.65e+00 | 4.36e-01 |
| | | C&I | 2.07e+00 | 5.93e+00 | 5.56e+00 |
| | | Oxford | 1.42e+00 | 2.36e+00 | 2.92e+00 |
| | Agegroup (7 df) | SLAM | 2.41e+00 | 1.80e+00 | 2.72e+00 |
| | | C&I | 5.09e+00 | 1.74e+00 | 6.79e+00 |

P Value
- <0.05 (red)
- >0.05 (blue)

**Chi Square (χ2)** statistics are shown in the results and broken down by ADR, the cohort with a degree of freedom*, three trusts (SLaM, Camden & Islington and Oxford) and further broken down into three months after starting the drug Clozapine.
Adjustment for multiple comparisons: **Bonferroni**.
The mean difference is significant at the **0.05 level** (95% confidence interval for difference). The results in **Red** shows statistically significant p values.

## Chi Square Statistics (χ2)

| ADR | Cohort | Trust | Months: One Month Later | Two Months Later | Three Months Later |
|---|---|---|---|---|---|
| Rash | Hospital Admissions(1 df) | SLAM | 2.33e+01 | 2.56e+01 | 3.69e+01 |
| | | C&I | 1.85e+00 | 9.16e-01 | 1.99e-03 |
| | Smoking Status (1 df) | SLAM | 5.10e+00 | 1.14e+01 | 1.03e+01 |
| | | C&I | 4.92e+00 | 4.78e+00 | 1.20e+00 |
| Sedation | Gender (1 df) | SLAM | 4.73e+00 | 4.86e+00 | 7.36e+00 |
| | | C&I | 1.30e+00 | 6.86e-02 | 1.23e+00 |
| | | Oxford | 6.74e-02 | 0.00e+00 | 4.26e-01 |
| | Ethnicity (3 df) | SLAM | 7.22e+00 | 6.71e+00 | 4.07e+00 |
| | | C&I | 5.31e+00 | 2.72e+00 | 2.13e+00 |
| | | Oxford | 2.23e+00 | 6.06e+00 | 2.41e+00 |
| | Agegroup (7 df) | SLAM | 4.02e+01 | 5.62e+01 | 3.95e+01 |
| | | C&I | 7.92e+00 | 6.59e+00 | 1.53e+01 |
| | Hospital Admissions(1 df) | SLAM | 3.69e+02 | 2.35e+02 | 2.24e+02 |
| | | C&I | 4.49e+01 | 3.31e+01 | 5.81e+00 |
| | Smoking Status (1 df) | SLAM | 1.76e+02 | 1.28e+02 | 1.09e+02 |
| | | C&I | 2.25e+01 | 2.87e+01 | 2.31e+01 |
| Shaking | Gender (1 df) | SLAM | 7.06e-01 | 1.11e-01 | 6.44e-01 |
| | | C&I | 9.31e-31 | 0.00e+00 | 4.83e-01 |
| | | Oxford | 1.51e-01 | 1.35e-29 | 4.98e+00 |
| | Ethnicity (3 df) | SLAM | 6.41e+00 | 7.27e+00 | 8.68e+00 |
| | | C&I | 4.35e+00 | 2.70e+00 | 6.57e+00 |
| | | Oxford | 2.44e+00 | 3.07e+00 | 3.82e+00 |
| | Agegroup (7 df) | SLAM | 2.87e+01 | 1.71e+01 | 3.25e+01 |
| | | C&I | 1.70e+01 | 6.91e+00 | 2.61e+01 |
| | Hospital Admissions(1 df) | SLAM | 7.64e+01 | 4.60e+01 | 3.35e+01 |
| | | C&I | 6.75e-02 | 3.10e-01 | 4.19e+00 |
| | Smoking Status (1 df) | SLAM | 2.50e+01 | 1.92e+01 | 2.81e+01 |
| | | C&I | 2.98e+00 | 1.17e+00 | 2.92e+00 |
| Stomachpain | Gender (1 df) | SLAM | 1.42e+01 | 3.27e+00 | 1.82e+01 |
| | | C&I | 5.96e-01 | 2.00e+00 | 4.75e-01 |
| | | Oxford | 1.37e-02 | 1.53e-30 | 6.20e-01 |
| | Ethnicity (3 df) | SLAM | 1.86e+00 | 1.98e+00 | 4.44e-01 |
| | | C&I | 6.94e+00 | 5.61e+00 | 3.55e+00 |
| | | Oxford | 2.32e+00 | 1.04e+00 | 1.04e+00 |
| | Agegroup (7 df) | SLAM | 5.32e+00 | 4.87e+00 | 6.92e+00 |
| | | C&I | 2.40e+00 | 9.41e+00 | 4.61e+00 |
| | Hospital Admissions(1 df) | SLAM | 3.64e+01 | 2.36e+01 | 1.45e+01 |
| | | C&I | 4.46e+00 | 4.19e+00 | 1.99e-03 |
| | Smoking Status (1 df) | SLAM | 2.91e+01 | 1.53e+01 | 6.77e+00 |
| | | C&I | 6.67e+00 | 2.92e+00 | 2.90e+00 |
| Sweating | Gender (1 df) | SLAM | 2.89e-03 | 3.76e+00 | 1.67e-01 |
| | | C&I | 1.49e+00 | 2.74e-01 | 9.02e-01 |
| | | Oxford | 1.13e-02 | 1.62e-02 | 6.10e-01 |
| | Ethnicity (3 df) | SLAM | 6.44e+00 | 2.71e+00 | 7.17e-01 |
| | | C&I | 3.66e+00 | 1.03e+00 | 1.01e+00 |
| | | Oxford | 1.50e+00 | 4.53e+00 | 3.57e+00 |
| | Agegroup (7 df) | SLAM | 8.64e+00 | 5.83e+00 | 5.06e+00 |
| | | C&I | 5.97e+00 | 5.04e+00 | 3.74e+00 |
| | Hospital | SLAM | 3.40e+01 | 8.37e+00 | 1.33e+01 |

P Value
- <0.05
- >0.05

**Chi Square (χ2)** statistics are shown in the results and broken down by ADR, the cohort with a degree of freedom*, three trusts (SLaM, Camden & Islington and Oxford) and further broken down into three months after starting the drug Clozapine.
Adjustment for multiple comparisons: **Bonferroni**.
The mean difference is significant at the **0.05 level** (95% confidence interval for difference). The results in **Red** shows statistically significant p values.

# Chi Square Statistics (χ2)

| ADR | Cohort | Trust | Months: One Month Later | Two Months Later | Three Months Later |
|---|---|---|---|---|---|
| **Sweating** | Admissions(1 df) | C&I | 4.19e+00 | 2.24e+00 | 2.94e+00 |
| | Smoking Status (1 df) | SLAM | 1.32e+01 | 1.71e+01 | 1.91e+01 |
| | | C&I | 2.92e+00 | 1.20e+00 | 8.38e-01 |
| **Tachycardia** | Gender (1 df) | SLAM | 1.88e+00 | 3.93e-02 | 5.66e-01 |
| | | C&I | 5.16e-29 | 6.12e-29 | 5.54e-02 |
| | | Oxford | 1.45e-30 | 4.93e-31 | 2.52e-02 |
| | Ethnicity (3 df) | SLAM | 3.11e+00 | 3.99e+00 | 5.13e+00 |
| | | C&I | 1.08e+01 | 6.00e-01 | 6.43e+00 |
| | | Oxford | 2.11e+00 | 6.45e+00 | 5.28e+00 |
| | Agegroup (7 df) | SLAM | 4.57e+01 | 4.79e+01 | 5.67e+01 |
| | | C&I | 1.24e+01 | 3.86e+00 | 7.55e+00 |
| | Hospital Admissions(1 df) | SLAM | 1.58e+02 | 1.20e+02 | 6.84e+01 |
| | | C&I | 2.07e+01 | 1.15e+01 | 3.56e+00 |
| | Smoking Status (1 df) | SLAM | 4.79e+01 | 4.20e+01 | 2.39e+01 |
| | | C&I | 1.13e+01 | 2.88e+00 | 3.59e+00 |
| **Tremor** | Gender (1 df) | SLAM | 2.33e-01 | 2.79e-02 | 2.12e-04 |
| | | C&I | 0.00e+00 | 0.00e+00 | 2.74e-01 |
| | Ethnicity (3 df) | SLAM | 6.68e+00 | 1.07e+00 | 3.55e+00 |
| | | C&I | 3.78e+00 | 2.00e+00 | 1.05e+00 |
| | Agegroup (7 df) | SLAM | 1.17e+01 | 1.18e+01 | 7.78e+00 |
| | | C&I | 6.00e+00 | 2.97e+00 | 6.81e+00 |
| | Hospital Admissions(1 df) | SLAM | 3.48e+01 | 2.90e+01 | 2.78e+01 |
| | | C&I | 7.39e+00 | 9.16e-01 | 2.24e+00 |
| | Smoking Status (1 df) | SLAM | 4.73e+00 | 5.47e+00 | 9.27e+00 |
| | | C&I | 1.17e+00 | 7.85e-02 | 5.35e+00 |
| **Vomiting** | Gender (1 df) | SLAM | 2.72e-01 | 4.06e+00 | 1.83e+00 |
| | | C&I | 3.65e-31 | 8.73e-01 | 1.62e+00 |
| | | Oxford | 3.78e-02 | 2.11e+00 | 2.43e-29 |
| | Ethnicity (3 df) | SLAM | 2.56e+00 | 2.08e+00 | 1.28e+00 |
| | | C&I | 3.97e+00 | 4.11e+00 | 5.11e+00 |
| | | Oxford | 6.77e-01 | 4.90e-01 | 3.67e+00 |
| | Agegroup (7 df) | SLAM | 1.83e+01 | 1.59e+01 | 2.28e+01 |
| | | C&I | 6.31e+00 | 8.01e+00 | 5.81e+00 |
| | Hospital Admissions(1 df) | SLAM | 2.13e+01 | 3.82e+01 | 3.49e+01 |
| | | C&I | 3.98e+00 | 1.52e-01 | 3.69e-01 |
| | Smoking Status (1 df) | SLAM | 1.59e+01 | 2.07e+01 | 9.08e+00 |
| | | C&I | 1.02e+01 | 3.36e+00 | 2.55e+00 |
| **Weightgain** | Gender (1 df) | SLAM | 9.08e+00 | 1.13e+01 | 4.76e+00 |
| | | C&I | 2.24e+00 | 4.57e+00 | 6.80e-03 |
| | | Oxford | 3.82e-01 | 1.15e+00 | 1.51e-01 |
| | Ethnicity (3 df) | SLAM | 5.03e+00 | 2.80e+00 | 2.14e+00 |
| | | C&I | 1.18e+00 | 1.49e-01 | 2.64e+00 |
| | | Oxford | 2.53e+00 | 3.96e+00 | 4.36e+00 |
| | Agegroup (7 df) | SLAM | 5.71e+01 | 4.43e+01 | 3.87e+01 |
| | | C&I | 1.96e+00 | 1.44e+01 | 6.11e+00 |
| | Hospital Admissions(1 df) | SLAM | 7.46e+01 | 7.03e+01 | 7.98e+01 |
| | | C&I | 2.75e+00 | 6.26e-05 | 3.62e+00 |
| | Smoking Status (1 df) | SLAM | 2.49e+01 | 3.71e+01 | 1.99e+01 |
| | | C&I | 3.82e+00 | 7.57e+00 | 8.57e+00 |

P Value
- <0.05
- >0.05

**Chi Square (χ2)** statistics are shown in the results and broken down by ADR, the cohort with a degree of freedom*, three trusts (SLaM, Camden & Islington and Oxford) and further broken down into three months after starting the drug Clozapine.
Adjustment for multiple comparisons: **Bonferroni**.
The mean difference is significant at the **0.05 level** (95% confidence interval for difference). The results in **Red** shows statistically significant p values.
