# Supplementary material for: The side effect profile of Clozapine in real world data of three large mental health hospitals
Source: PLoS One. 2020 Dec 8;15(12):e0243437. doi: 10.1371/journal.pone.0243437 (PMC7723266; doi:10.1371/journal.pone.0243437)
Supplement: S3 Table — (PDF) [file pone.0243437.s003.pdf]

Combine Analysis

ADR	Cohort	Months: One Month Later	Two Months Later	Three Months Later
Abdominalpain	Gender	1.79e-29	2.45e-29	4.09e+00
	Ethnicity	2.26e+00	5.76e+00	2.40e-01
	Agegroup	1.60e+01	5.64e+00	6.85e+00
	Hospital Admission	7.90e+01	3.10e+01	2.57e+01
	Smoking Status	2.52e+01	2.29e+01	2.90e+01
Agitation	Gender	1.14e+00	7.73e-01	9.86e-02
	Ethnicity	1.44e+01	7.62e+00	6.53e+00
	Agegroup	5.96e+01	3.63e+01	7.00e+01
	Hospital Admission	5.58e+02	3.37e+02	2.35e+02
	Smoking Status	3.49e+02	2.04e+02	2.10e+02
Akathisia	Gender	9.11e-03	1.28e+00	6.46e-28
	Ethnicity	3.80e+00	1.63e+00	1.53e+00
	Agegroup	1.33e+01	1.14e+01	1.82e+00
	Hospital Admission	2.01e+01	2.27e+01	7.08e+00
	Smoking Status	6.34e+00	8.24e-01	6.83e+00
Backache	Gender	2.03e+01	5.47e+00	4.79e+00
	Ethnicity	8.09e-01	2.01e-01	7.57e-01
	Agegroup	1.88e+01	7.07e+00	4.71e+00
	Hospital Admission	5.48e+01	4.47e+01	1.27e+01
	Smoking Status	2.35e+01	1.98e+01	8.47e+00
Blurredvision	Gender	5.78e+00	1.55e-31	3.90e-01
	Ethnicity	1.93e+00	1.00e+00	3.22e+00
	Agegroup	5.85e+00	8.72e+00	9.92e+00
	Hospital Admission	1.40e+01	1.37e+01	8.08e-01
	Smoking Status	6.59e-01	4.46e+00	3.94e+00
Confusion	Gender	1.14e+00	6.30e-02	5.81e-01
	Ethnicity	8.71e+00	3.87e+00	4.01e+00
	Agegroup	1.19e+01	2.23e+01	6.62e+00
	Hospital Admission	1.35e+02	8.41e+01	3.66e+01
	Smoking Status	7.72e+01	2.94e+01	4.68e+01
Constipation	Gender	1.33e+01	1.97e+01	1.25e+01
	Ethnicity	8.47e+00	2.99e+00	2.18e+00
	Agegroup	1.81e+01	1.38e+01	8.47e+00
	Hospital Admission	6.69e+01	7.36e+01	2.51e+01
	Smoking Status	1.71e+01	2.92e+01	1.27e+01
Convulsion	Gender	3.72e-03	1.50e-03	2.90e-01
	Ethnicity	1.90e+01	4.94e-01	2.62e+00
	Agegroup	1.62e+01	7.99e+00	1.09e+01
	Hospital Admission	6.68e+01	3.61e+01	3.69e+01
	Smoking Status	2.16e+01	1.25e+01	2.82e+00
Diarrhoea	Gender	1.00e+01	1.63e+00	1.53e+01
	Ethnicity	2.20e+00	2.03e+00	4.86e+00
	Agegroup	1.60e+00	1.54e+00	4.51e+00
	Hospital Admission	4.56e+01	2.27e+01	1.35e+01
	Smoking Status	5.50e+00	1.72e+01	1.82e+00
Dizziness	Gender	2.11e+01	1.10e+01	5.35e+00
	Ethnicity	4.96e-01	6.24e+00	5.63e+00

P value
- <0.05 (red)
- >0.05 (blue)

Chi Square (χ2) statistics are shown i in the results and broken down into ADR, cohort and further broken down into three months after starting the drug Clozapine.
Adjustment for multiple comparisons: **Bonferroni**.
The mean difference is significant at the **0.05 level** (95% confidence interval for difference). The results in **Red** shows statistically significant p values.

Combine Analysis

ADR	Cohort	Months<br>One Month Later	Two Months Later	Three Months Later
Dizziness	Agegroup	1.63e+01	9.25e+00	1.35e+01
	Hospital Admission	1.37e+02	8.03e+01	5.37e+01
	Smoking Status	5.23e+01	4.04e+01	3.82e+01
Drymouth	Gender	4.68e-02	4.21e+00	2.52e+00
	Ethnicity	5.92e+00	1.80e+00	2.13e-01
	Agegroup	1.15e+01	1.49e+01	1.13e+01
	Hospital Admission	2.57e+01	2.15e+01	1.08e+01
	Smoking Status	9.23e+00	8.20e+00	1.28e+01
Dyspepsia	Gender	3.42e-02	1.47e+00	1.89e-01
	Ethnicity	9.22e-01	3.23e+00	2.81e+00
	Agegroup	1.04e+01	6.93e+00	8.02e+00
	Hospital Admission	3.49e+01	7.45e+00	3.68e+01
	Smoking Status	2.97e+01	1.44e+01	1.95e+01
Enuresis	Gender	9.51e+00	1.72e+01	6.12e+00
	Ethnicity	2.72e+00	1.32e+00	4.69e-01
	Agegroup	9.98e+00	4.88e+00	1.28e+01
	Hospital Admission	5.03e+01	3.41e+01	2.78e+01
	Smoking Status	2.82e+01	6.90e+00	5.34e+00
Fatigue	Gender	2.21e+01	1.13e+01	7.24e+00
	Ethnicity	7.31e+00	7.29e+00	5.71e+00
	Agegroup	6.58e+01	6.03e+01	5.82e+01
	Hospital Admission	3.42e+02	3.41e+02	2.67e+02
	Smoking Status	2.30e+02	1.84e+02	1.89e+02
Feelingsick	Gender	2.06e+01	1.92e+01	1.31e+01
	Ethnicity	2.48e+00	3.45e+00	3.90e+00
	Agegroup	4.37e+01	4.54e+01	2.98e+01
	Hospital Admission	1.20e+02	7.68e+01	5.15e+01
	Smoking Status	4.53e+01	3.91e+01	2.94e+01
Fever	Gender	5.80e+00	8.37e+00	2.26e-01
	Ethnicity	7.03e+00	3.29e+00	4.87e+00
	Agegroup	3.78e+00	4.39e+00	1.46e+01
	Hospital Admission	7.18e+01	1.63e+01	3.10e+01
	Smoking Status	2.31e+01	2.15e+01	1.27e+01
Headache	Gender	5.39e+00	6.03e-01	1.67e+00
	Ethnicity	4.82e+00	1.05e+00	3.53e+00
	Agegroup	2.08e+01	1.23e+01	2.49e+01
	Hospital Admission	9.89e+01	2.91e+01	4.02e+01
	Smoking Status	5.70e+01	3.26e+01	4.45e+01
Hyperprolactinaemia	Gender	2.35e+01	1.73e+01	5.82e+00
	Ethnicity	5.77e+00	3.05e+00	1.50e+00
	Agegroup	2.63e+01	2.79e+01	2.85e+01
	Hospital Admission	5.45e+01	2.82e+01	1.63e+01
	Smoking Status	4.15e+00	9.76e-01	5.55e-01
Hypersalivation	Gender	3.51e-01	3.44e-01	2.84e+00
	Ethnicity	2.20e-01	1.72e+00	4.98e+00
	Agegroup	1.45e+01	8.77e+00	2.60e+01
	Hospital Admission	5.05e+01	9.24e+01	4.73e+01

P value
- <0.05 (red)
- >0.05 (blue)

Chi Square (χ2) statistics are shown i in the results and broken down into ADR, cohort and further broken down into three months after starting the drug Clozapine.
Adjustment for multiple comparisons: **Bonferroni**.
The mean difference is significant at the **0.05 level** (95% confidence interval for difference). The results in **Red** shows statistically significant p values.

Combine Analysis

P value
- <0.05 (red)
- >0.05 (blue)

ADR	Cohort	Months: One Month Later	Two Months Later	Three Months Later
Hypersalivation	Smoking Status	1.45e+01	1.23e+01	1.58e+01
Hypertension	Gender	1.02e+00	4.02e-01	2.27e-04
	Ethnicity	7.91e+00	5.94e+00	9.29e+00
	Agegroup	1.91e+01	1.41e+01	4.28e+00
	Hospital Admission	3.18e+01	2.80e+01	2.33e+01
	Smoking Status	1.14e+01	1.68e+01	1.58e+01
Hypotension	Gender	1.60e+01	2.07e+00	2.81e+00
	Ethnicity	5.36e+00	3.78e+00	1.90e+00
	Agegroup	2.29e+01	1.91e+01	4.06e+01
	Hospital Admission	6.33e+01	2.91e+01	8.56e+00
	Smoking Status	7.28e+00	5.74e+00	3.13e+00
Insomnia	Gender	1.84e+00	2.86e+00	8.83e-04
	Ethnicity	1.46e+00	4.60e-01	1.21e-01
	Agegroup	1.25e+01	7.69e+00	1.22e+01
	Hospital Admission	9.24e+01	3.24e+01	5.38e+00
	Smoking Status	4.99e+01	1.97e+01	1.52e+01
Nausea	Gender	1.02e+01	4.89e+00	1.23e+01
	Ethnicity	4.02e+00	7.55e+00	5.21e-01
	Agegroup	5.36e+00	1.08e+01	9.62e+00
	Hospital Admission	3.46e+01	1.86e+01	3.65e+00
	Smoking Status	1.12e+01	3.91e+00	4.38e+00
Rash	Gender	1.52e+00	2.87e+00	2.19e+00
	Ethnicity	6.22e+00	1.88e+00	1.13e+00
	Agegroup	4.07e+00	3.07e+00	2.73e+00
	Hospital Admission	2.29e+01	2.92e+01	3.20e+01
	Smoking Status	1.03e+01	1.64e+01	1.21e+01
Sedation	Gender	1.69e+00	3.65e+00	3.76e+00
	Ethnicity	1.45e+01	1.73e+01	1.02e+01
	Agegroup	5.43e+01	7.30e+01	6.45e+01
	Hospital Admission	4.16e+02	2.85e+02	2.33e+02
	Smoking Status	1.91e+02	1.52e+02	1.29e+02
Shaking	Gender	6.72e-01	9.07e-02	6.45e-30
	Ethnicity	3.09e+00	3.73e+00	4.84e+00
	Agegroup	4.40e+01	2.42e+01	4.78e+01
	Hospital Admission	7.70e+01	4.64e+01	4.40e+01
	Smoking Status	2.73e+01	1.98e+01	3.08e+01
Stomachpain	Gender	1.21e+01	5.66e+00	1.98e+01
	Ethnicity	5.74e+00	6.15e+00	4.29e+00
	Agegroup	4.94e+00	8.06e+00	8.00e+00
	Hospital Admission	4.53e+01	3.00e+01	1.61e+01
	Smoking Status	3.62e+01	1.89e+01	9.70e+00
Sweating	Gender	2.49e-01	3.64e+00	9.75e-02
	Ethnicity	7.29e+00	3.87e+00	1.26e+00
	Agegroup	1.07e+01	9.05e+00	5.97e+00
	Hospital Admission	4.31e+01	1.42e+01	1.89e+01
	Smoking Status	1.64e+01	1.81e+01	1.97e+01
Tachycardia	Gender	1.27e+00	5.82e-02	4.77e-01

Chi Square (χ2) statistics are shown i in the results and broken down into ADR, cohort and further broken down into three months after starting the drug Clozapine.
Adjustment for multiple comparisons: **Bonferroni**.
The mean difference is significant at the **0.05 level** (95% confidence interval for difference). The results in **Red** shows statistically significant p values.

Combine Analysis

ADR	Cohort	Months: One Month Later	Two Months Later	Three Months Later
Tachycardia	Ethnicity	9.86e+00	6.45e+00	1.34e+01
	Agegroup	6.00e+01	6.15e+01	6.99e+01
	Hospital Admission	1.89e+02	1.43e+02	7.73e+01
	Smoking Status	5.83e+01	4.26e+01	2.71e+01
Tremor	Gender	1.84e-01	1.88e-02	1.32e-01
	Ethnicity	6.38e+00	5.42e+00	6.67e+00
	Agegroup	1.51e+01	9.52e+00	1.19e+01
	Hospital Admission	4.64e+01	3.43e+01	3.43e+01
	Smoking Status	6.03e+00	5.33e+00	1.42e+01
Vomiting	Gender	7.20e-02	7.54e+00	3.11e+00
	Ethnicity	4.73e+00	3.34e+00	4.62e-01
	Agegroup	2.41e+01	2.40e+01	2.53e+01
	Hospital Admission	2.86e+01	3.86e+01	3.27e+01
	Smoking Status	2.49e+01	2.41e+01	1.22e+01
Weightgain	Gender	1.17e+01	1.67e+01	3.55e+00
	Ethnicity	3.75e+00	4.75e+00	5.07e+00
	Agegroup	5.43e+01	6.11e+01	5.27e+01
	Hospital Admission	8.05e+01	6.91e+01	9.23e+01
	Smoking Status	2.81e+01	4.37e+01	2.71e+01

P value
- <0.05 (red)
- >0.05 (blue)

Chi Square (χ2) statistics are shown i in the results and broken down into ADR, cohort and further broken down into three months after starting the drug Clozapine.
Adjustment for multiple comparisons: **Bonferroni**.
The mean difference is significant at the **0.05 level** (95% confidence interval for difference). The results in **Red** shows statistically significant p values.
