# Supplementary material for: The side effect profile of Clozapine in real world data of three large mental health hospitals
Source: PLoS One. 2020 Dec 8;15(12):e0243437. doi: 10.1371/journal.pone.0243437 (PMC7723266; doi:10.1371/journal.pone.0243437)
Supplement: Attachment — Submitted filename: Response to reviewers.docx [file pone.0243437.s004.docx]

Dear Editor,

Please find below responses to reviewer's comments for our submission ‘The side effect profile of Clozapine in real world data of three large mental health hospitals’ (PONE-D-20-04570). We hope that we have addressed all of the reviewer's’ concerns and feel that the paper has been substantially improved. Please see the attached copy of manuscript labeled ‘Revised manuscript with Track Changes’ and revised paper without track changes. Please find below the reviewers’ comments (in bold) along with the changes made and responses from our side.

**Reviewer has several concerns, though. The biggest concern is the lack of a replication cohort. As mentioned in a previous paragraph, in case of confusion as well as other subjective side effects (such as insomnia and dyspepsia), there might be a lot of discrepancies between departments and hospitals, as criteria for subjective side effects may differ among providers and specialties. Furthermore, there is a possibility of Berkson bias, as it is unclear to what extent data obtained in the UK are generalizable. It has been known that genetic architecture plays an important role in pathogenesis of psychotic disorders as well as in pharmacodynamics of clozapine, and it is not unlikely that obtained data may not be generalizable and/or replicated. Second, authors may want to clarify why 3-month period was selected for observation. Among most clinically significant side effects are agranulocytosis, myocarditis, and seizures, and these effects may take longer to develop.**

The study is replicated in three large mental health trusts, i) the South London and Maudsley (SLAM) NHS Foundation Trust, ii) the Camden and Islington (C&I) NHS Foundation Trust and iii) Oxford Health NHS Foundation Trust. In UK, Trust are group of hospitals that work together and deliver broad range of services. SLAM and C&I are based in London and have mixed population and Oxford is more homogeneous. Clinical recording behavior in different institute may differ, and it is dependent on the local preferences and procedures. We are using a robust ADE dictionary collated by our lead pharmacy partners which contain different terms and phrases to describe an ADE. Further, we have also evaluated our ADE detection pipeline in C&I trust to assess its generalisability. This is correct some of the ADRs may take longer to develop which also reflects in our results. Initially we aim to investigate side effects six months before and after starting the drug Clozapine. But number of patients were reduced significantly in all three trusts due to either the patients were not active within the Trust or they started their drug prescription from local GP practices. In this study, the cohort is defined to identify ADEs, medication and relevant comorbid factors such as diagnosis, demographics, hospital admission and smoking status. The approach is described in examining the baseline timeframe to achieve higher yield of eligible patients. For example, the diagnosis were determined by widening the timeframe to achieve higher yield. By Narrowing the timeframe, we achieve higher yield of available patients while longer timeframe included patients with missing data which may results in misclassification.

We also acknowledge that the results still may be specific to the UK in some way. SLAM and C&I trusts are based in London, the population are from very mixed backgrounds, and ethnicities and Oxford trust population are more homogenous. Therefore, we believe the results are specific to the UK but may not cover all UK specific concerns.

**There are also few minor comments. To what extend it is OK to use SIDER database as the only reference? The latest version of SIDER (4.1) was released in 2015, and data on side effects of clozapine listed there might be somewhat outdated. Could results be compared to an additional database? Furthermore, Table 2 appears to be redundant, numbers from this table likely could be given in the text in parentheses. Authors also may want to describe statistics in a more detail, especially how adjustments to potential confounders were made. Figures 1 and 2 seem to be redundant, and authors may consider removing them.**

We are using the latest SIDER (4.1) as of January 2018. These results can be compared with additional database, but no other sources are as comprehensive as SIDER.

We have removed the table and results are shown in text.

We have removed the Figure 1 and Figure 2.

We performed the statistical analysis individually to prevent the influence of confounders in our categorical variable such as age, gender, ethnicity, smoking and hospital admissions.
